# Asymmetric Ketocyanine Dyes with an Extended Polymethine Chain

**DOI:** 10.1002/open.202500096

**Published:** 2025-06-04

**Authors:** Sviatoslava O. Melnychuk, Sergii V. Popov, Serhii B. Babii, Andrii V. Kulinich

**Affiliations:** ^1^ Spectrum Info LLC 11 Myrnoho Panasa st., Office 2/28 Kyiv 01011 Ukraine; ^2^ Enamine Ltd. 78 Winston Churchill st. Kyiv 02094 Ukraine; ^3^ Institute of Organic Chemistry National Academy of Sciences of Ukraine 5 Akademika Kukharya st. Kyiv 02094 Ukraine

**Keywords:** absorption spectra, fluorescence, ketocyanines, polymethines, solvatochromism

## Abstract

A series of long‐chain ketocyanines (KCYs), in which chromophore asymmetry is achieved through the noncentral positioning of the acceptor carbonyl group and variation in the electron‐donating ability of the end groups, is synthesized via sequential condensation reactions and isolated with good preparative yields. The obtained dyes exhibit positive solvatochromism, with its range increasing as the donor strength of the terminal groups grows and upon transition to higher vinylogs. The new KCYs are efficient fluorophores, with fluorescence quantum yields up to 40% and large Stokes shifts, while their fluorescence bands extend into the near‐infrared (NIR) spectral region. Furthermore, the asymmetric KCYs are successfully transformed into the cationic polymethine‐styryl derivatives, which exhibit absorption and fluorescence spectra that are significantly shifted toward longer wavelengths.

## Graphical abstract

This work is dedicated to the synthesis and investigation of the structure, spectral and fluorescent properties of new asymmetric long‐chain ketocyanine dyes. It has been shown also that these compounds can serve as convenient precursors for the synthesis of highly asymmetric polymethine dyes, which exhibit large Stokes shifts and comparatively bright fluorescence in the region above 900 nm.

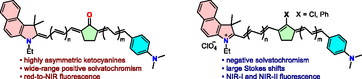

## 1 Introduction

**Scheme 1:**
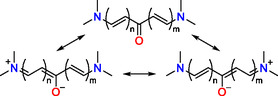
Major valence structures of a generic KCY. Possible substituent variations and ring structures are omitted for clarity.

Ketocyanines (KCYs) represent a captivating and multifaceted subclass of polymethine dyes. They can be considered either as bis(merocyanines) featuring a shared central acceptor carbonyl group or as cyanines with a carbonyl group integrated into the polymethine chain.^[^ ^1^ ^]^ The latter aspect is more obvious from the zwitterionic resonance structures, where the carbonyl group takes on a negative charge (**Scheme** **1**). Additionally, KCY dyes are structurally related and can be regarded as constituent elements of other bis‐dipolar polymethines,^[^ ^2^ ^]^ including squaraines and croconaines.^[^ ^3^, ^4^, ^5^ ^]^ However, the latter approach is not universally recognized, considering that in typical KCYs, the oxygen atom is at an even position of the polymethine backbone, counting from the terminal heteroatom, whereas in squaraines/croconaines, it is joined to an odd position (typically, in both cases it is the meso‐position, though not necessarily so). KCY dyes with an odd‐positioned oxygen exhibit significantly smaller excitation energy gaps at the same chromophore length but they remain an alluring rarity.^[^ ^6^ ^]^ Notably, they cannot be represented by a noncharged Lewis structure, and the quantum chemical calculations indicate their biradicaloid nature.^[^ ^3^ ^]^ From this point, we will only discuss KCYs with an even‐positioned oxygen.

**Scheme 2:**
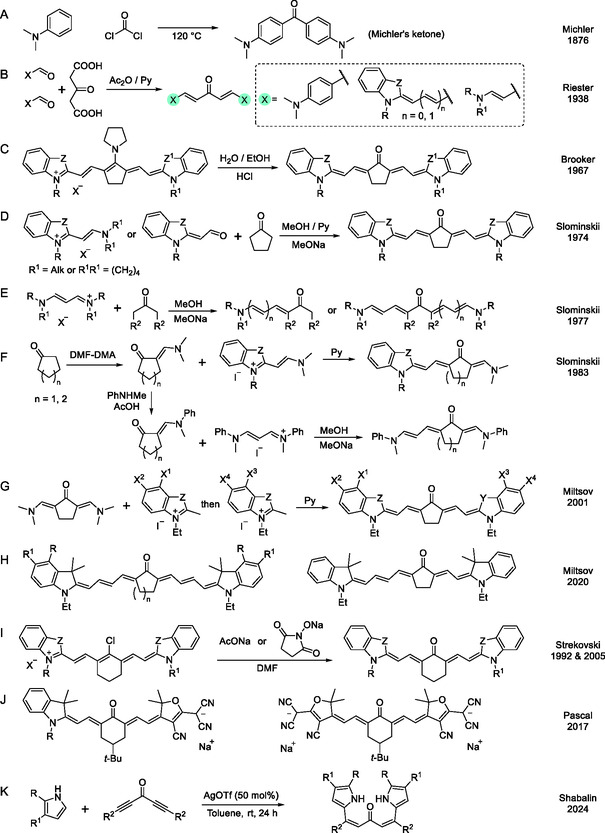
History of principal synthetic approaches toward KCYs and some notable specimens of dyes of this class.

To date, no comprehensive review of KCY dyes exists, necessitating a brief historical background. Michler's ketone (**Scheme** **2**A), a well‐known photosensitizer and a precursor for triarylmethane dyes, can be regarded as the oldest (1876) known KCY.^[^ ^7^ ^]^ However, Michler's ketone and other dyes of a similar structure are seldom considered as proper KCYs,^[^ ^8^ ^]^ with a colorful paper by Poe et al. being a rare exception.^[^ ^9^ ^]^ Hence, the living history of KCYs can be roughly traced back to a patent by Oskar Riester, who extended the previously described reaction of acetonedicarboxylic acid with aromatic aldehydes, resulting in pale yellow dyes, to more electron‐donating 4‐(dialkylamino)benzaldehyde, heterocyclic aldehydes of varying chain length, and 3‐aminoacrolein derivatives (Scheme 2B).^[^ ^10^ ^]^ Thus, all the main structural motifs of KCYs were first described in this work, which, however, did not gain much attention in the following years, likely due to the low photostability of the obtained open‐chain chromophores. Another limitation, relevant to the present study, is that only symmetric dyes can be obtained in this way.

The more practical KCYs, featuring rigidizing ring groups in the polymethine chain, were first described in a patent by L. Brooker and A. Fumia in 1967 (priority date).^[^ ^11^ ^]^ The synthetic scheme involved hydrolysis of meso‐pyrrolidino‐tricarbocyanines (Scheme 2C), obtained from 1‐pyrrolidino‐1‐cyclopentene and various dimethine‐hemicyanines. While both symmetric and asymmetric KCY dyes were claimed, and seemingly achievable by the described method, only symmetric ones were given as examples. A more convenient approach, suggested by Y. Slominskii and I. Radchenko in 1974, entailed the condensation of dimethine‐hemicyanines with cyclopentanone or other sym‐ketones in pyridine–methanol in the presence of sodium methoxide (Scheme 2D).^[^ ^12^ ^]^ The rather harsh conditions precluded the possibility to halt the reaction midway, resulting in the exclusive formation of symmetric KCYs. Further milestone in the KCY chemistry was independently achieved by Z. Krasnaya et al.^[^ ^13^ ^]^ and Y. Slominskii and I. Radchenko.^[^ ^14^ ^]^ The former devised a way toward mono‐ and bis[4‐(dimethylamino)butadienyl]ketones (with ring groups in the chain), while the latter employed a slightly different approach to produce mono‐ and bis[ω‐(alkyl(aryl)amino)polyenyl]ketones with varying chain length (Scheme 2E) and successfully derivatized some of the obtained KCYs into cationic species through the replacement of the meso‐oxygen with chlorine (using POCl_3_), hydrogen (LiAlH_4_), and phenyl (PhLi).^[^ ^14^ ^]^ Obviously, the mono‐products, attainable in excess ketone conditions, can serve as precursors for asymmetric KCYs. This was demonstrated by Y. Slominskii et al. in the following study, in which two types of asymmetric KCYs were prepared (Scheme 2F).^[^ ^15^ ^]^

Although bis[ω‐(alkyl(aryl)amino)polyenyl]ketones can be used as precursors for more extended polymethine chromophores, their ω‐dialkylamino analogs are much preferable in this regard. They were successfully utilized in the step‐by‐step strategy toward asymmetric KCYs devised by S. Miltsov et al. (Scheme 2G).^[^ ^16^ ^]^ Later, starting from *N*,*N*,*N*′,*N*′‐tetramethylvinamidinium salt, S. Miltsov et al. synthesized a number of longer‐chained KCYs, as well as a dye with noncentral position of the cyclopentanone ring in the chromophore (Scheme 2H).^[^ ^17^ ^]^

Among other studies on the preparation of KCYs with heterocyclic end groups, two papers by L. Strekovski et al. deserve mention.^[^ ^18^, ^19^ ^]^ Their approach is rather similar to the one by L. Brooker and A. Fumia.^[^ ^11^ ^]^ However, instead of meso‐pyrrolidino‐, much more readily available meso‐chloro‐tricarbocyanines with a five‐ or six‐membered ring in the polymethine chain were used as precursors. They were converted into respective KCYs in high yields by treatment with either sodium acetate or the sodium salt of *N*‐hydroxysuccinimide in dimethylformamide (DMF) (Scheme 2I). This method was later utilized by S. Pascal et al. to afford a number of near‐infrared (NIR) fluorescent dyes, including less‐typical anionic and dianionic KCYs with one or two acceptor end groups (Scheme 2J).^[^ ^20^ ^]^

Recently, D. Shabalin et al. suggested yet another synthetic approach to KCYs: a Lewis acid (AgOTf)‐catalyzed addition of pyrroles to penta‐1,4‐diyn‐3‐ones. (Scheme 2K).^[^ ^21^ ^]^ The obtained symmetric compounds are interesting both for being pyrrole‐based and for their *β,β*′‐disubstituted KCY structure. However, likely due to steric congestion, they exhibit comparatively low molar absorption, and the position of their long‐wavelength absorption band is only weakly dependent on the substituents in the pyrrole end groups and the polymethine chain.

Similar to Michler's ketone, certain KCYs, particularly bis(arylidene)cycloalkanones, were identified as effective singlet oxygen sensitizers.^[^ ^8^, ^22^ ^]^ However, deeper‐colored KCYs with heterocyclic end groups exhibit reduced intersystem crossing efficiency (likely due to a larger energy gap between their ^1^ππ* and ^1^nπ* states) and decreased photostability, making them less suitable for this application. Thus, the primary practical appeal of KCYs stems from their remarkable solvatochromism and solvatofluorochromism, which are notably well‐developed despite the internal near‐symmetry of the D–π–A–π–D′ chromophore, which results in smaller dipole moments when compared to typical merocyanines. Since a solitary carbonyl group is a relatively weak acceptor, the nonpolar valence structure (see Scheme 1) contributes more to the ground‐state electronic structure of known KCYs, which results in positive solvatochromism across the entire range of solvent polarity, irrespective of the donor strength(s) of the end groups. Within the KCY chromophore, the carbonyl oxygen is the most susceptible to strong specific solvation and other intermolecular interactions, which makes the electronic spectra more sensitive to highly electrophilic agents, such as protogenic solvents,^[^ ^23^, ^24^, ^25^ ^]^ acids,^[^ ^18^, ^19^, ^26^ ^]^ and metal ions.^[^ ^27^, ^28^, ^29^ ^]^ High environment responsiveness of absorption and fluorescence spectra of KCYs was assayed recently in bioimaging applications as well.^[^ ^30^ ^]^

The fluorescence quantum yields (FQYs) of KCYs are highly dependent on solvation, a phenomenon first observed by V. Danilov et al.^[^ ^31^ ^]^ To explain a sharp increase in the FQY of the studied dye in alcohols, they assumed that the formation of H‐bonded complexes between the carbonyl oxygen of the KCY and two molecules of a protogenic solvent heightens the isomerization barrier. Indeed, photoisomerization was early recognized as an important path of the excited‐state decay in KCYs.^[^ ^32^, ^33^, ^34^, ^35^ ^]^ Later, time‐resolved (transient) spectroscopy and quantum‐chemical studies suggested that the relative position of the ^1^ππ* and ^1^nπ* states, as well as their variation upon solvation, is another major factor governing the fluorescence decay rate of KCYs.^[^ ^36^, ^37^, ^38^ ^]^ Moreover, it was found that for some KCYs, the excited‐state lifetime can actually decrease in protogenic media, and this trend was not reversed even by introducing bridge groups restricting the possible formation of twisted intramolecular charge transfer states involving terminal dialkylamino donors.^[^ ^39^ ^]^ The authors proposed that the main reason for the observed behavior, in accordance with the classical energy gap law, is the enhanced internal conversion for positive solvatochromic dyes in polar solvents. Their assumption, while most reasonable for deeper‐colored, especially NIR‐emitting, fluorophores, appears flawed in the case where the fluorescence maximum does not exceed 666 nm (in methanol) for the deepest‐colored julolidine derivative.^[^ ^39^ ^]^ A more plausible explanation lies in the enhancement of internal conversion in high‐polarity media due to inhomogeneous solvation. This hypothesis is supported by the observation that the long‐wavelength absorption band of KCYs, despite their positive solvatochromism, typically broadens in protogenic solvents.^[^ ^24^, ^38^ ^]^

The exploration of the non‐linear optical (NLO) properties of KCY chromophores has been less extensive compared to related merocyanines. This disparity can be attributed, in part, to the comparatively small first hyperpolarizability (*β*) of more accessible symmetric KCYs. Then, in the case of asymmetric ones, theoretical evaluation of the NLO properties becomes too intricate, depending on both the electronic structure of a molecule and its V‐shape profile, which varies with factors such as the size of the central ring (when present), the presence of aromatic rings in the chromophore, and potential isomerization.^[^ ^34^, ^35^, ^40^, ^41^ ^]^

Asymmetric KCYs are not uncommon. However, the regularities governing their spectral‐fluorescence properties have not been explored in detail. The most abundant group here is bis(arylidene)cycloalkanones and bis(arylidene)acetones,^[^ ^42^, ^43^, ^44^ ^]^ for which the only variable is the donor strengths of the end groups, and it has not much room for changing, since even the julolidine moiety, the most electron‐donating among end groups found in asymmetric KCYs, is considered a weak donor in the polymethine systems.^[^ ^45^ ^]^ There are a few examples of 2‐arylidene‐5‐cinnamylidenecyclopentanones, with different π‐chain lengths on either side of the carbonyl acceptor,^[^ ^46^, ^47^ ^]^ but, first, their spectral and fluorescent properties were studied very superficially, and second, the issue of only weak donors present remains. Several 2‐arylidene‐5‐[(dimethylamino)methylene]cyclopentanones were synthesized recently by S. Batalin.^[^ ^48^ ^]^ Structurally, they are similar to 2‐[2‐(hetarylidene)ethylidene]‐5‐[(dimethylamino)methylene]cyclopentanones by Y. Slominskii et al. (see Scheme 2F).^[^ ^15^ ^]^ In our view, such compounds should be considered as precursors to extended and deeper‐colored KCYs rather than as proper functional dyes, particularly given their less appealing spectral characteristics.^[^ ^15^, ^49^ ^]^ Conjugated bis(ω‐aminopolyenyl)ketones with ‘arms’ of varying length and geometry were often used for studying chromophore interactions in asymmetric KCYs.^[^ ^34^, ^40^ ^]^ While good testing objects, they lack the potential for variations in the donor strength of end groups. Additionally, shifting their absorption/fluorescence into the NIR spectral range, which holds considerable practical appeal, requires a sizable extension of the conjugation chain, which poses challenges in terms of availability and photodurability. Considering both the structure–properties relationships studies and possible practical applications, better results were obtained by S. Miltsov et al.^[^ ^16^, ^25^ ^]^ who synthesized and studied solvatochromism, pH sensitivity, and photostability (in ethanol solutions) of a series of indole‐, benzo[*e*]indole‐, and benzothiazole‐based KCYs. Still, in their earlier works, there were only dyes with the meso‐positioned carbonyl acceptor, the same π‐chain length, and minor differences in the donor strengths of the end groups, reflected in the close to zero Brooker's deviations of the absorption maxima. In their more recent paper,^[^ ^17^ ^]^ the only KCY with the noncentral carbonyl has the identical (indole) donor end groups. In sum, there have been so far no studies involving the synthesis and investigation of the spectral and fluorescence properties of asymmetric KCYs, for which both the donor strength of the end groups and the polymethines chain length varied.

## 2 Results and Discussion

### 2.1 Synthesis of Asymmetric KCYs

**Scheme 3:**
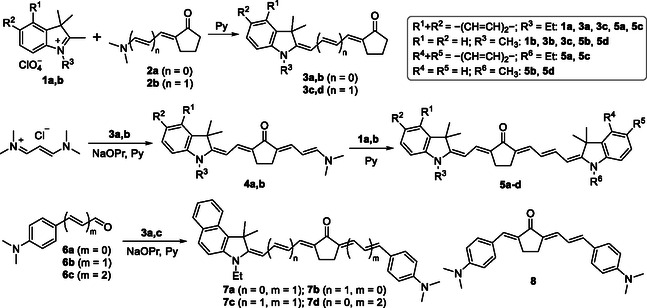
Synthesis of asymmetric KCY dyes.

Given the aim of synthesizing KCYs whose asymmetry arises from both the noncentral position of the acceptor fragment and differences in the structure (donor strength) of the end groups, we chose a synthetic path distinct from all those mentioned above. The key intermediates in this approach are merocyanines **3a–d** with a cyclopentanone terminal group (**Scheme** **3**). The derivatives with benzo[*e*]indole and indole end groups, which have similar electron‐donating properties, were obtained in preparative yields of 30%–40% (after column chromatography) from the respective heterocyclic salts **1a,b** and readily available enaminoketones **2a** ^[^ ^15^ ^]^ and **2b** ^[^ ^50^ ^]^ by refluxing in pyridine.

The reaction of merocyanines **3a,b** with *N*,*N*,*N*′,*N*′‐tetramethylvinamidinium chloride in pyridine with the addition of 1.5 M sodium propoxide affords 2‐heterarylidene‐5‐(3‐dimethylamino)propenylidene‐substituted cyclopentanones **4a,b** in high yields. Condensation of compounds **4a,b** with salts **1a,b** in pyridine yielded KCYs **5a–d**. All these dyes are asymmetric in the sense that the cyclopentanone group is positioned noncentrally within the polymethine chain. In the case of compounds **5c,d**, the donor strength of the terminal heterocyclic groups also differs, though just slightly.^[^ ^45^ ^]^ To achieve greater electronic asymmetry of the KCY chromophore, dyes **7a–d** were obtained by condensing merocyanines **3a,c** with 4‐(dimethylamino)benzaldehyde and its vinylogs **6a–c** in pyridine in the presence of sodium propoxide (Scheme 3). The “parent” dye **8**, required for analyzing the effect of terminal group donor strength on the spectral properties of dyes **7**, was synthesized using a previously reported method.^[^ ^22^ ^]^

**Scheme 4:**
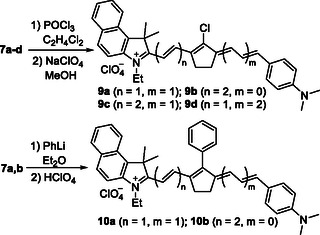
Chemical transformations of asymmetric KCYs.

It is known that KCYs can serve as precursors for the synthesis of cationic polymethine dyes. To date, the reactivity of the carbonyl group has been studied only in symmetric KCYs with heterocyclic end groups, from which the corresponding symmetric tricarbocyanines—either unsubstituted or meso‐substituted—have been synthesized.^[^ ^14^ ^]^ Notably, such meso‐substituted cyanines can be readily obtained through alternative synthetic pathways. In contrast, less accessible asymmetric KCYs have not been explored in this regard. At that, KCY dyes with a noncentral carbonyl group provide access to much more exotic cationic derivatives. Among the synthesized in this work KCYs, dyes **7a–d** exhibit the highest electronic asymmetry. Therefore, they were selected for derivatization into the corresponding chloro‐substituted polymethines **9a–d** by treatment with POCl_3_ in 1,2‐dichloroethane, followed by anion exchange to perchlorate. This exchange was performed to leverage the lower solubility of perchlorate salts, which facilitated the isolation and purification of the desired dyes. (**Scheme** **4**). The interaction of compounds **7a,b** with phenyllithium in diethyl ether, followed by treatment with perchloric acid, led to another pair of isomeric asymmetric cyanines, **10a,b**, comprising a phenyl substituent in the polymethine chain.

### 2.2 Structure of the New Compounds. Quantum Chemical Calculations

The structure of the synthesized compounds was confirmed using high‐resolution mass spectrometry (HR‐MS), ^1^H, and ^13^C NMR spectral data. Analysis of the ^1^H NMR spectra also allowed us to determine reliably the dyes’ predominant conformation, even without employing techniques that enable the study of through‐space interactions, such as NOESY.

**Figure 1:**
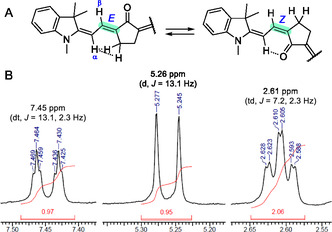
A) Possible conformations of the studied chromophores near the cyclopentanone ring. B) Snippets of the ^1^H NMR spectrum of merocyanine **3b** in (CD_3_)_2_SO, showing strong long‐range spin–spin coupling between the *β* H‐atom of the polymethine chain and the adjacent cyclopentanone CH_2_ group.

Literature data indicate that for most polymethine dyes—cationic, anionic, and neutral (merocyanines)—an all‐*trans* conformation of the chromophore is typical in the absence of bulky substituents in the polymethine chain.^[^ ^1^, ^51^, ^52^ ^]^ This rule generally holds for derivatives with five‐ and six‐membered rings in the polymethine chain, provided they do not introduce significant steric congestion that could induce trans–cis conformational changes. Indeed, the ^1^H NMR spectral data for the synthesized KCYs indicate a trans conformation for all ‘open’ segments of the chromophore, as evidenced by vicinal spin–spin coupling constants in the range of 12.0–14.1 Hz. At the same time, these KCYs comprise an electron‐accepting carbonyl group, which could hypothetically stabilize *Z*‐conformation (cis) of the adjacent to the ring C=C bonds via the weak C—H···O=C interaction (**Figure** **1**A). Similar stabilization has been reported, for instance, in dimethine merocyanines with the rhodanine acceptor group, as well as in the merocyanine form of spiropyrans.^[^ ^51^, ^53^, ^54^ ^]^ Analysis of the ^1^H NMR spectra of KCYs **5a–d**, **7a–d**, and merocyanines **3a–d** indicates that *E*‐conformers (trans) predominate in all cases. This is primarily reflected in the significant differences in chemical shifts between the corresponding α and *β* (or γ and δ in the case of tetramethine derivatives) H‐atoms, reaching up to 2.2 ppm. In *Z*‐conformers, these differences would be much smaller, as the anisotropic effect of the carbonyl group would induce an additional downfield shift of the α (or γ) signal. Further supporting this conclusion is the strong spin–spin coupling between the *β*‐ (or δ‐) H‐atoms of the polymethine chain and the adjacent cyclopentanone CH_2_ group (Figure 1B). This long‐range ^4^ *J*_HH_ spin–spin interaction is conformationally dependent,^[^ ^55^ ^]^ and only in the *E*‐isomer does the corresponding H—C—C—C—H fragment adopt a favorable W‐like geometry.

For the most electronically asymmetric KCYs **7a‐d** and corresponding cationic styryls **9a–d**, quantum chemical calculations were performed using the density functional theory (DFT)‐B3LYP/6‐31G(d,p) method. The solvent effect was accounted for using the integral equation formalism polarizable continuum model (PCM)^[^ ^56^ ^]^ with dichloromethane (DCM) as the model solvent. To reduce computation time, the *N*‐ethyl group in the benzo[*e*]indole moiety was replaced with a methyl group. Considering both literature data and the experimental results presented above, the all‐trans conformation of the polymethine chain was chosen as the initial structure. An additional calculation was performed for dye **7b** for the isomer, in which the double bond between the cyclopentanone and 4‐(dimethylamino)benzylidene fragments has *Z*‐conformation, with a possible binding interaction between the carbonyl oxygen and the proximal H‐atom of the phenyl group. This isomer was found to be 15.8 kJ mol^−1^ less favorable than the all‐trans isomer (see ESI).

According to the calculations, the chromophores of dyes **7a–d** and **9a–d** are essentially planar in the ground state S_0_, although the dimethylamino group exhibits a slight pyramidal distortion in the former. A much greater difference is observed in bond length alternations (BLA)^[^ ^57^ ^]^ in their polymethine chains. In molecules **7a–d**, the BLA values calculated for the “open” polymethine fragment between the benzo[*e*]indole and cyclopentanone moieties range from 0.056 to 0.057 Å. The BLA values in the other ‘open’ polymethine fragment are much bigger and vary more significantly (0.077–0.093 Å), though the most extreme value of 0.093 Å observed in **7b** may be attributed to both the presence of only two C—C bonds in this case (preventing averaging) and some steric strain, which also results in a CCC angle of 131.7° at the corresponding methine group. For styryls **9a–d**, BLA in the first polymethine fragment is very small (|BLA| = 0.002–0.015 Å). It decreases in the second fragment as well (BLA = 0.035–0.058 Å) but remains significantly more pronounced. Overall, these data correlate with the lower electron‐donating ability of the 4‐(dimethylamino)phenyl end group and suggest an increase in the electronic symmetry of the chromophore when transitioning from KCYs to the corresponding cationic styryls.

While time‐dependent DFT (TD‐DFT) calculations are known to systematically overestimate the energy of the longest‐wavelength electronic transition in various polymethine dyes,^[^ ^58^ ^]^ they show much better agreement with experimental data for KCYs.^[^ ^59^ ^]^ This is presumably due to the lower contribution of the polymethine charge resonance compared to more electronically symmetric dyes and the weaker charge transfer relative to dipolar merocyanines. Nevertheless, we performed these calculations primarily to explore the nature of the long‐wavelength electronic transitions in our dyes, as low‐lying ^1^nπ* states in KCYs can significantly influence their photophysical properties. Additionally, we aimed to assess whether quantum chemical methods could accurately predict the relative positions of spectral bands in isomers with different cyclopentanone unit locations.

**Figure 2:**
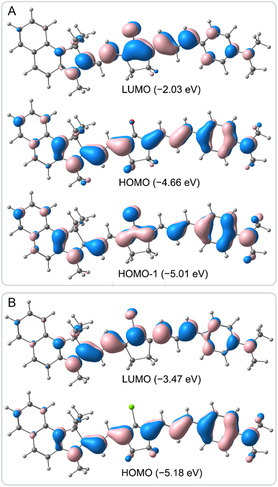
Molecular orbitals of dyes **7a** (A) and **9a** (B) (PCM_CH2Cl2_/DFT‐B3LYP/6‐31G(d,p), contour value is 0.03 bohr^−3/2^).

It was found that, in the PCM_DCM_/DFT‐B3LYP/6‐31G(d,p) calculations, the ^1^nπ* transitions in all four molecules **7a–d** have relatively high energies and correspond only to the third excited state (S_3_ ← S_0_). The most long wavelength and intense ^1^ππ* transition, S_1_ ← S_0_, is almost exclusively associated with the highest ocupied molecular orbital (HOMO) → lowest unoccupied molecular orbital (LUMO) excitation and is accompanied by electron density transfer from the terminal groups to the polymethine chain and the oxygen atom of the carbonyl group (**Figure** **2**). Interestingly, the calculated transition energies (maxima) for **7a–d** are rather close to the experimental values and correctly predict the relative positions of absorption maxima in pairs of isomers **7a**–**7b** and **7c**–**7d** (Table S2, Supporting Information). On the other hand, the TD‐DFT calculations overestimate vinylene shifts here.

The HOMOs of dyes **7a**–**d** are both in topology and energy very similar to those of the corresponding styryls **9a**–**d** (Figure 2). Thus, the bathochromic shift of the absorption bands when transitioning from KCYs to styryls must primarily be due to differences in their LUMOs. A distinctive feature of KCYs, arising from the bis nature of their chromophore, is the presence of a low‐lying S_2_ ← S_0_ transition (HOMO–1 → LUMO). However, this transition is significantly less intense than S_1_ ← S_0_, which can be attributed to the nearly 180° angle between the two “mono” chromophores in these KCYs.

### 2.3 UV–Vis Spectral Properties. Solvatochromism

Analysis of electronic absorption and fluorescence spectra of organic dyes in solvents of varying polarity (solvatochromism) is the primary method for studying not only their spectral properties but also their electronic structure.^[^ ^60^ ^]^ This is particularly relevant for donor–acceptor chromophores, where solvation effects have the most significant impact on electronic structure, and KCYs belong to this wide class of organic dyes. A prerequisite for reliable conclusions based on solvatochromism is a sufficiently wide range of solvent polarity. Furthermore, solvents must be structurally diverse, as, for example, polar protogenic and nonprotogenic ones may solvate the studied chromophore differently. For this work, we chose *n*‐hexane, toluene, ethyl acetate, DCM, DMF, and ethanol.

However, it was found that the long‐chained KCYs are not sufficiently stable in toluene and DCM solutions, likely due to effective singlet oxygen sensitization. This issue was partially solved by distilling spectral‐grade toluene under an argon flow to remove oxygen. In the case of DCM, the absorption spectra showed partial decomposition of some compounds even in freshly distilled spectral‐grade solvent. Therefore, the corresponding data were discarded from further analysis, except for the fluorescence spectra of a number of KCYs, which were measured in DCM as well and were free from any signs of impurities.

For styryl dyes **9a–d** and **10a,b**, the choice of solvents is limited by both their lower solubility in low‐polarity media and the tendency of ionic polymethines, especially long‐chain ones, to form tight ion pairs, which significantly alters their spectral and fluorescence properties.^[^ ^61^, ^62^ ^]^ Additionally, compounds **9a–d** proved to be unstable in DMF, likely due to their reactivity toward nucleophilic substitution of the chlorine atom in the polymethine chain.

**Figure 3:**
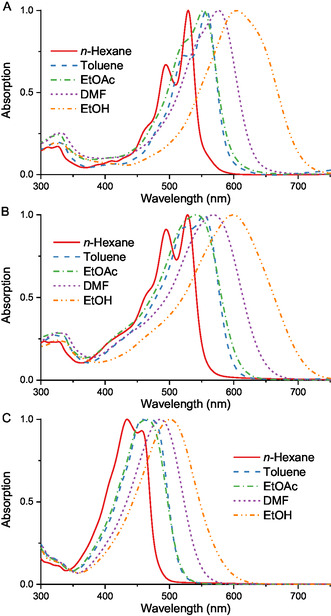
Normalized UV–vis spectra of dyes **5a** (A), **7c** (B), and **8** (C) in solvents of varying polarity.

All synthesized asymmetric KCYs, regardless of the electron‐donating strength of their end groups and the polymethine chain length, exhibit distinct positive solvatochromism, with the maximum redshift observed in the polar protogenic ethanol (**Table** **1**, **Figure** **3**). An exception to this trend is the spectral behavior in the toluene–ethyl acetate solvent pair. While ethyl acetate is a slightly more polar medium in terms of both dielectric constant and microscopic polarity (Table S1, Supporting Information), the transition from toluene to ethyl acetate results in a small (3–7 nm) hypsochromic shift of the absorption maxima for the studied KCYs, except for dye **8**, which has two weakly electron‐donating 4‐dimethylaminophenyl termini. This is likely due to the significantly higher refractive index (*n*_D_) of toluene, an increase in which results in a bathochromic shift of spectral bands regardless of the sign of the dye's solvatochromism.^[^ ^63^ ^]^

**Table 1:** Characteristics of the long‐wavelength absorption bands of the studied KCYs.

Dye	Solvent	*λ* ^a^_max_ (nm)	*ε*×10^−3^ (m^2 ^mol^−1^)
**5a**	*n*‐Hexane	529, 494	13.6, 9.31
	Toluene	557	8.05
	EtOAc	553	9.01
	DMF	576	10.3
	Ethanol	604	8.83
**5b**	*n*‐Hexane	507, 475	12.4, 9.14
	Toluene	534	9.90
	EtOAc	529	9.75
	DMF	551	8.45
	Ethanol	576	9.46
**8**	*n*‐Hexane	457. 434	5.28. 5.67
	Toluene	458	5.98
	EtOAc	464	6.53
	DMF	486	6.50
	Ethanol	502	6.49
**5c**	*n*‐Hexane	518, 485	14.6, 9.15
	Toluene	546	11.3
	EtOAc	541	11.7
	DMF	564	7.43
	Ethanol	590	10.5
**5d**	*n*‐Hexane	521, 494	13.2, 7.06
	Toluene	548	10.6
	EtOAc	542	11.6
	DMF	565	8.55
	Ethanol	593	11.0
**7a**	*n*‐Hexane	503, 472	8.58, 6.92
	Toluene	524	7.39
	EtOAc	518	6.16
	DMF	537	5.69
	Ethanol	560	7.95
**7b**	*n*‐Hexane	512, 484	9.38, 8.00
	Toluene	534	8.23
	EtOAc	526	7.82
	DMF	550	7.53
	Ethanol	576	8.09
**7c**	*n*‐Hexane	528, 495	8.48, 7.73
	Toluene	554	7.08
	EtOAc	543	6.88
	DMF	568	6.59
	Ethanol	599	7.05
**7d**	*n*‐Hexane	518, 488	–^a)^
	Toluene	536, 516	7.01, 7.01
	EtOAc	520	6.88
	DMF	546	7.04
	Ethanol	573	5.87

^a)^Insufficient solubility. Possible aggregation.

A difference in the shape of the absorption bands in solvents of varying polarity is noteworthy (Figure 3). In the least polar *n*‐hexane and to a lesser extent in toluene, a vibrational structure typical of both carotenoids and polymethine dyes is observed, with spacing between vibronic maxima ranging from 1050 to 1340 cm^−1^.^[^ ^64^, ^65^ ^]^ With the exception of the least dipolar chromophore **8**, the longest‐wavelength vibronic maximum is the most intense one even in *n*‐hexane (Table 1). In more polar media, especially in highly polar DMF and ethanol, the absorption bands broaden and become bell‐shaped (Figure 3). A similar change in band shape with solvent polarity was also observed for symmetric KCYs.^[^ ^38^ ^]^

This tendency is rather unusual, as a large proportion of positively solvatochromic donor–acceptor dyes, such as merocyanines, exhibit band narrowing and develop the characteristic “cyanine‐like” contour with increasing solvent polarity.^[^ ^1^ ^]^ A possible explanation is strong inhomogeneous solvation of KCY molecules in polar media, as ^1^H NMR data and the similarity of fluorescence excitation and absorption spectra indicate the absence of a significant fraction of conformers in solvents of varying polarity.

The dependence of the molar absorption coefficient (*ε*) of the obtained long‐chain KCYs on solvent polarity is irregular (Table 1). In most cases, the maximum *ε* value is observed in *n*‐hexane, which correlates with the narrowing of the absorption bands in this solvent (Figure 3). The minimum *ε* values for most of the studied dyes are recorded in DMF, yet no new absorption bands attributable to possible decomposition products were observed in this solvent. Among the KCYs with identical end groups (**5a**, **5b**, **8**), the deepest and most intense absorption bands are observed for **5a**, which has the most electron‐donating benzo[*e*]indole termini. The parameters of its indole‐derived analog **5b** are very close.

The UV–vis spectral properties of KCYs **5c**, **5d** are intermediate between those of the corresponding “parent” dyes **5a** and **5b** (Table 1). In this case, due to the asymmetry of the polymethine chain, with the cyclopentanone moiety in a noncentral position, classical Brooker's deviation of absorption maximum is not applicable. Still, the *λ* ^a^_max_ values of dyes **5c** and **5d** in each solvent are close to the arithmetic mean of the corresponding parameters of dyes **5a** and **5b**, as the electron‐donating abilities of benzo[*e*]indole and indole differ only slightly. The differences in the absorption maxima of **5c** and **5d** are very small, ranging from 1 to 3 nm depending on the solvent. In all cases, dye **5d**, in which the benzo[*e*]indole end group is connected to the cyclopentanone fragment via a longer tetramethine chain, exhibits a longer‐wavelength maximum.

The difference in electron‐donating abilities of the end groups is significantly greater in dyes **7a**–**d**. Accordingly, in the **7a**–**7b** pair, the position of the cyclopentanone fragment has a much stronger effect on the long wavelength absorption than in the **5c**–**5d** pair, while in both cases, the isomer with the longer polymethine segment adjacent to the benzo[*e*]indole residue exhibits a more bathochromic absorption maximum (Table 1). Notably, for KCYs **7a** and **7b**, the absorption maxima are noticeably, up to 15 nm, red‐shifted compared to the arithmetic mean values calculated from the spectra of the corresponding parent dyes **5a** and **8**. Hence, spectral deviation is indeed an inadequate parameter for assessing the electronic asymmetry of such KCYs. It is worth recalling that this parameter was introduced for typical single‐chromophore dyes,^[^ ^66^ ^]^ whereas KCYs, as previously noted, can be considered as bis(merocyanines).

Dyes **7c** and **7d** were synthesized to study the effect of further elongation of the polymethine chain on the spectral properties of “electronically asymmetric” KCYs. Among them, molecule **7c** is a vinylog of **7a**, with an additional vinylene group between the benzo[*e*]indole and cyclopentanone fragments, as well as a vinylog of **7b**, with an extended chain between the cyclopentanone and 4‐dimethylaminophenyl moieties. The transition to **7c** in both cases is accompanied by bathochromic shifts of the absorption band, minor compared to the typical vinylene shift of 100 nm observed in symmetric polymethines, along with an increase in the solvatochromic range; the latter expands even in terms of the energy scale, ν [cm^−1^]. The transition from **7a** to its vinylog **7d**, which contains five methine groups between the cyclopentanone and 4‐dimethylaminophenyl fragments, results in significantly smaller bathochromic shifts of the absorption band than in the case of **7c**. In ethyl acetate, the vinylene shift is equal to merely 2 nm, due to a redistribution of band intensity toward higher vibronic transitions. Notably, the solvatochromic range of dye **7d** turned out to be smaller than that of its shorter vinylog **7a** (Table 1).

### 2.4 Fluorescence Spectra

**Figure 4:**
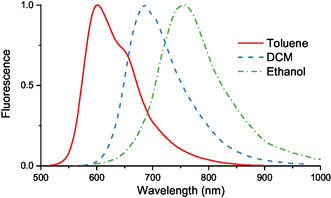
Normalized fluorescence spectra of dye **7c** in solvents of varying polarity.

The fluorescent properties were studied for more electron‐asymmetric KCYs **7a‐d**. These dyes exhibit positive solvatofluorochromism (**Table** **2**, **Figure** **4**), with solvatofluorochromic shifts significantly exceeding solvatochromic shifts, reaching 199 nm (4420 cm^−1^) in the toluene–ethanol solvent pair for compound **7d** [for comparison, the solvatochromic shift in this solvent pair is 37 nm (1200 cm^−1^)]. Accordingly, Stokes shifts increase with the solvent polarity (Table 2). They also grow with the extension of the polymethine chain, as seen in the transition from **7a,b** to **7c,d**. In ethanol, the fluorescence maxima of the latter two dyes fall within the practically important NIR spectral region.

**Table 2:** Spectral and fluorescence characteristics of KCYs 7a–d and the derived cationic cyanines 9a–d and 10a,b.

Dye	Solvent	*λ* ^a^_max_ (nm)	*λ* ^f^_max_ (nm)	*φ*_f_ (%)	Δ*ν*_S_ (cm^−1^)
**7a**	Toluene	524	555	37	1070
	DMF	537	614	22	2340
	Ethanol	560	684	19	3240
**7b**	Toluene	534	576	5.9	1370
	DMF	550	660	41	3030
	Ethanol	576	711	29	3300
**7c**	Toluene	554	601	16	1410
	DCM	570	686	27	2970
	Ethanol	599	756	8.1	3470
**7d**	Toluene	536	579	25	1390
	DCM	546	653	12	3000
	Ethanol	573	778	4.1	4600
**9a**	DCM	788	989	3.3	2580
	Ethanol	712	990	0.92	3940
**9b**	DCM	764	987	2.8	2960
	Ethanol	668	974	0.74	4700
**9c**	DCM	751	–	–	–
	Ethanol	686	–	–	–
**9d**	DCM	802	–	–	–
	Ethanol	718	–	–	–
**10a**	DCM	782	965	6.0	2430
**10b**	DCM	769	963	5.2	2620

When comparing the shape of the absorption and fluorescence bands of dyes **7a**–**d** (cf. Figure 3B and 4), a breakdown of their mirror symmetry becomes evident due to the relative narrowing of the latter, at least in polar solvents. This narrowing becomes even more apparent on an energy scale. For instance, in compound **7c**, the full widths at half maximum of the long‐wavelength absorption and fluorescence bands in ethanol are 2590 and 2060 cm^−1^, respectively. The fluorescence band shapes of the studied KCYs are more similar to the universal contour typical of electron‐symmetric polymethines. This suggests that in the fluorescent state S_1_, the electronic structure of their chromophores is more “electron‐symmetric” than in the ground state S_0_. The increase in vinylene shifts for fluorescence bands compared to absorption bands supports this conclusion. This increase is observed even in the less polar toluene and reaches its maximum in ethanol, where, for the **7a**–**7d** vinylogous pair, the vinylene shift *λ* ^f^_max_ reaches 94 nm, only slightly lower than the typical 100 nm value of symmetric polymethines.

All four KCYs **7a**–**d** are relatively bright fluorophores. Their FQYs (*φ*_f_) exhibit an irregular dependence on solvent polarity, with different trends observed for different compounds (Table 2). However, a consistent pattern is the decrease in FQY with the extension of the polymethine chain, which is particularly pronounced in ethanol. Presumably, this decrease is driven by both the enhancement of internal conversion in deeper fluorophores and increased solvation, as higher vinylogs possess more solvation centers and greater overall molecular polarizability.

### 2.5 Cationic Dyes Derived from Asymmetric KCYs

Cationic styryls **9a–d** absorb and emit light in a significantly longer‐wavelength region compared to the corresponding KCYs **7a–d** (Table 2). Notably, the spectral effects of this structural modification are nonuniform. The most pronounced spectral shift occurs in dye pairs that comprise more methine groups in the polymethine chain between the cyclopentanone/cyclopentene and 4‐dimethylaminophenyl fragments (**7a** → **9a**, **7d** → **9d**). The difference is so substantial that dye **9a** exhibits a longer‐wavelength absorption maximum than its higher vinylog **9c** (Table 2). We cannot offer an explanation for this phenomenon. A priori, we even assumed that the isomeric styryls would exhibit more uniform spectral properties than their respective parent KCYs. Then this assumption was contradicted by experimental results, at least for the absorption spectra.

Dyes **9a,b** exhibit pronounced negative solvatochromism in the DCM–ethanol solvent pair; in both solvents, the absorption bands are broad and bell‐shaped, characteristic of cyanines with end groups of markedly different electron‐donating abilities.^[^ ^64^ ^]^ Unlike the parent KCYs, the solvatofluorochromic range of **9a,b** is significantly smaller than their solvatochromic range, which is typical of asymmetric cationic polymethines.^[^ ^67^ ^]^ Also, the differences in *λ* ^f^_max_ between the isomers **9a** and **9b** are much smaller than the differences in *λ* ^a^_max_ in both solvents (Table 2).

The FQYs of styryls **9a,b** in less polar DCM are relatively high, considering that their fluorescence maxima lie well beyond 900 nm (Table 2), a spectral region where bright organic fluorophores (with *φ*_f_ > 10%) are practically impossible due to enhanced internal conversion. Increasing the solvent polarity by switching to ethanol leads to a several‐fold decrease in FQYs, likely due to stronger solvation of their molecules and greater electronic asymmetry in the ground state, both of which enhance internal conversion.

Unfortunately, due to instrumental limitations, the fluorescence of dyes **9c** and **9d**, including the expected emission maxima, could not be recorded. Within the instrumental range, below 1070 nm, only the short‐wavelength slopes of their fluorescence bands were observed. Still, based on the existing knowledge on the spectral and fluorescence properties of asymmetric cyanines, it can be reasonably assumed that the vinylene shifts of the fluorescence maxima when going from **9a,b** to **9c,d** are close to or even exceed 100 nm.

**Figure 5:**
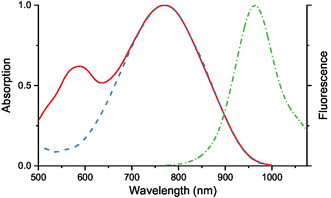
Normalized absorption and fluorescence (dash‐dot) spectra of dye **10b** in dichloromethane; for absorption, the solid line corresponds to *C* = 1.2 × 10^−5 ^M, while the dashed line was obtained after 25‐fold dilution.

Phenyl‐substituted styryls **10a,b** absorb within the same spectral range as their chloro‐substituted analogs **9a,b** (Table 2). Once again, the isomer with the longer polymethine chain between the cyclopentene and 4‐dimethylaminophenyl moieties (**10a**) exhibits a more bathochromic absorption, although the difference in *λ* ^a^_max_ is much smaller than in the **9a**–**9b** pair. It was also observed that dyes **10a,b** are more prone to aggregation than their analogs **9a,b**. In moderately polar DCM, an additional absorption band is observed in the spectrum, which disappears either upon dilution or upon addition of a more polar solvent (**Figure** **5**).

The fluorescence maxima of styryls **10a,b** are hypsochromically shifted relative to those of their chloro‐substituted analogs **9a,b**, resulting in smaller Stokes shifts. On the other hand, the FQYs of dyes **10a,b** are twice as high, making them more appealing for practical applications. An additional advantage is their greater chemical stability due to the absence of a halogen atom, susceptible to nucleophilic substitution.

## 3 Conclusion

Overall, the spectral and fluorescence properties of the synthesized long‐chain asymmetric KCYs were found to be rather similar to those of their symmetric vinylogs. At the same time, the spectral properties of the new dyes exhibit a dependence on the position of the cyclopentanone acceptor fragment, which becomes more pronounced as the electron‐donating abilities of the terminal groups diverge. The vinylene shifts of the absorption bands of the obtained KCYs are relatively small, limiting the possibility of developing such chromophores for absorption in the NIR region. However, their fluorescence bands in polar solvents, particularly in ethanol, exhibit large Stokes shifts as well as vinylene shifts significantly larger than those observed in the absorption spectra. So, their fluorescence emission in polar media lies in the red and NIR spectral regions. Additionally, their FQYs are relatively high, making these fluorophores attractive for biomedical applications, as they can be excited, for instance, by the second harmonic of a Nd:YAG laser (532 nm).

The cationic asymmetric styryl dyes derived from asymmetric long‐chain KCYs are of interest both from a practical perspective (large Stokes shifts, relatively bright fluorescence in the NIR region) and a synthetic standpoint. Specifically, the instability of compounds **9a‐d** in DMF solutions indicates their high reactivity in nucleophilic substitution reactions at the meso‐chlorine atom, which can be leveraged for further structural modifications and, consequently, tuning of their spectral properties.

## 4 Experimental Section

4.1

4.1.1

##### General Information

Reagents and solvents were purchased from commercial suppliers (Sigma‐Aldrich, Apollo Scientific, Fisher, Acros Organics, and Alfa Aesar) and were used in the synthetic part of the study without additional purification. Solvents for spectral measurements were purified according to standard laboratory procedures.^[^ ^68^ ^]^ Toluene was additionally distilled under an argon flow.

^1^H NMR spectra were recorded on a Bruker Avance III spectrometer (400 MHz), Varian VNMRS (500 and 600 MHz). The ^13^C{^1^H} NMR spectra were all recorded on a Bruker Avance III spectrometer (400 MHz). For cationic polymethines **9a–d** and **10a,b**, the ^13^C NMR spectra were not recorded due to solubility and aggregation issues. The ^13^C NMR chemical shifts are given to one decimal place, except in cases where such rounding would result in the apparent overlap of two or more distinct signals. The residual solvent peaks were used as internal standards: CDCl_3_ (*δ*_H _= 7.26 ppm, *δ*_C _= 77.16 ppm), (CD_3_)_2_SO (*δ*_H_ = 2.50 ppm), and CD_3_CN (*δ*_H_ = 1.94 ppm). Separation and mass spectrometric detection of samples were performed using an Infinity 1260 UHPLC system (Agilent Technologies, Waldbronn, Germany) coupled with a 6224 Accurate Mass TOF LC/MS system (Agilent Technologies, Singapore). Electronic absorption spectra were recorded in the 250–1000 nm (UV–vis‐NIR) range using a Cary 5000 spectrophotometer and 1‐cm cuvettes. Steady‐state fluorescence spectra were measured using a Solar CM‐2203 spectrofluorometer (modified to extend the measurement range to 1070 nm). To minimize the inner filter effect, the optical density of dye solutions in fluorescence measurements did not exceed 0.1 at the absorption maximum. The FQY (*φ*_f_) were determined relative to indodicarbocyanine iodide (*φ*_f_ = 0.25, EtOH) and indotricarbocyanine iodide (*φ*_f_ = 0.28, EtOH),^[^ ^67^ ^]^ and corrected for solvent refractive indices.

DFT/TD‐DFT calculations were performed with the Gaussian 09 program suite using the hybrid B3LYP functional and the double–ζ basis set 6‐31G(d,p).^[^ ^69^ ^]^ The ground‐state geometry optimizations were followed by frequency calculations to verify that the found stationary points were true minima. In the case of fast absorption processes (Franck–Condon principle), the nuclear relaxation was avoided by employing state‐specific solvation calculations in terms of fast electronic cloud reorganizations and slow solvent and solute nuclear motions.^[^ ^70^ ^]^

##### Experimental Procedures and Product Characterization

*Representative Synthetic Procedure for Merocyanines 3a–d*: Salt **1a** (9.5 g, 28 mmol) and 2‐[(dimethylamino)methylene] cyclopentanone **2a** (3.9 g, 28 mmol) were refluxed for 2 h in pyridine (30 mL). After cooling, the mixture was poured on ice (200 g) and left overnight. The aqueous layer was decanted, and the oily residue was dissolved in DCM, washed with water (2 × 150 mL), and dried over MgSO_4_. After filtration and solvent removal, the crude product was purified by chromatography on silica gel (hexane–ethyl acetate 3:1) to give **3a** as a dark‐yellow solid.

*2‐[2‐(3‐Ethyl‐1,1‐dimethyl‐1H‐benzo[e]indol‐2(3H)‐ylidene)ethylidene]cyclopentanone (**3a**)*: Yield 46%. ^1^H NMR (400 MHz, CDCl_3_) *δ*: 8.03 (1H, d, *J* = 8.7 Hz), 7.74–7.88 (3H, m), 7.49 (1H, t, *J* = 7.6 Hz), 7.30 (1H, t, *J* = 7.8 Hz), 7.10 (1H, d, *J* = 8.7 Hz), 5.30 (1H, d, *J* = 13.1 Hz), 3.87 (2H, q, *J* = 7.1 Hz), 2.71 (2H, t, *J* = 7.2 Hz), 2.41 (2H, t, *J* = 7.7 Hz), 1.91–2.06 (8H, m), 1.34 (3H, t, *J* = 7.1 Hz). ^13^C{^1^H} NMR (400 MHz, CDCl_3_) *δ*: 206.0, 165.8, 140.8, 130.2, 130.1, 129.9, 129.8, 129.7, 129.0, 127.1, 127.0, 122.9, 121.9, 109.2, 92.1, 48.8, 39.1, 37.4, 28.0 (C(CH_3_)_2_), 27.3, 20.0, 11.6. HRMS‐ESI [(M + H)^+^]: m/z calculated for C_23_H_25_NO: 332.2009; found: 332.2008. *λ* ^a^_max_ (MeOH) = 456 nm.

*2‐[2‐(1,3,3‐Trimethyl‐2‐indolinylidene)ethylidene]cyclopentanone (**3b**)*.^[^ ^15^ ^]^^1^H NMR (400 MHz, DMSO‐*d*_6_) *δ* 7.45 (1H, dt, *J* = 13.1, 2.3 Hz), 7.32 (1H, d, *J* = 7.3 Hz), 7.20 (1H, t, *J* = 7.6 Hz), 6.86–6.99 (2H, m), 5.26 (1H, d, *J* = 13.1 Hz), 3.23 (3H, s), 2.61 (2H, td, *J* = 7.2, 2.3 Hz), 2.22 (2H, t, *J* = 7.8 Hz), 1.87 (2H, quin, *J* = 7.5 Hz), 1.52 (6H, s). ^13^C{^1^H} NMR (400 MHz, CDCl_3_) *δ*: 206.1, 165.0, 144.4, 139.5, 129.6, 127.9, 127.7, 121.8, 121.1, 106.9, 93.0, 46.7, 39.1, 29.5, 28.7 (C(CH_3_)_2_), 27.2, 20.0.

*2‐[4‐(3‐Ethyl‐1,1‐dimethyl‐1H‐benzo[e]indol‐2(3H)‐ylidene)but‐2‐en‐1‐ylidene]cyclopentanone (**3c**)*: Dark green solid; yield 34%. ^1^H NMR (400 MHz, (CD_3_)_2_SO) *δ*: 8.03 (1H, d, *J* = 8.7 Hz), 7.80–7.91 (2H, m), 7.42–7.55 (2H, m), 7.22–7.36 (2H, m), 7.08 (1H, d, *J* = 12.0 Hz), 6.08 (1H, t, *J* = 12.9 Hz), 5.68 (1H, d, *J* = 12.3 Hz), 3.84 (2H, q, *J* = 7.1 Hz), 2.64 (2H, t, *J* = 7.0 Hz), 2.23 (2H, t, *J* = 7.3 Hz), 1.78–1.96 (8H, m), 1.17 (3H, t, *J* = 7.1 Hz). ^13^C{^1^H} NMR (400 MHz, CDCl_3_) *δ*: 207.3, 161.6, 141.2, 140.6, 134.5, 130.6, 130.0, 129.9, 129.7, 129.5, 129.1, 127.0, 122.6, 121.8, 120.0, 109.1, 96.2, 48.3, 39.0, 37.2, 27.52, 27.48, 20.1, 11.9. HRMS‐ESI [(M + H)^+^]: m/z calculated for C_25_H_27_NO: 358.2165; found: 358.2151. *λ* ^a^_max_ (MeOH) = 494 nm.

*2‐[4‐(1,3,3‐Trimethyl‐2‐indolinylidene)but‐2‐en‐1‐ylidene]cyclopentanone (**3d**)*: Dark red solid, yield 29%. ^1^H NMR (400 MHz, (CD_3_)_2_SO) *δ*: 7.36 (1H, t, *J* = 13.2 Hz), 7.27 (1H, d, *J* = 7.3 Hz), 7.16 (1H, t, *J* = 7.6 Hz), 7.06 (1H, dt, *J* = 12.0, 2.5 Hz), 6.80–6.91 (2H, m), 6.04 (1H, dd, *J* = 13.9, 12.0 Hz), 5.57 (1H, d, *J* = 12.4 Hz), 2.62 (2H, td, *J* = 7.1, 2.5 Hz), 2.22 (2H, t, *J* = 7.7 Hz), 1.86 (2H, quin, *J* = 7.4 Hz), 1.53 (6H, s). ^13^C{^1^H} NMR (400 MHz, CDCl_3_) *δ*: 207.4, 160.9, 144.7, 140.4, 139.2, 134.4, 127.9, 127.7, 121.8, 120.5, 120.4, 106.6, 96.9, 46.2, 38.9, 29.3, 28.4 (C(CH_3_)_2_), 27.4, 20.1. HRMS‐ESI [(M + H)^+^]: m/z calculated for C_20_H_23_NO: 294.1852; found: 294.1839. *λ* ^a^_max_ (MeOH) = 473 nm.

##### General Synthetic Procedure for Dyes 4a,b

Merocyanine **3a** or **3b** (5.5 mmol), *N*,*N*,*N*′,*N*′‐tetramethylvinamidinium chloride (0.98 g, 6 mmol), and freshly prepared 1.5 M sodium propoxide (4.5 mL) were stirred overnight in pyridine (6 mL) at room temperature. The mixture was poured into water (300 mL), filtered, and the crude product was purified by column chromatography using chloroform:methanol (95:5) as eluent and then hexane:ethyl acetate (3:1) for the next chromatography.

*2‐[3‐(Dimethylamino)allylidene]‐5‐[2‐(3‐ethyl‐1,1‐dimethyl‐1H‐benzo[e]indol‐2(3H)‐ylidene)ethylidene]cyclopentanone (**4a**)*: Dark brown solid; yield 76%. ^1^H NMR (400 MHz, CDCl_3_) *δ*: 8.04 (1H, d, *J* = 8.6 Hz), 7.73–7.84 (3H, m), 7.46 (1H, t, *J* = 7.7 Hz), 7.24–7.30 (1H, m), 7.20 (1H, d, *J* = 11.9 Hz), 7.06 (1H, d, *J* = 8.7 Hz), 6.74 (1H, d, *J* = 12.6 Hz), 5.37 (1H, d, *J* = 13.0 Hz), 5.03 (1H, t, *J* = 12.3 Hz), 3.83 (2H, q, *J* = 7.1 Hz), 2.92 (6H, s), 2.70–2.78 (2H, m), 2.63–2.69 (2H, m), 1.98 (6H, s), 1.31 (3H, t, *J* = 7.1 Hz). ^13^C{^1^H} NMR (400 MHz, CDCl_3_) *δ*: 192.6, 163.2, 150.6, 141.2, 134.2, 132.8, 130.0, 129.9, 129.8, 129.6, 129.5, 129.2, 127.5, 126.9, 122.6, 121.9, 109.1, 96.2, 92.8, 48.6, 40.8 (br, N(CH_3_)_2_), 37.2, 27.8 (C(CH_3_)_2_), 24.1, 23.9, 11.5. HRMS‐ESI [(M + H)^+^]: m/z calculated for C_28_H_33_N_2_O: 413.2587; found: 413.2581. *λ* ^a^_max_ (MeOH) = 556 nm.

*2‐[3‐(Dimethylamino)allylidene]‐5‐[2‐(1,3,3‐trimethyl‐2‐indolinylidene)ethylidene]cyclopentanone (**4b**)*: Dark brown solid; yield 73%. ^1^H NMR (400 MHz, (CD_3_)_2_SO) *δ* 7.36 (1H, d, *J* = 13.0 Hz), 7.29 (1H, d, *J* = 7.4 Hz), 7.17 (1H, t, *J* = 7.7 Hz), 7.05 (1H, d, *J* = 12.5 Hz), 6.96 (1H, d, *J* = 12.3 Hz), 6.83–6.91 (2H, m), 5.27 (1H, d, *J* = 12.9 Hz), 4.96 (1H, t, *J* = 12.2 Hz), 3.19 (3H, s), 2.90 (6H, s), 2.56–2.65 (2H, m), 2.51–2.56 (2H, m), 1.54 (6H, s). ^13^C{^1^H} NMR (400 MHz, CDCl_3_) *δ*: 192.7, 162.6, 150.7, 144.8, 139.6, 134.4, 133.4, 129.5, 127.8, 127.3, 121.8, 120.5, 106.5, 96.2, 93.5, 46.4, [40.9, 40.8 (N(CH_3_)_2_)], 29.4, 28.6 (C(CH_3_)_2_), 24.0, 23.9. HRMS‐ESI [(M + H)^+^]: m/z calculated for C_28_H_33_N_2_O: 349.2274; found: 349.2270. *λ* ^a^_max_ (MeOH) = 539 nm.

##### General Synthetic Procedure for Dyes 5a–d

Salt **1** (2.4 mmol) and dye **4** (1.6 mmol) were refluxed for 2 h in pyridine (2 mL). After cooling, the mixture was poured on ice (200 g) and left overnight. The crude product was filtered, dried, and purified by column chromatography with hexane:ethyl acetate (3:1) as eluent. After solvent removal, the residue was crystallized from methanol, filtered, and dried in air.

*2‐[4‐(3‐Ethyl‐1,1‐dimethyl‐1H‐benzo[e]indol‐2(3H)‐ylidene)but‐2‐en‐1‐ylidene]‐5‐[2‐(3‐ethyl‐1,1‐dimethyl‐1H‐benzo[e]indol‐2(3H)‐ylidene)ethylidene]cyclopentanone (**5a**)*: Dark blue solid, yield 26%. ^1^H NMR (400 MHz, CDCl_3_) *δ*: 8.00–8.11 (2H, m), 7.93 (1H, d, *J* = 13.7 Hz), 7.72–7.86 (4H, m), 7.44–7.53 (2H, m), 7.39 (1H, t, *J* = 13.3 Hz), 7.24–7.33 (3H, m), 7.02–7.14 (2H, m), 6.21 (1H, t, *J* = 13.0 Hz), 5.58 (1H, d, *J* = 12.3 Hz), 5.42 (1H, d, *J* = 13.0 Hz), 3.87 (2H, q, *J* = 7.1 Hz), 3.79 (2H, q, *J* = 7.3 Hz), 2.70–2.90 (4H, m), 2.00 (6H, s), 1.94 (6H, s), 1.25–1.39 (6H, m). ^13^C{^1^H} NMR (400 MHz, CDCl_3_) *δ*: 193.0, 164.7, 160.5, 141.4, 140.9, 138.1, 136.3, 132.8, 131.9, 130.3, 130.2, 129.90, 129.89, 129.87, 129.63, 129.60, 129.5, 129.4, 129.2, 129.1, 127.0, 126.9, 122.9, 122.4, 122.0, 121.8, 121.1, 109.2, 109.1, 96.6, 93.0, 48.9, 48.2, 37.4, 37.1, 27.9 (C(CH_3_)_2_), 27.5 (C(CH_3_)_2_), 24.1, 24.0, 11.9, 11.6. HRMS‐ESI [(M + H)^+^]: m/z calculated for C_43_H_45_N_2_O: 605.3526; found: 604.3416.

*2‐[4‐(1,3,3‐Trimethylindolin‐2‐ylidene)but‐2‐en‐1‐ylidene]‐5‐[2‐(1,3,3‐trimethylindolin‐2‐ylidene)ethylidene]cyclopentanone (**5b**)*: Dark brown solid, yield 21%. ^1^H NMR (500 MHz, CD_3_CN) *δ*: 7.54 (1H, dt, *J* = 13.0, 2.3 Hz), 7.17–7.24 (2H, m), 7.14 (1H, d, *J* = 7.5 Hz), 7.03–7.12 (2H, m), 6.96 (1H, dt, *J* = 12.1, 2.3 Hz), 6.81 (1H, t, *J* = 7.3 Hz), 6.72–6.78 (2H, m), 6.68 (1H, d, *J* = 7.9 Hz), 6.07 (1H, dd, *J* = 13.8, 12.1 Hz), 5.50 (1H, d, *J* = 12.2 Hz), 5.29 (1H, d, *J* = 13.0 Hz), 3.17 (3H, s), 3.10 (3H, s), 2.61–2.66 (2H, m), 2.55–2.60 (2H, m), 1.52 (6H, s), 1.51 (6H, s). ^13^C{^1^H} NMR (400 MHz, CDCl_3_) *δ*: 192.9, 164.1, 159.9, 144.9, 144.5, 139.8, 139.3, 138.1, 136.3, 132.9, 132.4, 129.5, 127.9, 121.9, 121.8, 121.5, 121.0, 120.3, 106.9, 106.6, 106.4, 97.4, 93.8, 46.8, 46.2, 29.5, 29.3, 28.7 (C(CH_3_)_2_), 28.4 (C(CH_3_)_2_), 24.01, 23.96 HRMS‐ESI [(M + H)^+^]: m/z calculated for C_33_H_37_N_2_O: 477.2900; found: 477.2882.

*2‐[2‐(3‐Ethyl‐1,1‐dimethyl‐1H‐benzo[e]indol‐2(3H)‐ylidene)ethylidene]‐5‐[4‐(1,3,3‐trimethylindolin‐2‐ylidene)but‐2‐en‐1‐ylidene]cyclopentanone (**5c**)*: Dark brown solid, yield 53%. ^1^H NMR (400 MHz, CDCl_3_) *δ*: 8.03 (1H, d, *J* = 8.8 Hz), 7.72–7.84 (3H, m), 7.47 (1H, t, *J* = 7.8 Hz), 7.38 (1H, t, *J* = 13.2 Hz), 7.18–7.30 (4H, m), 7.04 (1H, d, *J* = 8.7 Hz), 6.94 (1H, t, *J* = 7.7 Hz), 6.72 (1H, d, *J* = 7.7 Hz), 6.19 (1H, t, *J* = 13.0 Hz), 5.56 (1H, d, *J* = 12.3 Hz), 5.32 (1H, d, *J* = 13.0 Hz), 3.78 (2H, q, *J* = 7.4 Hz), 3.23 (3H, s), 2.70–2.84 (4H, m), 1.93 (6H, s), 1.68 (6H, s), 1.30 (3H, t, *J* = 7.2 Hz). ^13^C{^1^H} NMR (400 MHz, CDCl_3_) *δ*: 192.9, 164.0, 160.6, 144.5, 141.3, 139.7, 138.3, 136.0, 133.0, 132.5, 129.90, 129.89, 129.6, 129.4, 129.3, 129.2, 127.8, 126.9, 122.5, 121.9, 121.8, 121.1, 121.0, 109.1, 106.8, 96.6, 93.8, 48.3, 46.7, 37.1, 29.5, 28.7 (C(CH_3_)_2_), 27.5 (C(CH_3_)_2_), 24.0, 24.0, 11.9. HRMS‐ESI [(M + H)^+^]: m/z calculated for C_38_H_41_N_2_O: 541.3213; found: 541.3195.

*2‐[4‐(3‐Ethyl‐1,1‐dimethyl‐1H‐benzo[e]indol‐2(3H)‐ylidene)but‐2‐en‐1‐ylidene]‐5‐[2‐(1,3,3‐trimethylindolin‐2‐ylidene)ethylidene]cyclopentanone (**5d**)*: Dark green solid, yield 30%. ^1^H NMR (400 MHz, CDCl_3_) *δ*: 8.05 (1H, d, *J* = 8.6 Hz), 7.90 (1H, d, *J* = 13.6 Hz), 7.83 (1H, d, *J* = 8.0 Hz), 7.78 (1H, d, *J* = 8.5 Hz), 7.49 (1H, t, *J* = 7.5 Hz), 7.14–7.33 (5H, m), 7.09 (1H, d, *J* = 8.5 Hz), 6.89 (1H, t, *J* = 7.6 Hz), 6.65 (1H, d, *J* = 7.8 Hz), 6.20 (1H, t, *J* = 13.0 Hz), 5.51 (1H, d, *J* = 12.2 Hz), 5.40 (1H, d, *J* = 13.2 Hz), 3.86 (2H, q, *J* = 7.2 Hz), 3.17 (3H, s), 2.72–2.84 (4H, m), 1.99 (6H, s), 1.61 (6H, s), 1.34 (3H, t, *J* = 7.0 Hz). ^13^C{^1^H} NMR (400 MHz, CDCl_3_) *δ*: 193.0, 164.7, 159.7, 144.9, 140.9, 139.3, 137.8, 136.5, 132.5, 131.8, 130.3, 130.2, 129.9, 129.63, 129.55, 129.1, 127.9, 127.0, 122.8, 121.9, 121.8, 121.5, 120.2, 109.2, 106.4, 97.4, 92.9, 48.9, 46.1, 37.4, 29.3, 28.4 (C(CH_3_)_2_), 27.9 (C(CH_3_)_2_), 23.98, 23.97, 11.6. HRMS‐ESI [(M + H)^+^]: m/z calculated for C_38_H_41_N_2_O: 541.3213; found: 541.3193.

##### General Synthetic Procedure for Dyes 7a–d

Merocyanine **3** (3 mmol), aldehyde **6** (3 mmol), freshly prepared 1.5 M sodium propoxide (0.45 mL), and pyridine (4 mL) were stirred for 20 min at room temperature. The product was precipitated with methanol (5–7 mL), filtered, and washed with methanol (2 × 10 mL).

*2‐[3‐(4‐(Dimethylamino)phenyl)allylidene]‐5‐[2‐(3‐ethyl‐1,1‐dimethyl‐1H‐benzo[e]indol‐2(3H)‐ylidene)ethylidene]cyclopentanone (**7a**)*: Recrystallized by dissolving in DMF and precipitating with hot ethanol. Dark solid, yield 52%. ^1^H NMR (400 MHz, CDCl_3_) *δ*: 8.06 (1H, d, *J* = 8.7 Hz), 7.95 (1H, d, *J* = 13.0 Hz), 7.83 (1H, d, *J* = 8.2 Hz), 7.79 (1H, d, *J* = 8.6 Hz), 7.49 (1H, t, *J* = 7.7 Hz), 7.41 (2H, d, *J* = 8.3 Hz), 7.31 (1H, t, *J* = 7.5 Hz), 7.21 (1H, d, *J* = 10.6 Hz), 7.10 (1H, d, *J* = 8.7 Hz), 6.76–6.92 (2H, m), 6.69 (2H, d, *J* = 8.3 Hz), 5.41 (1H, d, *J* = 13.1 Hz), 3.87 (2H, q, *J* = 7.1 Hz), 3.01 (6H, s), 2.84–2.92 (2H, m), 2.73–2.82 (2H, m), 1.99 (6H, s). ^13^C{^1^H} NMR (400 MHz, CDCl_3_) *δ*: 193.1, 165.4, 150.7, 140.8, 140.0, 139.7, 131.2, 131.0, 130.6, 130.4, 130.3, 129.9, 129.7, 129.0, 128.5 (2C, Ar), 127.0, 125.5, 123.0, 122.0, 121.2, 112.3 (2C, Ar), 109.2, 92.9, 49.0, 40.4 (N(CH_3_)_2_), 37.4, 28.0 (C(CH_3_)_2_), 24.1, 23.9, 11.6. HRMS‐ESI [(M + H)^+^]: m/z calculated for C_34_H_37_N_2_O: 489.2900; found: 489.2895.

*2‐[4‐(Dimethylamino)benzylidene]‐5‐[4‐(3‐ethyl‐1,1‐dimethyl‐1H‐benzo[e]indol‐2(3H)‐ylidene)but‐2‐en‐1‐ylidene]cyclopentanone (**7b**)*: Dark solid, yield 73%. ^1^H NMR (400 MHz, CDCl_3_) *δ*: 8.03 (1H, d, *J* = 8.6 Hz), 7.81 (1H, d, *J* = 8.2 Hz), 7.76 (1H, d, *J* = 8.6 Hz), 7.35–7.59 (6H, m), 7.28 (1H, dd, *J* = 7.8, 6.3 Hz), 7.06 (1H, d, *J* = 8.7 Hz), 6.74 (2H, d, *J* = 8.5 Hz), 6.20 (1H, t, *J* = 12.9 Hz), 5.59 (1H, d, *J* = 12.4 Hz), 3.80 (2H, q, *J* = 6.9 Hz), 2.99–3.09 (8H, m), 2.80–2.88 (2H, m), 1.93 (6H, s), 1.31 (3H, t, *J* = 7.1 Hz). ^13^C{^1^H} NMR (400 MHz, CDCl_3_) *δ*: 195.0, 161.4, 150.7, 141.2, 139.8, 135.6, 135.0, 133.4, 132.5, 132.4 (2C, Ar), 130.0, 129.9, 129.7, 129.6, 129.1, 127.0, 124.7, 122.6, 121.8, 120.6, 112.1 (2C, Ar), 109.1, 96.6, 48.4, 40.3 (N(CH_3_)_2_), 37.2, 27.5 (C(CH_3_)_2_), 26.5, 24.5, 11.9. HRMS‐ESI [(M + H)^+^]: m/z calculated for C_34_H_37_N_2_O: 489.2900; found: 489.2895.

*2‐[3‐(4‐(Dimethylamino)phenyl)allylidene]‐5‐[4‐(3‐ethyl‐1,1‐dimethyl‐1,3‐dihydro‐2H‐benzo[e]indol‐2‐ylidene)but‐2‐en‐1‐ylidene]cyclopentanone (**7c**)*: Dark solid, yield 59%. ^1^H NMR (400 MHz, CDCl_3_) *δ*: 8.03 (1H, d, *J* = 8.3 Hz), 7.81 (1H, d, *J* = 8.2 Hz), 7.76 (1H, d, *J* = 8.6 Hz), 7.38–7.51 (4H, m), 7.34 (1H, d, *J* = 12.3 Hz), 7.26–7.31 (1H, m), 7.23 (1H, d, *J* = 11.7 Hz), 7.06 (1H, d, *J* = 8.9 Hz), 6.75–6.93 (2H, m), 6.69 (2H, d, *J* = 8.3 Hz), 6.18 (1H, t, *J* = 12.8 Hz), 5.59 (1H, d, *J* = 12.3 Hz), 3.80 (2H, q, *J* = 7.0 Hz), 3.02 (6H, s), 2.83–2.89 (2H, m), 2.76–2.83 (2H, m), 1.93 (6H, s), 1.31 (3H, t, *J* = 7.0 Hz). ^13^C{^1^H} NMR (400 MHz, CDCl_3_) *δ*: 194.3, 161.6, 150.9, 141.2, 140.9, 140.1, 139.0, 135.1, 134.5, 132.3, 130.1, 129.9, 129.7, 129.6, 129.1, 128.7 (2C, Ar), 127.0, 125.3, 122.7, 121.8, 121.0, 120.6, 112.3 (2C, Ar), 109.2, 96.7, 48.4, 40.4 (N(CH_3_)_2_), 37.2, 27.6 (C(CH_3_)_2_), 24.2, 24.1, 11.9. HRMS‐ESI [(M + H)^+^]: m/z calculated for C_36_H_39_N_2_O: 515.3057; found: 515.3074.

*2‐[5‐(4‐(Dimethylamino)phenyl)penta‐2,4‐dien‐1‐ylidene]‐5‐[2‐(3‐ethyl‐1,1‐dimethyl‐1,3‐dihydro‐2H‐benzo[e]indol‐2‐ylidene)ethylidene]cyclopentanone (**7d**)*: Dark solid, yield 40%. ^1^H NMR (400 MHz, CDCl_3_) *δ*: 7.91–8.10 (2H, m), 7.83 (1H, d, *J* = 8.2 Hz), 7.79 (1H, d, *J* = 8.6 Hz), 7.50 (1H, t, *J* = 7.7 Hz), 7.35 (2H, d, *J* = 8.3 Hz), 7.31 (1H, t, *J* = 7.6 Hz), 7.08–7.17 (2H, m), 6.61–6.85 (5H, m), 6.40–6.52 (1H, m), 5.41 (1H, d, *J* = 13.2 Hz), 3.79–3.98 (2H, m), 3.00 (6H, s), 2.79–2.87 (2H, m), 2.72–2.79 (2H, m), 1.98 (6H, s), 1.36 (3H, t, *J* = 7.0 Hz). ^13^C{^1^H} NMR (400 MHz, CDCl_3_) *δ*: 193.0, 165.7, 150.5, 141.0, 140.84, 140.80, 136.1, 131.0, 130.9, 130.5, 130.3, 129.9, 129.7, 129.1, 128.2 (2C, Ar), 128.1, 127.6, 127.1, 125.5, 125.1, 123.0, 122.0, 112.4 (2C, Ar), 109.2, 92.9, 49.0, 40.5 (N(CH_3_)_2_), 37.5, 28.0 (C(CH_3_)_2_), 24.1, 23.9, 11.6. HRMS‐ESI [(M + H)^+^]: m/z calculated for C_36_H_39_N_2_O: 515.3057; found: 515.3078.

##### General Synthetic Procedure for Dyes 9a–d

Dye **7** (1 mmol) and POCl_3_ (0,27 mL, 3 mmol) in 1,2‐dichloroethane (5 mL) were refluxed for 2 min. After cooling, diethyl ether (20 mL) was added. The precipitate was filtered, dissolved in methanol (30 mL) and the final product was precipitated from the hot solution with NaClO_4_ (2 mL, 10% in methanol). After cooling, the pure solid was filtered, washed with methanol, and dried at 85 °C.

*2‐[2‐(2‐Chloro‐3‐(3‐(4‐(dimethylamino)phenyl)allylidene)cyclopent‐1‐en‐1‐yl)vinyl]‐3‐ethyl‐1,1‐dimethyl‐1H‐benzo[e]indolium perchlorate (**9a**)*: Dark solid, yield 77%. ^1^H NMR (400 MHz, (CD_3_)_2_SO) *δ*: 8.42 (1H, d, *J* = 8.3 Hz), 8.28 (1H, d, *J* = 8.9 Hz), 8.21 (1H, d, *J* = 8.1 Hz), 8.15 (1H, d, *J* = 15.6 Hz), 8.09 (1H, d, *J* = 8.9 Hz), 7.79 (1H, t, *J* = 7.7 Hz), 7.71 (1H, t, *J* = 7.5 Hz), 7.48 (2H, d, *J* = 8.4 Hz), 6.88–7.02 (4H, m), 6.74 (2H, d, *J* = 8.3 Hz), 4.72 (2H, q, *J* = 7.1 Hz), 3.03 (4H, s), 2.99 (6H, s), 1.97 (6H, s), 1.49 (3H, t, *J* = 7.1 Hz). HRMS‐ESI [(M−ClO_4_)^+^]: m/z calculated for C_34_H_36_ClN_2_: 507.2562; found: 507.2563.

*2‐[2‐(2‐Chloro‐3‐(3‐(4‐(dimethylamino)phenyl)allylidene)cyclopent‐1‐en‐1‐yl)vinyl]‐3‐ethyl‐1,1‐dimethyl‐1H‐benzo[e]indolium perchlorate (**9b**)*: Dark solid, yield 58%. ^1^H NMR (400 MHz, (CD_3_)_2_SO) *δ*: 8.50 (1H, dd, *J* = 15.4, 10.9 Hz), 8.41 (1H, d, *J* = 8.4 Hz), 8.27 (1H, d, *J* = 8.9 Hz), 8.20 (1H, d, *J* = 8.1 Hz), 8.08 (1H, d, *J* = 8.9 Hz), 7.75–7.88 (2H, m), 7.71 (1H, t, *J* = 7.5 Hz), 7.48 (2H, d, *J* = 8.4 Hz), 7.36 (1H, d, *J* = 15.3 Hz), 6.90–7.07 (3H, m), 6.80 (1H, s), 4.63 (4H, q, *J* = 6.8 Hz), 3.00–3.13 (8H, m), 2.85–2.95 (2H, m), 1.97 (6H, s), 1.48 (3H, t, *J* = 6.8 Hz). HRMS‐ESI [(M−ClO_4_)^+^]: m/z calculated for C_34_H_36_ClN_2_: 507.2562; found: 507.2556.

*2‐[4‐(2‐Chloro‐3‐(3‐(4‐(dimethylamino)phenyl)allylidene)cyclopent‐1‐en‐1‐yl)buta‐1,3‐dien‐1‐yl]‐3‐ethyl‐1,1‐dimethyl‐1H‐benzo[e]indolium perchlorate (**9c**)*: Dark solid, yield 42%. ^1^H NMR (400 MHz, (CD_3_)_2_SO) *δ*: 8.50 (1H, dd, *J* = 15.2, 11.1 Hz), 8.42 (1H, d, *J* = 8.5 Hz), 8.27 (1H, d, *J* = 8.9 Hz), 8.20 (1H, d, *J* = 8.2 Hz), 8.08 (1H, d, *J* = 9.0 Hz), 7.77–7.84 (2H, m), 7.68–7.74 (1H, m), 7.46 (2H, d, *J* = 8.5 Hz), 7.36 (1H, d, *J* = 15.2 Hz), 6.99 (1H, dd, *J* = 14.7, 11.1 Hz), 6.92 (1H, dd, *J* = 15.1, 11.4 Hz), 6.77–6.86 (3H, m), 6.64 (1H, d, *J* = 11.2 Hz), 4.63 (2H, q, *J* = 7.3 Hz), 2.99 (6H, s), 2.93–2.97 (2H, m), 2.82–2.88 (2H, m), 2.54 (6H, s), 1.97 (6H, s), 1.47 (3H, t, *J* = 7.2 Hz). HRMS‐ESI [(M−ClO_4_)^+^]: m/z calculated for C_36_H_38_ClN_2_: 533.2718; found: 533.2718.

*2‐[2‐(2‐Chloro‐3‐(5‐(4‐(dimethylamino)phenyl)penta‐2,4‐dien‐1‐ylidene)cyclopent‐1‐en‐1‐yl)vinyl]‐3‐ethyl‐1,1‐dimethyl‐1H‐benzo[e]indolium perchlorate (**9d**)*: Dark solid, yield 34%. ^1^H NMR (400 MHz, (CD_3_)_2_SO) *δ*: 8.43 (1H, d, *J* = 8.3 Hz), 8.29 (1H, d, *J* = 8.8 Hz), 8.21 (1H, d, *J* = 8.0 Hz), 8.08–8.17 (2H, m), 7.80 (1H, t, *J* = 7.5 Hz), 7.72 (1H, t, *J* = 7.5 Hz), 7.49 (2H, d, *J* = 8.3 Hz), 6.91–7.11 (4H, m), 6.75–6.91 (3H, m), 6.66 (1H, t, *J* = 13.3 Hz), 4.74 (2H, q, *J* = 7.0 Hz), 3.00–3.09 (8H, m), 2.93–3.00 (2H, m), 1.93–2.02 (6H, m), 1.50 (3H, t, *J* = 7.0 Hz). HRMS‐ESI [(M−ClO_4_)^+^]: m/z calculated for C_36_H_38_ClN_2_: 533.2718; found: 533.2731.

##### General Synthetic Procedure for Dyes 10a,b

KCY **7a** or **7b** (0.34 g, 0.7 mmol), diethyl ether (40 mL), and phenyllithium solution in dibutyl ether (0.7 mL, 1.3 mmol; cas# 591‐51‐5) were refluxed for 3 h under argon. After cooling, 3 mL of 5% aqueous HClO_4_ was added to the reaction mixture. The product was filtered, dried, and recrystallized by dissolving in DMF and precipitating with hot methanol.

*2‐[2‐(3‐(3‐(4‐(Dimethylamino)phenyl)allylidene)‐2‐phenylcyclopent‐1‐en‐1‐yl)vinyl]‐3‐ethyl‐1,1‐dimethyl‐1H‐benzo[e]indolium perchlorate (**10a**)*: Dark solid, yield 25%. ^1^H NMR (600 MHz, (CD_3_)_2_SO) *δ*: 8.34 (1H, d, *J* = 8.5 Hz), 8.23 (1H, d, *J* = 8.9 Hz), 8.16 (1H, d, *J* = 8.1 Hz), 8.02 (1H, d, *J* = 8.9 Hz), 7.81 (1H, d, *J* = 15.4 Hz), 7.68–7.72 (1H, m), 7.61–7.67 (4H, m), 7.46 (2H, d, *J* = 8.4 Hz), 7.39–7.43 (2H, m), 7.04 (1H, dd, *J* = 15.3, 11.6 Hz), 6.88 (1H, d, *J* = 15.4 Hz), 6.72–6.84 (3H, m), 6.47 (1H, d, *J* = 11.6 Hz), 4.66 (2H, q, *J* = 7.2 Hz), 3.12 (4H, s), 2.99 (6H, s), 1.70 (6H, s), 1.44 (3H, t, *J* = 7.2 Hz). HRMS‐ESI [(M−ClO_4_)^+^]: m/z calculated for C_40_H_41_N_2_: 549.3264; found: 549.3257.

*2‐[4‐(3‐(4‐(Dimethylamino)benzylidene)‐2‐phenylcyclopent‐1‐en‐1‐yl)buta‐1,3‐dien‐1‐yl]‐3‐ethyl‐1,1‐dimethyl‐1H‐benzo[e]indolium perchlorate (**10b**)*: Dark solid, yield 22%. ^1^H NMR (600 MHz, (CD_3_)_2_SO) *δ*: 8.37 (1H, dd, *J* = 15.1, 11.1 Hz), 8.32 (1H, d, *J* = 8.5 Hz), 8.24 (1H, d, *J* = 8.9 Hz), 8.17 (1H, d, *J* = 8.2 Hz), 7.76 (1H, t, *J* = 7.7 Hz), 7.66–7.70 (1H, m), 7.57–7.61 (2H, m), 7.51–7.55 (1H, m), 7.41 (1H, d, *J* = 14.6 Hz), 7.27–7.36 (4H, m), 7.24 (1H, d, *J* = 15.1 Hz), 6.97 (1H, dd, *J* = 14.6, 11.1 Hz), 6.80 (2H, br. s), 6.25 (1H, s), 4.59 (2H, q, *J* = 7.3 Hz), 3.09–3.19 (2H, m), 2.94–3.05 (8H, m), 1.89 (6H, s), 1.45 (3H, t, *J *= 7.3 Hz). HRMS‐ESI [(M−ClO_4_)^+^]: m/z calculated for C_40_H_41_N_2_: 549.3264; found: 549.3254.

## Conflict of Interest

The authors declare no conflict of interest.

## Author Contributions

**Sviatoslava O. Melnychuk**: data curation (equal), investigation (equal), validation (equal), visualization (equal). **Sergii V. Popov:** conceptualization (lead), investigation (equal), resources (lead). **Serhii B. Babii**: data curation (equal), investigation (equal). **Andrii V. Kulinich**: data curation (equal), investigation (equal), validation (equal), visualization (equal), writing—original draft (lead), writing—review and editing (lead).

## Supporting information

Supplementary Material

## Data Availability

The data that support the findings of this study are available in the supplementary material of this article.
