# Supplementary material for: Asymmetric Ketocyanine Dyes with an Extended Polymethine Chain
Source: ChemistryOpen. 2025 Jun 4;14(9):e202500096. doi: 10.1002/open.202500096 (PMC12409850; doi:10.1002/open.202500096)
Supplement: Supplementary file 1 — Supplementary Material [file OPEN-14-e202500096-s001.pdf]

Asymmetric ketocyanine dyes with an extended polymethine chain

Sviatoslava O. Melnychuk,[b,c] Sergii V. Popov,[b] Serhii B. Babii,[b] and Andrii V. Kulinich*[a]

[a] Institute of Organic Chemistry, National Academy of Sciences of Ukraine,
5 Akademika Kukharya st., 02094 Kyiv, Ukraine;
E-mail: andrii.kulinich@gmail.com

[b] Spectrum Info LLC,
11 Myrnoho Panasa st., Office 2/28, 01011 Kyiv, Ukraine

[c] Enamine Ltd.,
78 Winston Churchill st., 02094 Kyiv, Ukraine

Supporting information includes:

Comments on fluorescence spectral measurements 2

Table S1. Some characteristics of the solvents used in the study 2

Table S2. TD-DFT and experimental absorption data of dyes **7a-d** in dichloromethane 2

Cartesian atomic coordinates (in Å) and final energies for the optimized ground-state geometries of molecules **7a-d** and **9a-d** (without counterion, with *N*-ethyl reduced to *N*-methyl) 3-12

^{1}H NMR spectra of the synthesized compounds (**3a-d**, **4a,b**, **5a-d**, **7a-d**, **9a-d**, **10a,b**) 13-32

^{13}C NMR spectra of the synthesized compounds (**3a-d**, **4a,b**, **5a-d**, **7a-d**) 33-46

HRMS spectra of the synthesized compounds (**3a,c,d**, **4a,b**, **5a-d**, **7a-d**, **9a-d**, **10a,b**) 47-65

Comments on fluorescence spectral measurements

FQYs are given in Table 2 in the paper without the experimental error, which is typically ca. 10% of the obtained value. For dyes **9a-b** and **10a,b** the experimental error should be even greater (we cannot evaluate it more precisely) since, first, their fluorescence bands are much distanced from the fluorescence band of the reference fluorophore (indotricarbocyanine), and, second, the long-wavelength slopes of their fluorescence bands have not been measured deeply enough, so their integral intensity must be underestimated. It should also be noted that due to the lower sensitivity of the fluorimeter detector in the region above 950 nm and the use of wider slit widths (15-20 nm) during their fluorescence spectra recording (to enhance the signal-to-noise ratio), the accuracy of determining the maxima of their fluorescence bands is ±3 nm.

Table S1. Some characteristics of the solvents used in the study

Solvent	ε_D	n_D	E_T^N	α	β	π^*	η (cP)
n-Hexane	1.88	1.386	0.009	—	—	–0.08	0.31
Toluene	2.38	1.497	0.099	0.00	0.11	0.54	0.59
EtOAc	6.02	1.372	0.228	0.00	0.45	0.55	0.45
DCM (CH_2Cl_2)	8.93	1.424	0.309	0.13	0.10	0.82	0.44
DMF	36.7	1.431	0.386	0.00	0.69	0.88	0.92
Ethanol	24.6	1.361	0.654	0.86	0.75	0.54	1.10

Table S2. TD-DFT [PCM$_{CH2Cl2}$/TD-B3LYP/6-31G(d,p)] and experimental absorption data of dyes **7a-d** in dichloromethane

Dye	E(S$_0$), Hartree	E(S$_1^{FC}$), Hartree	ΔE, eV	ΔE, nm	λ^a_{max}, nm (experimental)
**7a**	−1462.764106	−1462.681605	2.245	552	538
**7b**	−1462.762877	−1462.681205	2.222	558	550
**7c**	−1540.174462	−1540.099278	2.046	606	572
**7d**	−1540.175406	−1540.098123	2.103	590	548

Cartesian atomic coordinates (in Å) and final energies for the optimized ground-state geometries of molecules **7a-d** and **9a-d**

7a, PCM_{CH2Cl2}/DFT-B3LYP/6-31G(d,p) optimization; final energy -1462.76410609 Hartree

C	6.014703000000	-1.238143000000	-0.009417000000
C	6.321847000000	0.113581000000	-0.023776000000
N	5.146119000000	0.876946000000	-0.024601000000
C	4.026984000000	0.071785000000	-0.011039000000
C	4.492545000000	-1.398206000000	0.000359000000
C	7.053659000000	-2.205684000000	-0.005958000000
C	8.414884000000	-1.727508000000	-0.017857000000
C	8.669944000000	-0.329797000000	-0.032244000000
C	7.649992000000	0.593830000000	-0.035391000000
C	4.012038000000	-2.104657000000	1.292815000000
C	3.998067000000	-2.128904000000	-1.273215000000
C	5.087583000000	2.327311000000	-0.038273000000
C	2.753701000000	0.588825000000	-0.009325000000
C	1.527704000000	-0.139766000000	0.003926000000
C	0.278868000000	0.410288000000	0.003389000000
C	-0.092734000000	1.875214000000	-0.009469000000
C	-1.651750000000	1.915634000000	-0.026985000000
C	-2.103039000000	0.472484000000	0.007933000000
C	-0.927344000000	-0.426820000000	0.018876000000
O	-0.963731000000	-1.668291000000	0.037912000000
C	-3.371891000000	-0.011231000000	0.026296000000
C	-4.579421000000	0.764079000000	0.017732000000
C	-5.817271000000	0.197711000000	0.038701000000
C	-7.107888000000	0.860782000000	0.033093000000
C	-8.287016000000	0.087218000000	0.062103000000
C	-9.550407000000	0.657138000000	0.058688000000
C	-9.712246000000	2.065538000000	0.026802000000
C	-8.528562000000	2.851550000000	-0.005174000000
C	-7.276175000000	2.262728000000	-0.001179000000
N	-10.958124000000	2.647110000000	0.027526000000
C	-11.092248000000	4.095287000000	-0.034620000000
C	-12.152699000000	1.815475000000	0.030260000000
C	6.849195000000	-3.617592000000	0.008353000000
C	7.911703000000	-4.493719000000	0.010643000000
C	9.245280000000	-4.018364000000	-0.001135000000
C	9.484002000000	-2.663165000000	-0.015001000000
H	9.702041000000	0.009024000000	-0.040933000000
H	7.872371000000	1.654491000000	-0.046434000000
H	4.402892000000	-3.123128000000	1.347401000000
H	2.922309000000	-2.159582000000	1.332431000000
H	4.357870000000	-1.563129000000	2.177719000000
H	2.907987000000	-2.184759000000	-1.299735000000
H	4.333900000000	-1.604046000000	-2.171901000000
H	4.388688000000	-3.148143000000	-1.312985000000
H	6.094530000000	2.738517000000	-0.047376000000
H	4.558231000000	2.683499000000	-0.928490000000
H	4.567395000000	2.700625000000	0.850305000000
H	2.663706000000	1.670778000000	-0.019179000000
H	1.545945000000	-1.225947000000	0.015558000000
H	0.303607000000	2.398085000000	0.870201000000
H	0.325075000000	2.391351000000	-0.882876000000
H	-2.021179000000	2.427047000000	-0.924032000000
H	-2.042014000000	2.479992000000	0.828197000000
H	-3.472565000000	-1.096827000000	0.050140000000
H	-4.478804000000	1.847225000000	-0.006497000000
H	-5.861418000000	-0.891784000000	0.063255000000
H	-8.203669000000	-0.996715000000	0.087424000000
H	-10.416420000000	0.007689000000	0.080562000000
H	-8.594375000000	3.932012000000	-0.033986000000
H	-6.405190000000	2.910801000000	-0.026702000000
H	-10.609099000000	4.584372000000	0.820637000000
H	-10.655644000000	4.509121000000	-0.953248000000
H	-12.149670000000	4.357003000000	-0.016515000000
H	-12.197010000000	1.172920000000	0.918768000000
H	-13.034184000000	2.455778000000	0.034999000000

H	-12.205483000000	1.170728000000	-0.857069000000
H	5.841708000000	-4.014434000000	0.017627000000
H	7.723659000000	-5.563378000000	0.021627000000
H	10.071619000000	-4.722413000000	0.000849000000
H	10.502640000000	-2.283837000000	-0.024110000000

7b, PCM$_{CH2Cl2}$/DFT-B3LYP/6-31G(d,p) optimization; final energy -1462.76287718 Hartree

C	6.269190000000	-0.927042000000	-0.007636000000
C	6.171139000000	-2.310060000000	-0.010950000000
N	4.825683000000	-2.700496000000	-0.000720000000
C	3.987052000000	-1.604560000000	0.005887000000
C	4.858646000000	-0.332388000000	0.004473000000
C	7.543718000000	-0.301835000000	-0.015323000000
C	8.708165000000	-1.153859000000	-0.027435000000
C	8.547254000000	-2.565622000000	-0.031448000000
C	7.303598000000	-3.154285000000	-0.023599000000
C	4.592558000000	0.501415000000	-1.273918000000
C	4.610537000000	0.492157000000	1.292522000000
C	4.350161000000	-4.071866000000	0.002540000000
C	2.619400000000	-1.730517000000	0.011046000000
C	1.649759000000	-0.685048000000	0.021348000000
C	0.293688000000	-0.889119000000	0.023482000000
C	-0.653871000000	0.177037000000	0.035819000000
C	-2.011982000000	0.059081000000	0.036332000000
C	-2.834397000000	-1.207742000000	0.024297000000
C	-4.323180000000	-0.746257000000	-0.014854000000
C	-4.296585000000	0.764598000000	0.031381000000
C	-2.889942000000	1.238444000000	0.052696000000
O	-2.523990000000	2.423646000000	0.079492000000
C	-5.309540000000	1.667189000000	0.053413000000
C	-6.749985000000	1.501644000000	0.038009000000
C	-7.561580000000	2.657300000000	0.094107000000
C	-8.944653000000	2.600320000000	0.084074000000
C	-9.620048000000	1.354120000000	0.017530000000
C	-8.814173000000	0.187328000000	-0.046596000000
C	-7.430776000000	0.264795000000	-0.034585000000
N	-10.992910000000	1.279688000000	0.016566000000
C	-11.656447000000	-0.009371000000	-0.115964000000
C	-11.790801000000	2.497460000000	0.017738000000
C	7.757340000000	1.108892000000	-0.011966000000
C	9.028151000000	1.639560000000	-0.020085000000
C	10.166774000000	0.798228000000	-0.032130000000
C	10.002428000000	-0.568080000000	-0.035636000000
H	9.436819000000	-3.189094000000	-0.041396000000
H	7.209334000000	-4.234000000000	-0.028193000000
H	5.261611000000	1.363359000000	-1.322527000000
H	3.565528000000	0.871583000000	-1.300891000000
H	4.758224000000	-0.104683000000	-2.168909000000
H	5.280519000000	1.353559000000	1.337999000000
H	3.584141000000	0.862486000000	1.336457000000
H	4.788437000000	-0.120437000000	2.180708000000
H	5.195345000000	-4.756420000000	0.014606000000
H	3.735668000000	-4.264075000000	0.888421000000
H	3.749758000000	-4.275797000000	-0.890566000000
H	2.215290000000	-2.739411000000	0.006639000000
H	1.989559000000	0.347111000000	0.028735000000
H	-0.080767000000	-1.911966000000	0.016153000000
H	-0.264541000000	1.195968000000	0.045743000000
H	-2.594402000000	-1.839335000000	-0.839436000000
H	-2.638820000000	-1.820343000000	0.913117000000
H	-4.886198000000	-1.165454000000	0.827600000000
H	-4.818244000000	-1.108200000000	-0.924445000000
H	-4.970309000000	2.703322000000	0.090745000000
H	-7.080574000000	3.630970000000	0.146061000000
H	-9.506080000000	3.525176000000	0.126740000000
H	-9.276917000000	-0.789726000000	-0.108203000000
H	-6.870935000000	-0.660462000000	-0.087744000000
H	-11.381504000000	-0.688995000000	0.699829000000
H	-12.735198000000	0.138711000000	-0.077883000000
H	-11.413292000000	-0.503696000000	-1.066290000000
H	-11.604727000000	3.114038000000	-0.872074000000
H	-12.847505000000	2.232321000000	0.030814000000

H	-11.586924000000	3.110964000000	0.903789000000
H	6.908299000000	1.781071000000	-0.002715000000
H	9.158087000000	2.717879000000	-0.017227000000
H	11.161649000000	1.232585000000	-0.038484000000
H	10.867513000000	-1.226206000000	-0.044812000000

7b (Z-isomer), PCM_{CH2Cl2}/DFT-B3LYP/6-31G(d,p) optimization; final energy -1462.75684573 Hartree

C	-5.774357000000	1.426785000000	0.038079000000
C	-6.422226000000	0.209298000000	0.181482000000
N	-5.490551000000	-0.836880000000	0.195478000000
C	-4.201421000000	-0.359870000000	0.070287000000
C	-4.266721000000	1.176228000000	-0.048940000000
C	-6.522276000000	2.632684000000	-0.004655000000
C	-7.957530000000	2.535678000000	0.107593000000
C	-8.567273000000	1.260820000000	0.255269000000
C	-7.826227000000	0.102143000000	0.294065000000
C	-3.520750000000	1.840546000000	1.135311000000
C	-3.700933000000	1.635046000000	-1.416407000000
C	-5.812560000000	-2.246398000000	0.324110000000
C	-3.110279000000	-1.193857000000	0.065969000000
C	-1.737221000000	-0.829266000000	-0.058753000000
C	-0.696539000000	-1.722115000000	-0.047520000000
C	0.668372000000	-1.326730000000	-0.172182000000
C	1.757532000000	-2.146519000000	-0.171201000000
C	1.795806000000	-3.648397000000	-0.030646000000
C	3.233790000000	-4.032554000000	-0.456120000000
C	4.062325000000	-2.760033000000	-0.322444000000
C	3.125775000000	-1.609638000000	-0.290588000000
O	3.396413000000	-0.397451000000	-0.349809000000
C	5.423215000000	-2.820713000000	-0.242116000000
C	6.503873000000	-1.856376000000	-0.122585000000
C	7.814880000000	-2.369491000000	0.018219000000
C	8.927655000000	-1.555593000000	0.141790000000
C	8.796135000000	-0.142930000000	0.131716000000
C	7.485061000000	0.380273000000	-0.020176000000
C	6.381254000000	-0.447642000000	-0.141683000000
N	9.889090000000	0.681107000000	0.262008000000
C	9.731039000000	2.126219000000	0.180518000000
C	11.226255000000	0.113267000000	0.354205000000
C	-5.957772000000	3.934875000000	-0.150957000000
C	-6.752497000000	5.059123000000	-0.184078000000
C	-8.160256000000	4.957310000000	-0.073240000000
C	-8.743014000000	3.718812000000	0.069333000000
H	-9.649154000000	1.209958000000	0.339576000000
H	-8.317017000000	-0.857178000000	0.409218000000
H	-3.626582000000	2.927110000000	1.101608000000
H	-2.454392000000	1.606179000000	1.115287000000
H	-3.926728000000	1.491782000000	2.088890000000
H	-3.812097000000	2.714444000000	-1.541107000000
H	-2.639839000000	1.394077000000	-1.509051000000
H	-4.232990000000	1.144364000000	-2.236117000000
H	-6.891057000000	-2.376813000000	0.379096000000
H	-5.439965000000	-2.805885000000	-0.540402000000
H	-5.364260000000	-2.665517000000	1.231425000000
H	-3.300945000000	-2.258731000000	0.170330000000
H	-1.480046000000	0.220779000000	-0.168862000000
H	-0.919081000000	-2.782398000000	0.064995000000
H	0.874540000000	-0.261318000000	-0.281176000000
H	1.614315000000	-3.943355000000	1.012262000000
H	1.035789000000	-4.155241000000	-0.635046000000
H	3.229844000000	-4.366406000000	-1.502666000000
H	3.639278000000	-4.859023000000	0.135793000000
H	5.805666000000	-3.843810000000	-0.256168000000
H	7.958918000000	-3.447412000000	0.030940000000
H	9.902890000000	-2.014897000000	0.244218000000
H	7.330321000000	1.452064000000	-0.047328000000
H	5.393634000000	-0.018224000000	-0.259904000000
H	9.058690000000	2.502180000000	0.961211000000
H	10.702442000000	2.600005000000	0.318951000000
H	9.332078000000	2.443794000000	-0.792285000000
H	11.494899000000	-0.459891000000	-0.543619000000
H	11.949902000000	0.919517000000	0.470290000000

H	11.321704000000	-0.551637000000	1.221332000000
H	-4.884452000000	4.048618000000	-0.238712000000
H	-6.292708000000	6.036661000000	-0.296764000000
H	-8.772422000000	5.853394000000	-0.101113000000
H	-9.822539000000	3.625245000000	0.155553000000

7c, PCM$_{CH2Cl2}$/DFT-B3LYP/6-31G(d,p) optimization; final energy -1540.17446237 Hartree

C	-7.329610000000	0.035850000000	-0.002356000000
C	-7.430900000000	1.418407000000	-0.024897000000
N	-6.155170000000	1.997694000000	-0.031201000000
C	-5.168238000000	1.033491000000	-0.013532000000
C	-5.848485000000	-0.350293000000	0.007346000000
C	-8.501317000000	-0.765595000000	0.007897000000
C	-9.775890000000	-0.089449000000	-0.006251000000
C	-9.819137000000	1.330636000000	-0.029314000000
C	-8.672685000000	2.091456000000	-0.038778000000
C	-5.472323000000	-1.155060000000	-1.262036000000
C	-5.478065000000	-1.112597000000	1.304285000000
C	-5.881456000000	3.423017000000	-0.053883000000
C	-3.832419000000	1.353648000000	-0.015490000000
C	-2.724332000000	0.456634000000	-0.000450000000
C	-1.410195000000	0.849236000000	-0.004955000000
C	-0.324034000000	-0.075014000000	0.006742000000
C	1.005378000000	0.227295000000	0.003478000000
C	1.648681000000	1.595303000000	-0.018749000000
C	3.182814000000	1.342318000000	0.095756000000
C	3.357943000000	-0.158205000000	0.029626000000
C	2.036246000000	-0.822403000000	0.007533000000
O	1.838682000000	-2.047948000000	-0.006809000000
C	4.514336000000	-0.870431000000	-0.001869000000
C	5.845612000000	-0.336593000000	0.014908000000
C	6.954015000000	-1.126728000000	-0.025317000000
C	8.346468000000	-0.720712000000	-0.015854000000
C	9.357334000000	-1.703218000000	-0.069114000000
C	10.705720000000	-1.383418000000	-0.064974000000
C	11.131881000000	-0.032092000000	-0.005859000000
C	10.118939000000	0.963517000000	0.049187000000
C	8.777745000000	0.623093000000	0.043637000000
N	12.465115000000	0.302307000000	-0.001935000000
C	12.871992000000	1.698164000000	0.071000000000
C	13.480073000000	-0.739485000000	-0.059809000000
C	-8.510389000000	-2.192177000000	0.031342000000
C	-9.691985000000	-2.899531000000	0.040047000000
C	-10.939511000000	-2.230271000000	0.025833000000
C	-10.972784000000	-0.854695000000	0.003202000000
H	-10.789084000000	1.819819000000	-0.039586000000
H	-8.733866000000	3.173371000000	-0.056404000000
H	-6.011504000000	-2.104332000000	-1.294770000000
H	-4.403138000000	-1.375189000000	-1.291443000000
H	-5.727474000000	-0.591209000000	-2.163556000000
H	-4.408453000000	-1.328418000000	1.347029000000
H	-5.740054000000	-0.520568000000	2.185564000000
H	-6.015030000000	-2.061787000000	1.364571000000
H	-6.816308000000	3.978981000000	-0.065457000000
H	-5.311094000000	3.720797000000	0.832418000000
H	-5.306444000000	3.691523000000	-0.946506000000
H	-3.576287000000	2.409714000000	-0.031115000000
H	-2.914594000000	-0.613170000000	0.014330000000
H	-1.182211000000	1.914271000000	-0.020341000000
H	-0.569705000000	-1.137771000000	0.018871000000
H	1.411458000000	2.127937000000	-0.948477000000
H	1.287615000000	2.231749000000	0.797599000000
H	3.572260000000	1.741139000000	1.040755000000
H	3.732804000000	1.854420000000	-0.701975000000
H	4.409509000000	-1.954925000000	-0.045853000000
H	5.951417000000	0.745276000000	0.061406000000
H	6.790590000000	-2.204105000000	-0.071147000000
H	9.069583000000	-2.750832000000	-0.115242000000
H	11.432813000000	-2.184531000000	-0.107169000000
H	10.388817000000	2.011121000000	0.096056000000
H	8.045604000000	1.423906000000	0.086933000000
H	12.502156000000	2.276177000000	-0.785723000000

H	12.510080000000	2.181188000000	0.987830000000
H	13.960045000000	1.753489000000	0.069188000000
H	13.391428000000	-1.342921000000	-0.972333000000
H	14.466983000000	-0.278028000000	-0.056491000000
H	13.419193000000	-1.417214000000	0.801732000000
H	-7.573750000000	-2.735634000000	0.042765000000
H	-9.665955000000	-3.985194000000	0.058017000000
H	-11.861844000000	-2.802754000000	0.032857000000
H	-11.923189000000	-0.327314000000	-0.007907000000

7d, PCM$_{CH2Cl2}$/DFT-B3LYP/6-31G(d,p) optimization; final energy -1540.17540589 Hartree

C	-7.169392000000	0.003035000000	0.002139000000
C	-7.217365000000	1.388339000000	-0.010590000000
N	-5.919065000000	1.917276000000	-0.017422000000
C	-4.971248000000	0.916525000000	-0.009624000000
C	-5.704346000000	-0.439890000000	0.003785000000
C	-8.371545000000	-0.752200000000	0.010980000000
C	-9.618704000000	-0.026779000000	0.006179000000
C	-9.606794000000	1.393990000000	-0.007090000000
C	-8.431588000000	2.109647000000	-0.015496000000
C	-5.358155000000	-1.225444000000	1.293530000000
C	-5.362629000000	-1.248783000000	-1.272674000000
C	-5.589800000000	3.331100000000	-0.030963000000
C	-3.623296000000	1.185577000000	-0.013776000000
C	-2.555893000000	0.240372000000	-0.007193000000
C	-1.225883000000	0.547286000000	-0.009123000000
C	-0.587091000000	1.916921000000	-0.020300000000
C	0.951118000000	1.665368000000	0.028425000000
C	1.125219000000	0.163699000000	0.001698000000
C	-0.198027000000	-0.500444000000	-0.005967000000
O	-0.393336000000	-1.727169000000	-0.009377000000
C	2.282559000000	-0.548426000000	-0.010943000000
C	3.610289000000	-0.010639000000	-0.003107000000
C	4.734850000000	-0.784638000000	-0.017577000000
C	6.077255000000	-0.282894000000	-0.009260000000
C	7.173904000000	-1.089822000000	-0.024721000000
C	8.571755000000	-0.701651000000	-0.017800000000
C	9.570266000000	-1.697668000000	-0.045729000000
C	10.922866000000	-1.395109000000	-0.040833000000
C	11.366537000000	-0.048726000000	-0.010013000000
C	10.366319000000	0.960197000000	0.023815000000
C	9.020561000000	0.637162000000	0.018308000000
N	12.704838000000	0.268962000000	-0.014504000000
C	13.128759000000	1.658406000000	0.079841000000
C	13.705709000000	-0.787517000000	0.014026000000
C	-8.435820000000	-2.177377000000	0.024353000000
C	-9.643988000000	-2.838281000000	0.032467000000
C	-10.864535000000	-2.120836000000	0.027721000000
C	-10.844533000000	-0.744906000000	0.014812000000
H	-10.556957000000	1.920530000000	-0.010591000000
H	-8.450652000000	3.193232000000	-0.025594000000
H	-5.933250000000	-2.152294000000	1.350179000000
H	-5.938572000000	-2.176053000000	-1.310778000000
H	-4.297986000000	-1.484308000000	1.327047000000
H	-4.302777000000	-1.509092000000	-1.304888000000
H	-5.591239000000	-0.629513000000	2.180343000000
H	-5.598088000000	-0.668774000000	-2.169359000000
H	-6.502047000000	3.923439000000	-0.034676000000
H	-5.004485000000	3.599162000000	0.855042000000
H	-5.007790000000	3.582887000000	-0.923881000000
H	-3.332064000000	2.231459000000	-0.023466000000
H	-2.776792000000	-0.823330000000	-0.000173000000
H	-0.915352000000	2.525757000000	0.831223000000
H	-0.863233000000	2.480353000000	-0.920752000000
H	1.390510000000	2.098949000000	0.935035000000
H	1.458913000000	2.145897000000	-0.816008000000
H	3.723479000000	1.072599000000	0.016210000000
H	4.614246000000	-1.868693000000	-0.036575000000
H	2.179479000000	-1.633762000000	-0.029089000000
H	6.195425000000	0.799497000000	0.010073000000
H	6.996842000000	-2.165731000000	-0.045703000000
H	9.269580000000	-2.742431000000	-0.070654000000

H	11.639338000000	-2.206614000000	-0.060365000000
H	10.649192000000	2.004938000000	0.055945000000
H	8.299048000000	1.448313000000	0.045597000000
H	12.741952000000	2.255118000000	-0.755316000000
H	12.797638000000	2.129197000000	1.015452000000
H	14.216857000000	1.702680000000	0.045709000000
H	13.628320000000	-1.405306000000	0.919050000000
H	14.698822000000	-0.339820000000	-0.006244000000
H	13.617101000000	-1.449418000000	-0.856262000000
H	-7.520790000000	-2.756473000000	0.028270000000
H	-9.660212000000	-3.924226000000	0.042639000000
H	-11.808393000000	-2.657125000000	0.034248000000
H	-11.773792000000	-0.180929000000	0.011013000000

9a, PCM$_{CH2Cl2}$/DFT-B3LYP/6-31G(d,p) optimization; final energy -1847.58482499 Hartree

C	6.117081000000	-0.379153000000	0.003017000000
C	6.042757000000	-1.760745000000	0.020015000000
N	4.687847000000	-2.160201000000	0.018106000000
C	3.857717000000	-1.093329000000	0.005778000000
C	4.700980000000	0.189544000000	-0.008612000000
C	7.383823000000	0.264404000000	-0.000740000000
C	8.553907000000	-0.577627000000	0.015350000000
C	8.411716000000	-1.992873000000	0.035037000000
C	7.177309000000	-2.598648000000	0.037815000000
C	4.432426000000	0.992276000000	-1.308597000000
C	4.425643000000	1.027681000000	1.267236000000
C	4.239646000000	-3.550275000000	0.027455000000
C	2.465627000000	-1.246090000000	0.008105000000
C	1.521329000000	-0.221572000000	-0.006933000000
C	0.139309000000	-0.433937000000	-0.002359000000
C	-0.561094000000	-1.781764000000	0.018622000000
C	-2.080185000000	-1.450495000000	0.014170000000
C	-2.160531000000	0.068055000000	-0.007309000000
C	-0.837644000000	0.562985000000	-0.016002000000
Cl	-0.466399000000	2.266056000000	-0.041068000000
C	-3.311467000000	0.833121000000	-0.016487000000
C	-4.627533000000	0.323870000000	-0.007971000000
C	-5.727022000000	1.154819000000	-0.017598000000
C	-7.110738000000	0.792411000000	-0.011411000000
C	-8.095110000000	1.812912000000	-0.023785000000
C	-9.445558000000	1.534954000000	-0.019191000000
C	-9.911301000000	0.189418000000	-0.001878000000
C	-8.926806000000	-0.843737000000	0.011058000000
C	-7.582137000000	-0.546310000000	0.006386000000
N	-11.243043000000	-0.102079000000	0.002149000000
C	-11.698423000000	-1.489457000000	0.018919000000
C	-12.233187000000	0.971024000000	-0.009359000000
C	7.576659000000	1.676033000000	-0.019370000000
C	8.841836000000	2.220390000000	-0.022175000000
C	9.987541000000	1.390209000000	-0.006390000000
C	9.841287000000	0.021757000000	0.011992000000
H	9.309327000000	-2.603628000000	0.048693000000
H	7.095303000000	-3.678583000000	0.055112000000
H	4.608254000000	0.369809000000	-2.189840000000
H	3.404710000000	1.357858000000	-1.347429000000
H	5.099053000000	1.854462000000	-1.366714000000
H	4.595881000000	0.429446000000	2.166202000000
H	5.092498000000	1.890781000000	1.305537000000
H	3.398072000000	1.394914000000	1.290242000000
H	5.102126000000	-4.210469000000	0.005353000000
H	3.661831000000	-3.755449000000	0.932244000000
H	3.622346000000	-3.752050000000	-0.851359000000
H	2.088754000000	-2.262713000000	0.024191000000
H	1.848153000000	0.811400000000	-0.023348000000
H	-0.276387000000	-2.383415000000	-0.851620000000
H	-0.275702000000	-2.356131000000	0.906886000000
H	-2.582163000000	-1.855189000000	0.899102000000
H	-2.582337000000	-1.879947000000	-0.858949000000
H	-3.196143000000	1.915076000000	-0.031529000000
H	-4.761900000000	-0.754337000000	0.006343000000
H	-5.527869000000	2.226328000000	-0.031853000000
H	-7.772539000000	2.850689000000	-0.037389000000

H	-10.150234000000	2.356070000000	-0.029227000000
H	-9.231123000000	-1.882164000000	0.024886000000
H	-6.873950000000	-1.368553000000	0.016772000000
H	-11.347038000000	-2.037869000000	-0.862730000000
H	-11.350679000000	-2.015632000000	0.915487000000
H	-12.786971000000	-1.506073000000	0.016835000000
H	-12.136815000000	1.615247000000	0.872295000000
H	-12.138066000000	1.595039000000	-0.905561000000
H	-13.230565000000	0.534809000000	-0.003701000000
H	6.720239000000	2.338463000000	-0.031894000000
H	8.961207000000	3.299387000000	-0.036708000000
H	10.977392000000	1.835402000000	-0.008873000000
H	10.714361000000	-0.624776000000	0.024216000000

9b, PCM$_{CH2Cl2}$/DFT-B3LYP/6-31G(d,p) optimization; final energy -1847.58295696 Hartree

C	6.275665000000	-1.260046000000	0.006946000000
C	6.650304000000	0.071365000000	-0.022037000000
N	5.495381000000	0.885196000000	-0.061909000000
C	4.367526000000	0.143171000000	-0.049314000000
C	4.752127000000	-1.342593000000	-0.010437000000
C	7.267620000000	-2.276651000000	0.045956000000
C	8.646163000000	-1.855268000000	0.055783000000
C	8.967165000000	-0.469804000000	0.028393000000
C	7.993691000000	0.500522000000	-0.009986000000
C	4.213277000000	-2.000860000000	1.286360000000
C	4.246159000000	-2.062493000000	-1.287997000000
C	5.520732000000	2.345669000000	-0.105288000000
C	3.097364000000	0.735917000000	-0.061397000000
C	1.873059000000	0.076354000000	-0.064254000000
C	0.632491000000	0.733558000000	-0.063841000000
C	-0.570229000000	0.043378000000	-0.067889000000
C	-1.841278000000	0.641621000000	-0.057716000000
C	-2.115808000000	2.133495000000	-0.034998000000
C	-3.664016000000	2.260211000000	-0.010811000000
C	-4.195175000000	0.834510000000	-0.042115000000
C	-3.058282000000	-0.025486000000	-0.064288000000
Cl	-3.189405000000	-1.764563000000	-0.096072000000
C	-5.496372000000	0.389302000000	-0.049431000000
C	-6.737253000000	1.105948000000	-0.025114000000
C	-7.939743000000	0.350183000000	-0.054596000000
C	-9.187161000000	0.935231000000	-0.032531000000
C	-9.325741000000	2.351105000000	0.023528000000
C	-8.126920000000	3.119898000000	0.053504000000
C	-6.886052000000	2.517262000000	0.029572000000
N	-10.553315000000	2.946235000000	0.048378000000
C	-10.669439000000	4.400040000000	0.109083000000
C	-11.767119000000	2.135637000000	0.022887000000
C	6.995974000000	-3.674952000000	0.076144000000
C	8.018746000000	-4.596683000000	0.112707000000
C	9.370507000000	-4.178675000000	0.121811000000
C	9.672326000000	-2.836118000000	0.094209000000
H	10.013616000000	-0.180715000000	0.039480000000
H	8.263550000000	1.549317000000	-0.026575000000
H	4.562462000000	-1.458526000000	2.168851000000
H	3.121966000000	-2.014607000000	1.302862000000
H	4.564268000000	-3.031343000000	1.362834000000
H	4.609228000000	-1.556425000000	-2.186227000000
H	4.608591000000	-3.091651000000	-1.311276000000
H	3.155637000000	-2.089239000000	-1.326671000000
H	6.546969000000	2.688129000000	-0.204541000000
H	4.950245000000	2.703470000000	-0.965093000000
H	5.095691000000	2.760855000000	0.812216000000
H	3.062947000000	1.820920000000	-0.064361000000
H	1.845506000000	-1.008577000000	-0.066592000000
H	0.625244000000	1.821076000000	-0.058087000000
H	-0.532971000000	-1.044118000000	-0.077916000000
H	-1.657557000000	2.602489000000	0.842407000000
H	-1.684546000000	2.622486000000	-0.915391000000
H	-4.029585000000	2.832952000000	-0.869354000000
H	-4.003706000000	2.782564000000	0.889670000000
H	-5.613127000000	-0.691781000000	-0.078921000000
H	-7.872336000000	-0.733698000000	-0.096867000000

H	-10.063762000000	0.301189000000	-0.057898000000
H	-8.176805000000	4.200193000000	0.097379000000
H	-6.014435000000	3.157188000000	0.056556000000
H	-10.207000000000	4.803197000000	1.017820000000
H	-10.199297000000	4.877281000000	-0.758677000000
H	-11.723464000000	4.672597000000	0.115463000000
H	-11.827587000000	1.531065000000	-0.889680000000
H	-11.819630000000	1.462758000000	0.886922000000
H	-12.634493000000	2.793047000000	0.051994000000
H	5.972101000000	-4.027054000000	0.070332000000
H	7.784784000000	-5.656599000000	0.134736000000
H	10.164390000000	-4.918215000000	0.150626000000
H	10.706722000000	-2.503855000000	0.101037000000

9c, PCM_{CH2Cl2}/DFT-B3LYP/6-31G(d,p) optimization; final energy -1924.99682222 Hartree

C	-7.286005000000	-0.124291000000	-0.014102000000
C	-7.380025000000	1.256016000000	-0.039161000000
N	-6.083991000000	1.817609000000	-0.033660000000
C	-5.130072000000	0.859310000000	-0.006237000000
C	-5.811269000000	-0.516456000000	0.010566000000
C	-8.464934000000	-0.917215000000	-0.014057000000
C	-9.728736000000	-0.224115000000	-0.041237000000
C	-9.759795000000	1.197764000000	-0.066151000000
C	-8.608171000000	1.949256000000	-0.065388000000
C	-5.424759000000	-1.321354000000	-1.257581000000
C	-5.455894000000	-1.272976000000	1.317006000000
C	-5.807549000000	3.251587000000	-0.055327000000
C	-3.767991000000	1.179274000000	0.002908000000
C	-2.698931000000	0.285857000000	0.025985000000
C	-1.355233000000	0.683325000000	0.028908000000
C	-0.310092000000	-0.232477000000	0.044922000000
C	1.051401000000	0.098679000000	0.042467000000
C	1.624259000000	1.505684000000	0.025745000000
C	3.167120000000	1.317071000000	0.027614000000
C	3.388602000000	-0.187616000000	0.040914000000
C	2.113279000000	-0.802268000000	0.050962000000
Cl	1.902240000000	-2.533844000000	0.069875000000
C	4.604241000000	-0.840216000000	0.040931000000
C	5.867290000000	-0.205012000000	0.028493000000
C	7.044180000000	-0.918031000000	0.025215000000
C	8.384487000000	-0.410256000000	0.012457000000
C	9.472639000000	-1.317790000000	0.009322000000
C	10.786257000000	-0.895445000000	-0.002892000000
C	11.104511000000	0.491604000000	-0.012829000000
C	10.014782000000	1.411683000000	-0.009424000000
C	8.709326000000	0.970541000000	0.002576000000
N	12.398502000000	0.925841000000	-0.025100000000
C	12.700497000000	2.353857000000	-0.033218000000
C	13.497848000000	-0.034116000000	-0.028518000000
C	-8.484646000000	-2.341760000000	0.011022000000
C	-9.674073000000	-3.036201000000	0.008845000000
C	-10.912275000000	-2.351842000000	-0.018241000000
C	-10.933517000000	-0.975889000000	-0.042563000000
H	-10.725119000000	1.694540000000	-0.086159000000
H	-8.657996000000	3.031179000000	-0.084222000000
H	-5.983446000000	-2.258027000000	-1.298363000000
H	-4.360497000000	-1.563856000000	-1.267425000000
H	-5.654557000000	-0.751054000000	-2.161375000000
H	-4.390098000000	-1.503219000000	1.366822000000
H	-5.718147000000	-0.674474000000	2.193316000000
H	-6.006772000000	-2.213466000000	1.373758000000
H	-6.744063000000	3.801639000000	-0.080689000000
H	-5.253347000000	3.542974000000	0.840497000000
H	-5.224826000000	3.510233000000	-0.942996000000
H	-3.512292000000	2.234200000000	-0.011756000000
H	-2.888232000000	-0.783165000000	0.040844000000
H	-1.133415000000	1.748198000000	0.015052000000
H	-0.564459000000	-1.290369000000	0.058026000000
H	1.284622000000	2.053664000000	-0.859885000000
H	1.284810000000	2.074896000000	0.897918000000
H	3.628298000000	1.786559000000	0.902837000000
H	3.628920000000	1.771645000000	-0.855066000000

H	4.593345000000	-1.928210000000	0.050276000000
H	5.892822000000	0.881413000000	0.020620000000
H	6.958228000000	-2.004767000000	0.032658000000
H	9.265073000000	-2.384637000000	0.016823000000
H	11.575223000000	-1.636271000000	-0.004548000000
H	10.204682000000	2.477127000000	-0.016360000000
H	7.916854000000	1.712102000000	0.004628000000
H	12.301583000000	2.854934000000	0.856624000000
H	13.780844000000	2.488336000000	-0.041672000000
H	12.288618000000	2.847603000000	-0.921251000000
H	13.462874000000	-0.681705000000	-0.912514000000
H	14.442765000000	0.506666000000	-0.041363000000
H	13.479790000000	-0.669295000000	0.865009000000
H	-7.553931000000	-2.894700000000	0.032512000000
H	-9.661339000000	-4.121633000000	0.028210000000
H	-11.840528000000	-2.914291000000	-0.019616000000
H	-11.878509000000	-0.440292000000	-0.063266000000

9d, PCM_{CH2Cl2}/DFT-B3LYP/6-31G(d,p) optimization; final energy -1924.99671895 Hartree

C	6.974725000000	-1.485995000000	0.001609000000
C	7.277939000000	-0.136384000000	-0.041221000000
N	6.083361000000	0.615378000000	-0.072325000000
C	4.993300000000	-0.186372000000	-0.039552000000
C	5.457104000000	-1.649593000000	0.004625000000
C	8.019483000000	-2.448272000000	0.034917000000
C	9.374290000000	-1.955574000000	0.024523000000
C	9.621458000000	-0.555415000000	-0.015757000000
C	8.597614000000	0.361899000000	-0.048294000000
C	4.973325000000	-2.406535000000	-1.260067000000
C	4.969735000000	-2.325311000000	1.312662000000
C	6.028561000000	2.073160000000	-0.130296000000
C	3.696601000000	0.337776000000	-0.039384000000
C	2.507085000000	-0.391499000000	-0.028587000000
C	1.237542000000	0.189542000000	-0.022286000000
C	0.932397000000	1.677557000000	-0.015694000000
C	-0.619079000000	1.774710000000	0.000710000000
C	-1.111650000000	0.336225000000	-0.014494000000
C	0.022015000000	-0.501353000000	-0.023023000000
Cl	-0.086665000000	-2.242035000000	-0.035081000000
C	-2.432125000000	-0.082387000000	-0.020158000000
C	-3.552122000000	0.768155000000	-0.008652000000
C	-4.850655000000	0.289271000000	-0.020650000000
C	-6.010000000000	1.095249000000	-0.007924000000
C	-7.276929000000	0.557899000000	-0.026105000000
C	-8.531368000000	1.248882000000	-0.013587000000
C	-9.735873000000	0.503401000000	-0.055013000000
C	-10.976884000000	1.105672000000	-0.044841000000
C	-11.097472000000	2.522867000000	0.010480000000
C	-9.889408000000	3.280459000000	0.052698000000
C	-8.658830000000	2.660815000000	0.040341000000
N	-12.317552000000	3.133572000000	0.023581000000
C	-12.416959000000	4.588750000000	0.084899000000
C	-13.540856000000	2.337964000000	-0.014822000000
C	7.822279000000	-3.858796000000	0.077839000000
C	8.892209000000	-4.725595000000	0.107261000000
C	10.220347000000	-4.237568000000	0.096387000000
C	10.450767000000	-2.881219000000	0.056256000000
H	10.651266000000	-0.211278000000	-0.019458000000
H	8.811851000000	1.423310000000	-0.075048000000
H	5.389874000000	-3.415127000000	-1.281237000000
H	5.367269000000	-3.338968000000	1.388695000000
H	3.885347000000	-2.492021000000	-1.283159000000
H	3.880448000000	-2.386647000000	1.346550000000
H	5.296000000000	-1.888990000000	-2.167156000000
H	5.308008000000	-1.762986000000	2.186895000000
H	7.033549000000	2.471318000000	-0.239786000000
H	5.433136000000	2.391284000000	-0.989454000000
H	5.586668000000	2.474810000000	0.785625000000
H	3.609969000000	1.418707000000	-0.046863000000
H	2.540592000000	-1.474468000000	-0.027865000000
H	1.358062000000	2.166487000000	-0.898985000000
H	1.377385000000	2.163945000000	0.859192000000

H	-0.997595000000	2.326599000000	-0.865920000000
H	-0.979159000000	2.299838000000	0.891705000000
H	-3.396629000000	1.844799000000	0.010417000000
H	-4.994087000000	-0.791356000000	-0.041944000000
H	-2.619016000000	-1.154265000000	-0.035154000000
H	-5.872491000000	2.174074000000	0.015670000000
H	-7.345982000000	-0.529491000000	-0.054443000000
H	-9.679538000000	-0.581230000000	-0.097744000000
H	-11.861703000000	0.483702000000	-0.079161000000
H	-9.928380000000	4.361136000000	0.096713000000
H	-7.769705000000	3.282190000000	0.076132000000
H	-11.958790000000	4.985453000000	0.998581000000
H	-11.933122000000	5.061455000000	-0.777758000000
H	-13.467738000000	4.873623000000	0.081492000000
H	-13.598536000000	1.733865000000	-0.927881000000
H	-14.400480000000	3.005792000000	0.004653000000
H	-13.610793000000	1.665994000000	0.848707000000
H	6.818159000000	-4.263605000000	0.087507000000
H	8.714166000000	-5.796127000000	0.139209000000
H	11.052245000000	-4.934319000000	0.119934000000
H	11.466390000000	-2.495326000000	0.047952000000

^{1}H NMR spectra of the synthesized compounds

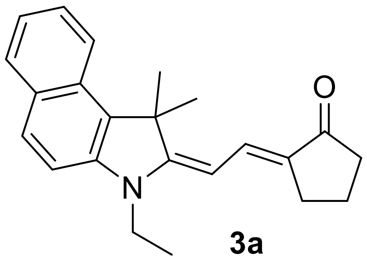

3a

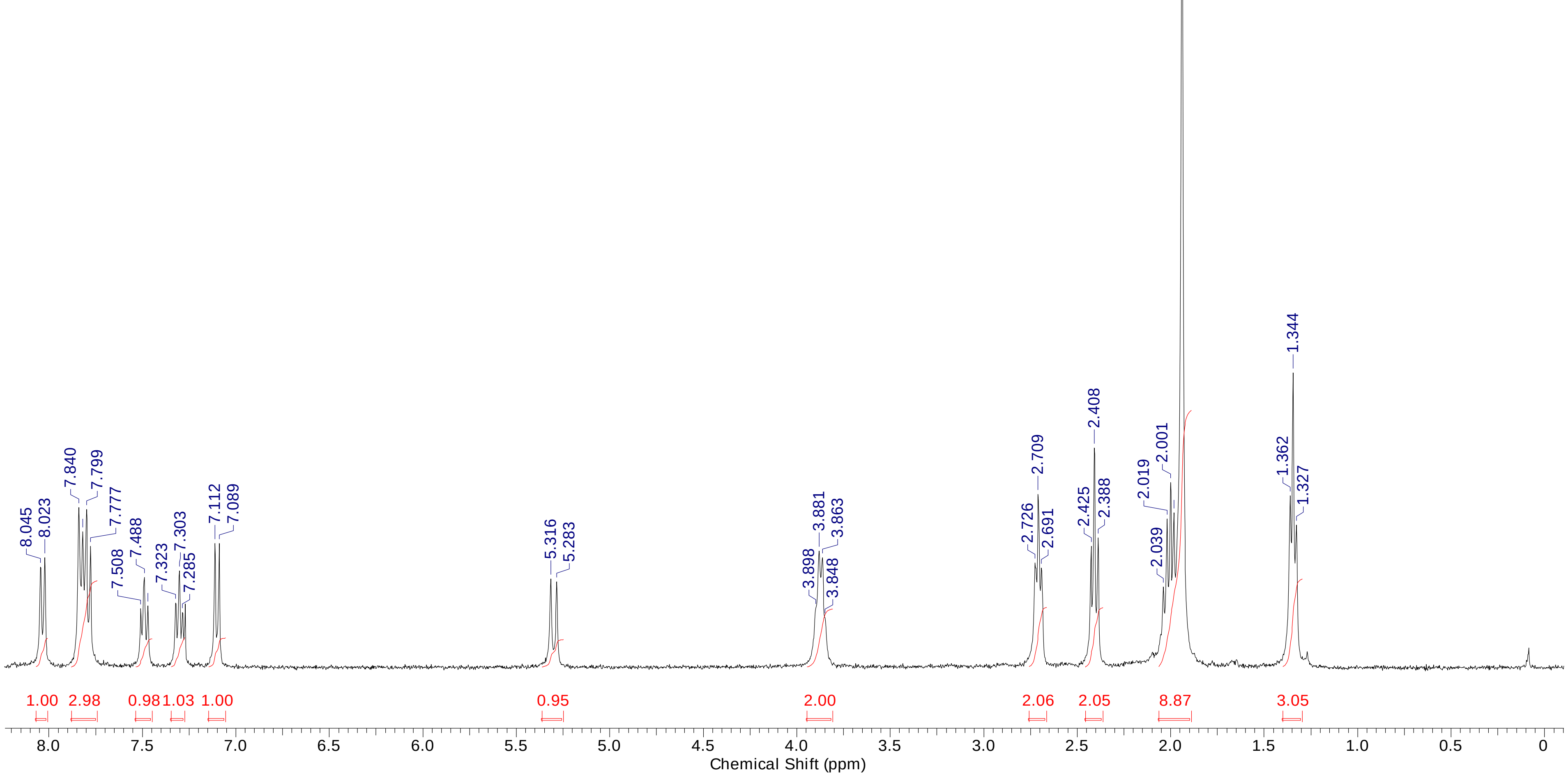

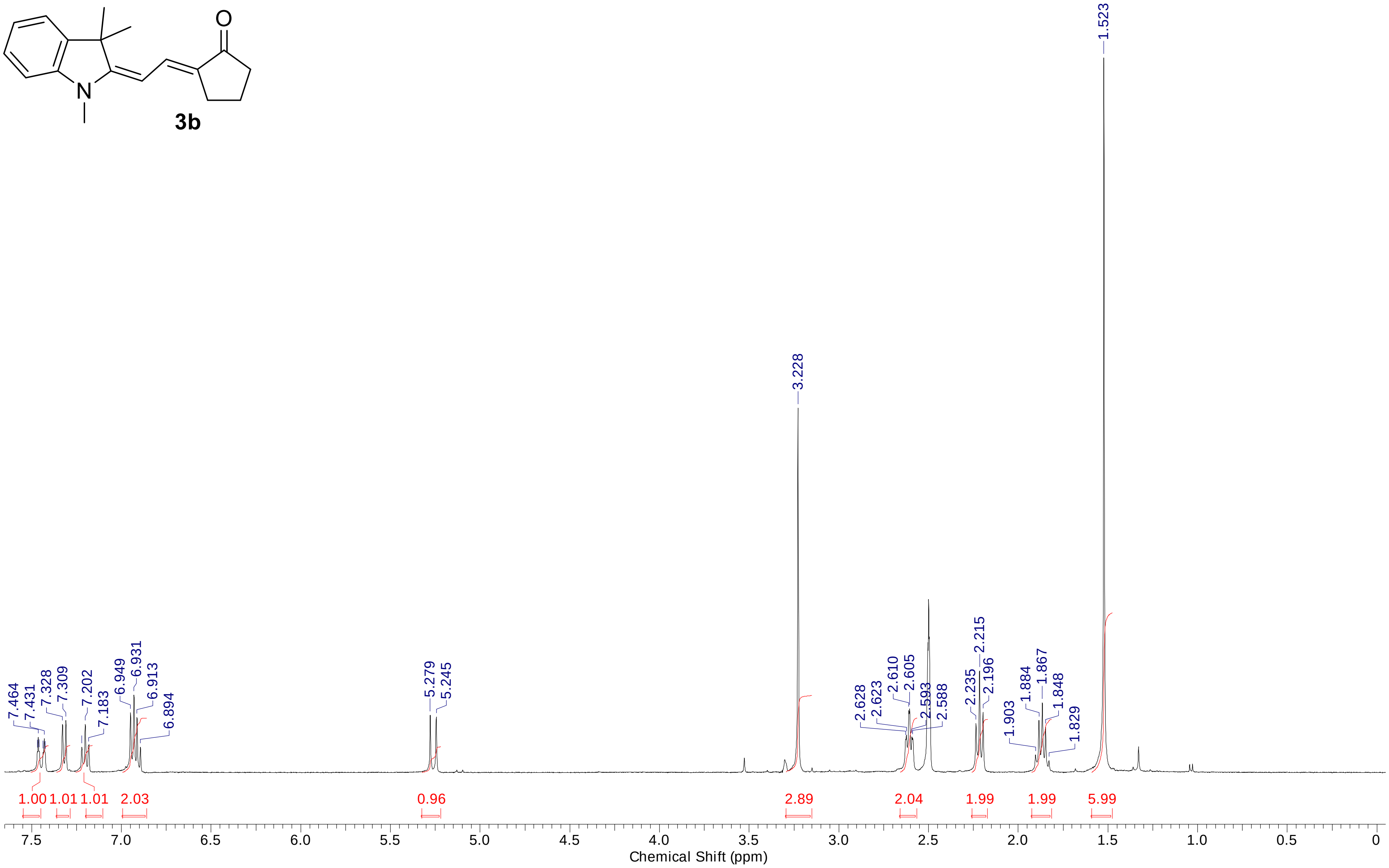
3b
7.464
7.431
7.328
7.309
7.202
7.183
6.949
6.931
6.913
6.894
5.279
5.245
3.228
2.628
2.623
2.610
2.605
2.593
2.588
2.235
2.215
2.196
1.903
1.884
1.867
1.848
1.829
1.523
1.00
1.01
1.01
2.03
0.96
2.89
2.04
1.99
1.99
5.99
Chemical Shift (ppm)

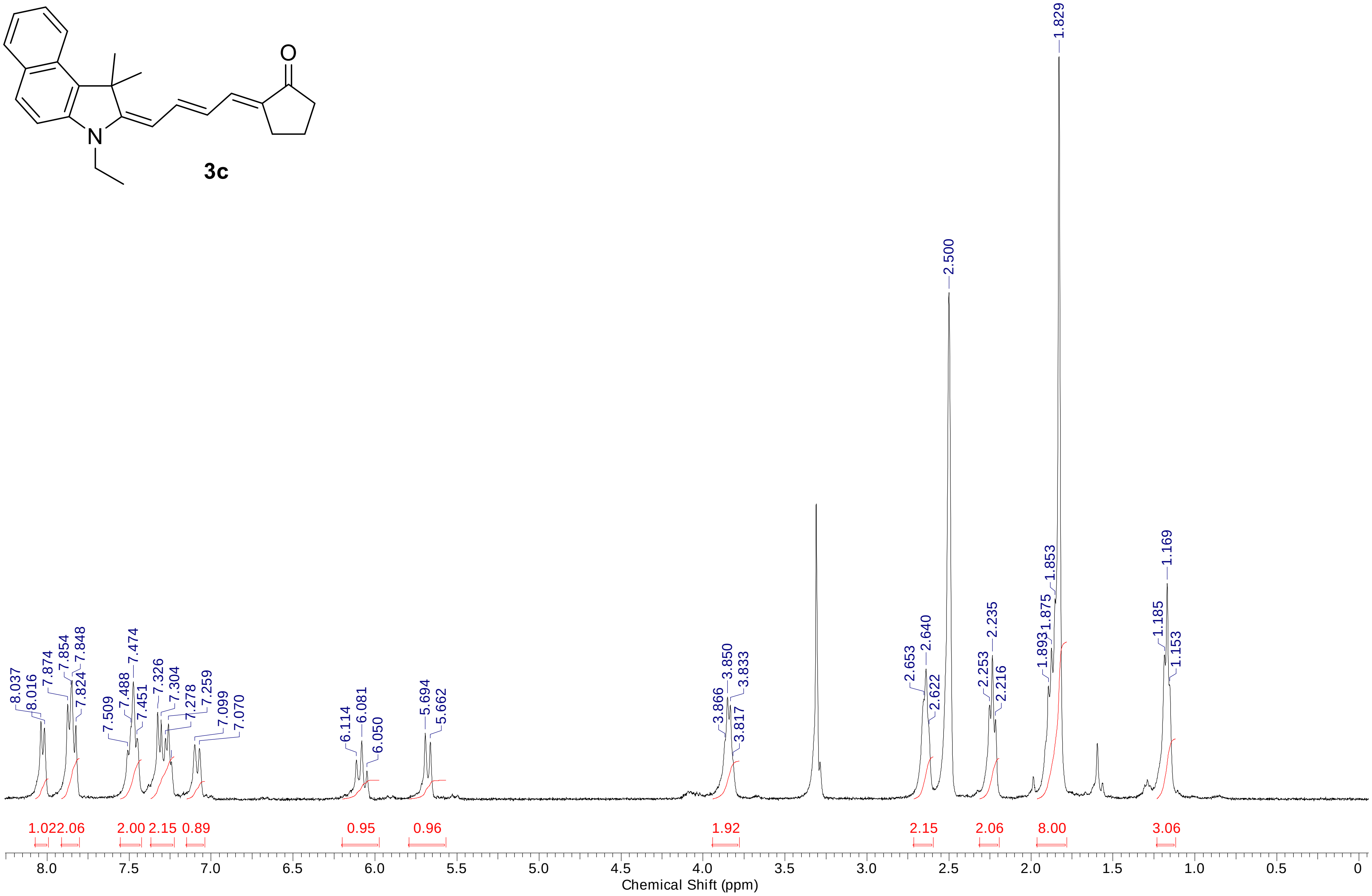
3c
Chemical Shift (ppm)
1.02 2.06 2.00 2.15 0.89 0.95 0.96 1.92 2.15 2.06 8.00 3.06
8.037 8.016 7.874 7.854 7.848 7.824 7.509 7.488 7.474 7.451 7.326 7.304 7.278 7.259 7.099 7.070
6.114 6.081 6.050 5.694 5.662
3.866 3.850 3.833 3.817 2.653 2.640 2.622 2.500 2.253 2.235 2.216 1.893 1.875 1.853 1.829 1.185 1.169 1.153

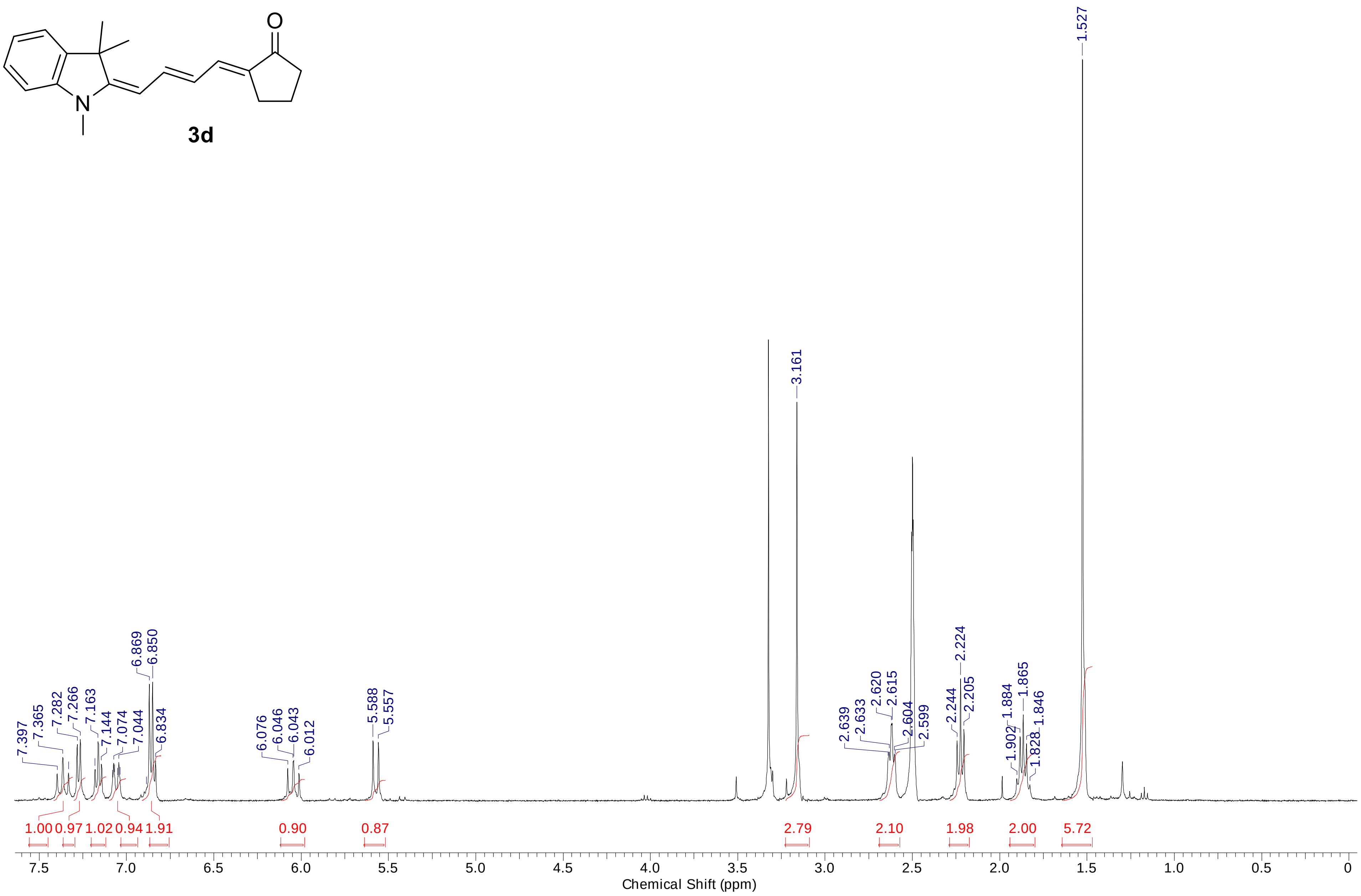
3d
7.397
7.365
7.282
7.266
7.163
7.144
7.074
7.044
6.869
6.850
6.834
6.076
6.046
6.043
6.012
5.588
5.557
3.161
2.639
2.633
2.620
2.615
2.604
2.599
2.244
2.224
2.205
1.902
1.884
1.865
1.846
1.828
1.527
1.00
0.97
1.02
0.94
1.91
0.90
0.87
2.79
2.10
1.98
2.00
5.72
Chemical Shift (ppm)

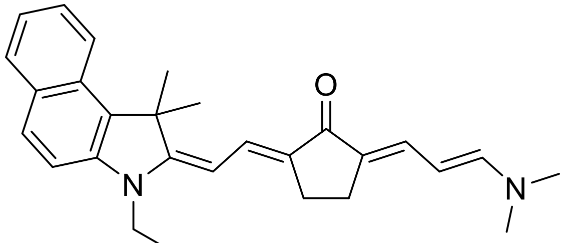
O
N
N

4a

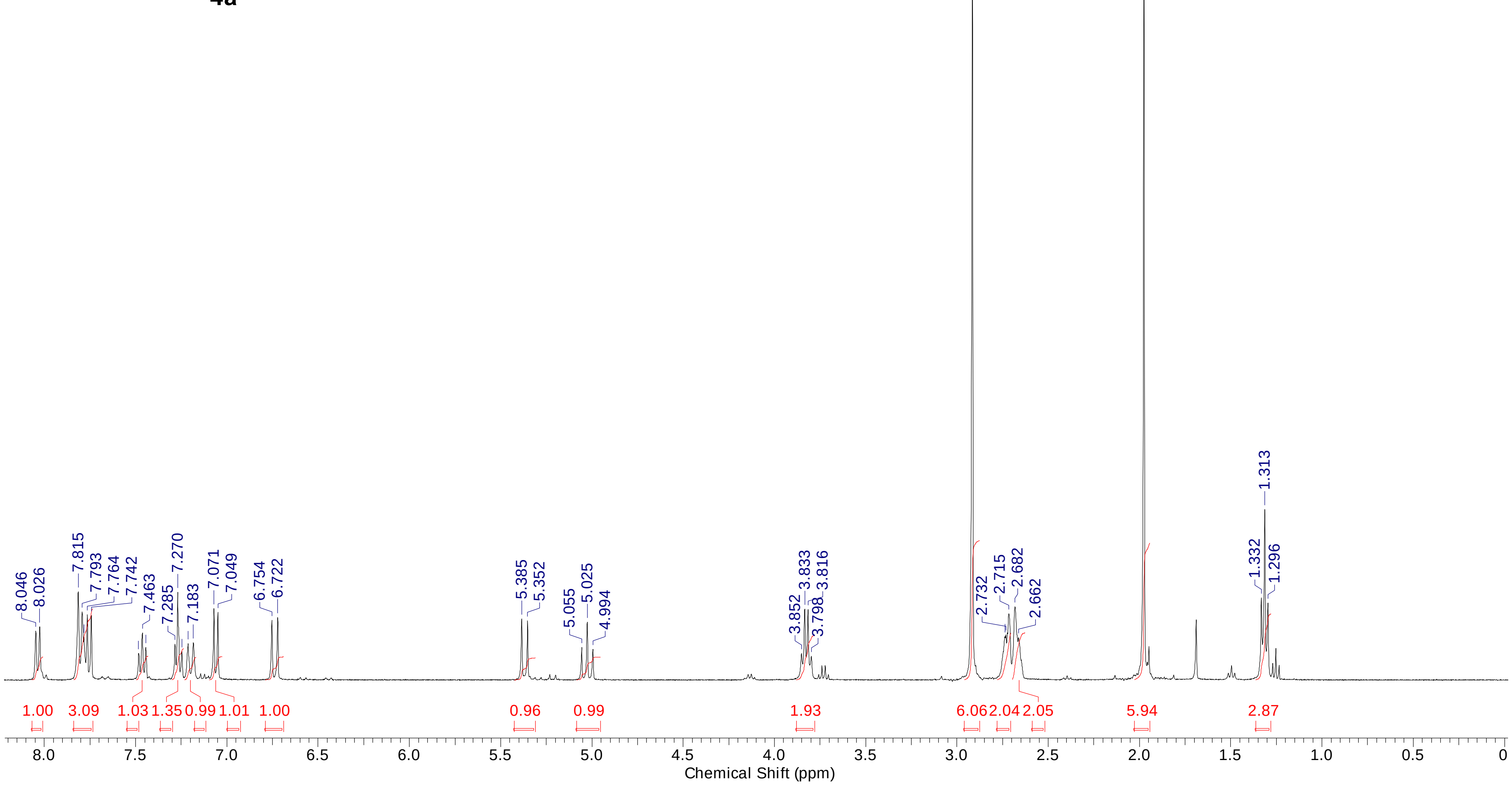
8.046
8.026
7.815
7.793
7.764
7.742
7.463
7.285
7.270
7.183
7.071
7.049
6.754
6.722
5.385
5.352
5.055
5.025
4.994
3.852
3.833
3.816
3.798
2.915
2.732
2.715
2.682
2.662
1.975
1.332
1.313
1.296
1.00
3.09
1.03
1.35
0.99
1.01
1.00
0.96
0.99
1.93
6.06
2.04
2.05
5.94
2.87
8.0
7.5
7.0
6.5
6.0
5.5
5.0
4.5
4.0
3.5
3.0
2.5
2.0
1.5
1.0
0.5
0
Chemical Shift (ppm)

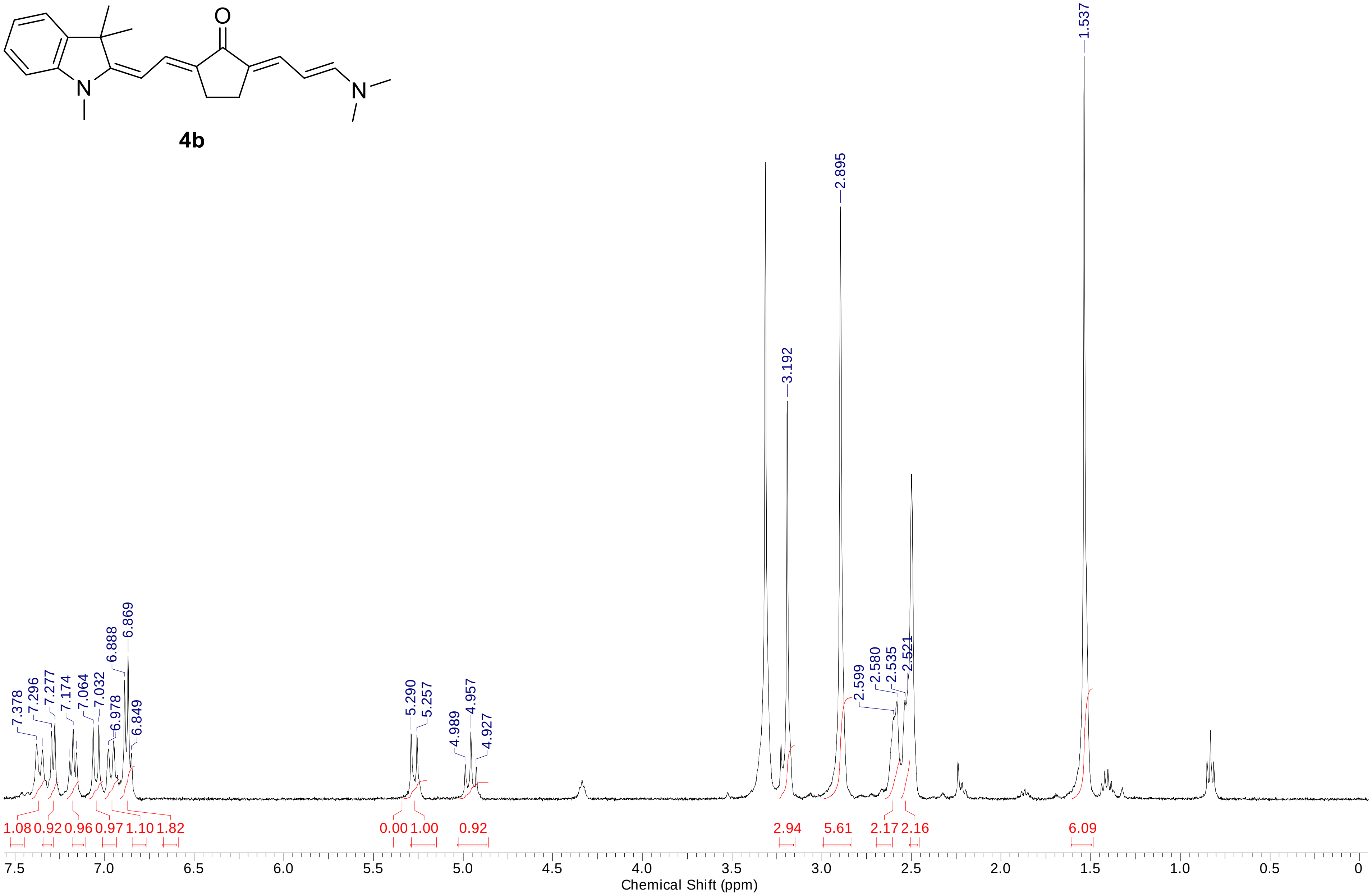
4b
O
N
7.378
7.296
7.277
7.174
7.064
7.032
6.978
6.888
6.869
6.849
5.290
5.257
4.989
4.957
4.927
3.192
2.895
2.599
2.580
2.535
2.521
1.537
1.08 0.92 0.96 0.97 1.10 1.82
0.00 1.00
0.92
2.94
5.61
2.17 2.16
6.09
7.5 7.0 6.5 6.0 5.5 5.0 4.5 4.0 3.5 3.0 2.5 2.0 1.5 1.0 0.5 0
Chemical Shift (ppm)

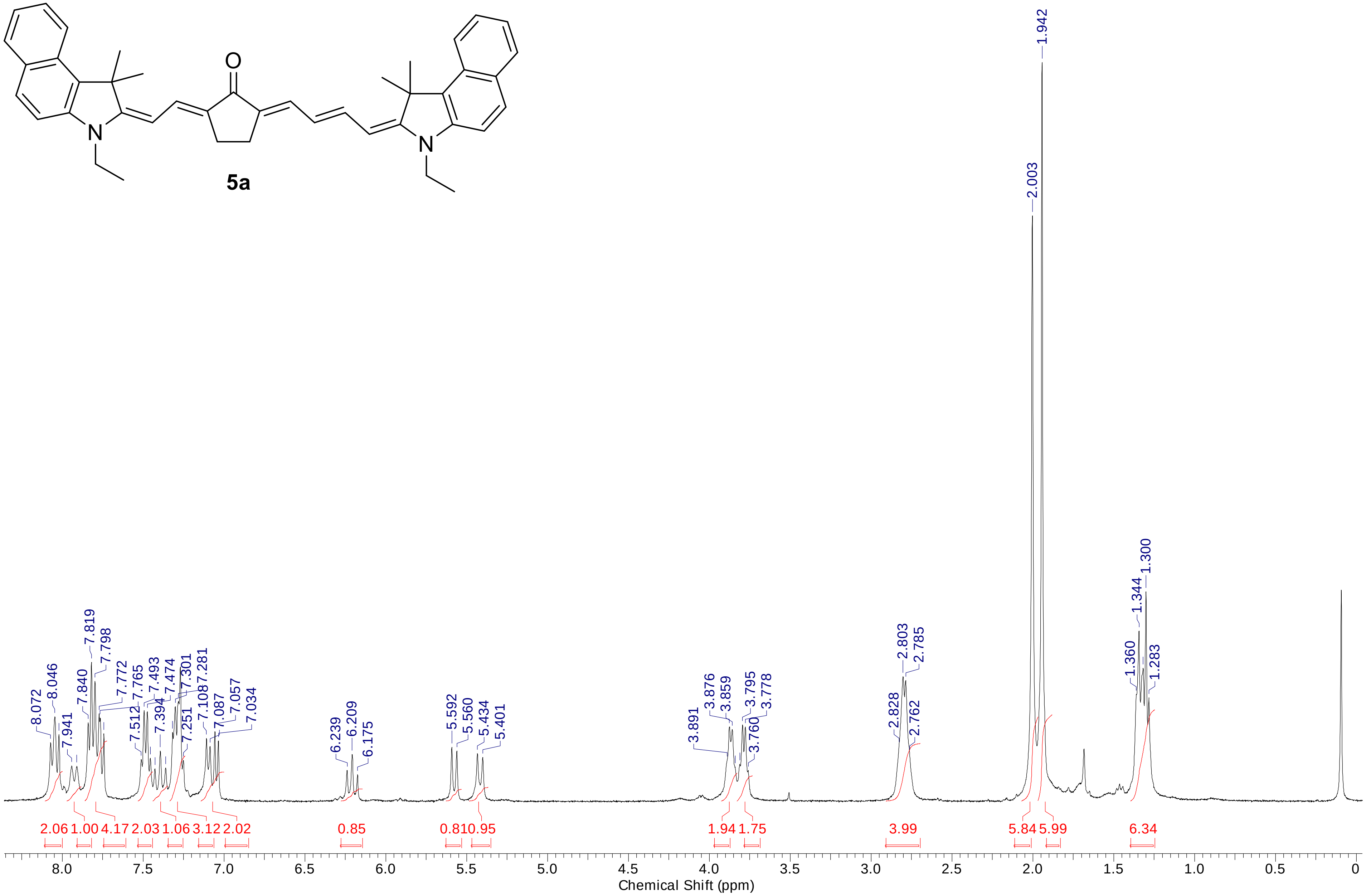
O
N
N
5a
Chemical Shift (ppm)
2.06 1.00 4.17 2.03 1.06 3.12 2.02
0.85
0.81 0.95
1.94 1.75
3.99
5.84 5.99
6.34
8.072 8.046 7.941 7.840 7.819 7.798 7.772 7.765 7.512 7.493 7.474 7.394 7.301 7.281 7.251 7.108 7.087 7.057 7.034
6.239 6.209 6.175
5.592 5.560 5.434 5.401
3.891 3.876 3.859 3.795 3.778 3.760
2.828 2.803 2.785 2.762
2.003 1.942
1.360 1.344 1.300 1.283

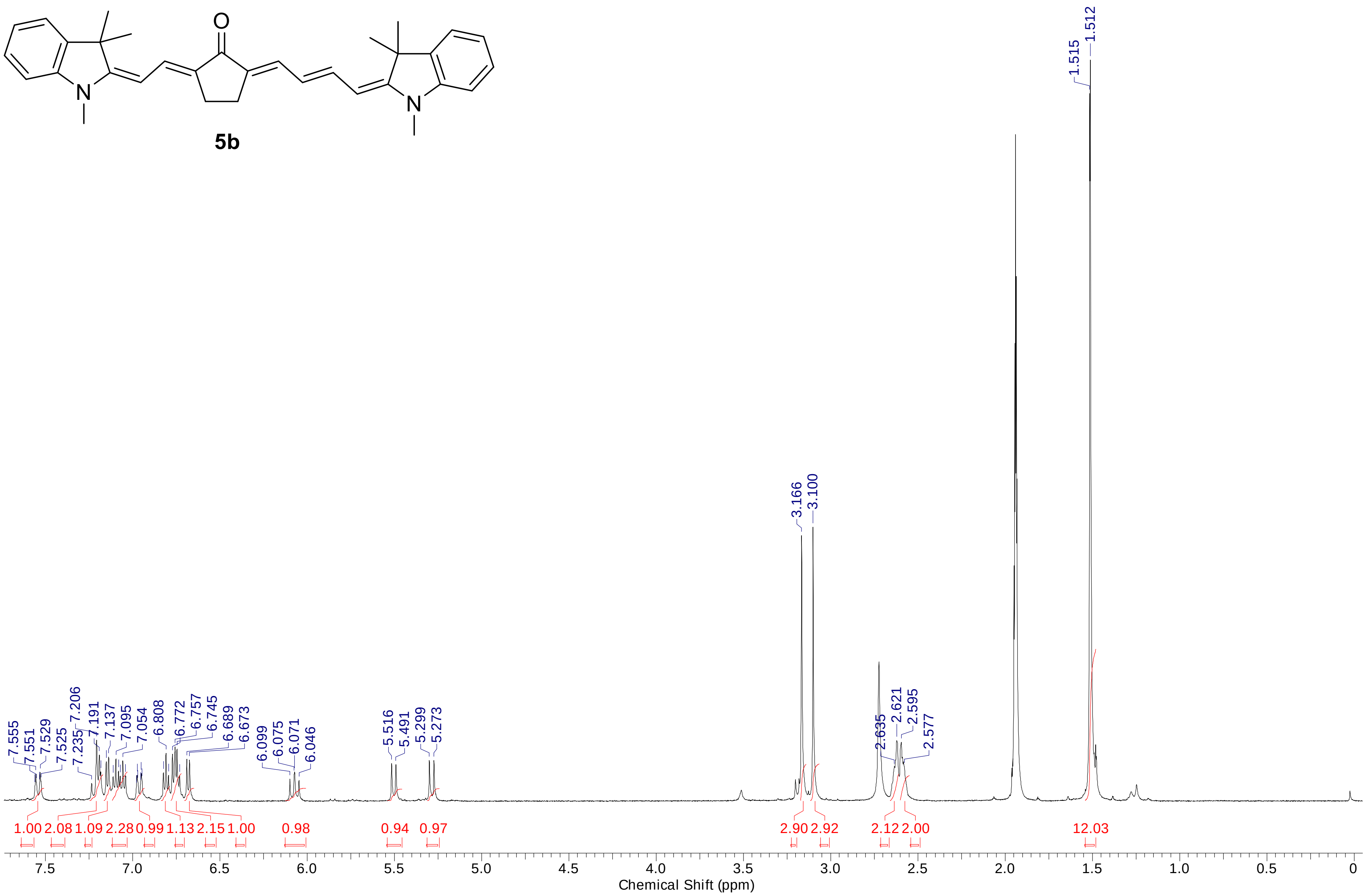
5b
7.555
7.551
7.529
7.525
7.235
7.206
7.191
7.137
7.095
7.054
6.808
6.772
6.757
6.745
6.689
6.673
6.099
6.075
6.071
6.046
5.516
5.491
5.299
5.273
3.166
3.100
2.635
2.621
2.595
2.577
1.515
1.512
1.00 2.08 1.09 2.28 0.99 1.13 2.15 1.00 0.98 0.94 0.97 2.90 2.92 2.12 2.00 12.03
7.5 7.0 6.5 6.0 5.5 5.0 4.5 4.0 3.5 3.0 2.5 2.0 1.5 1.0 0.5 0
Chemical Shift (ppm)

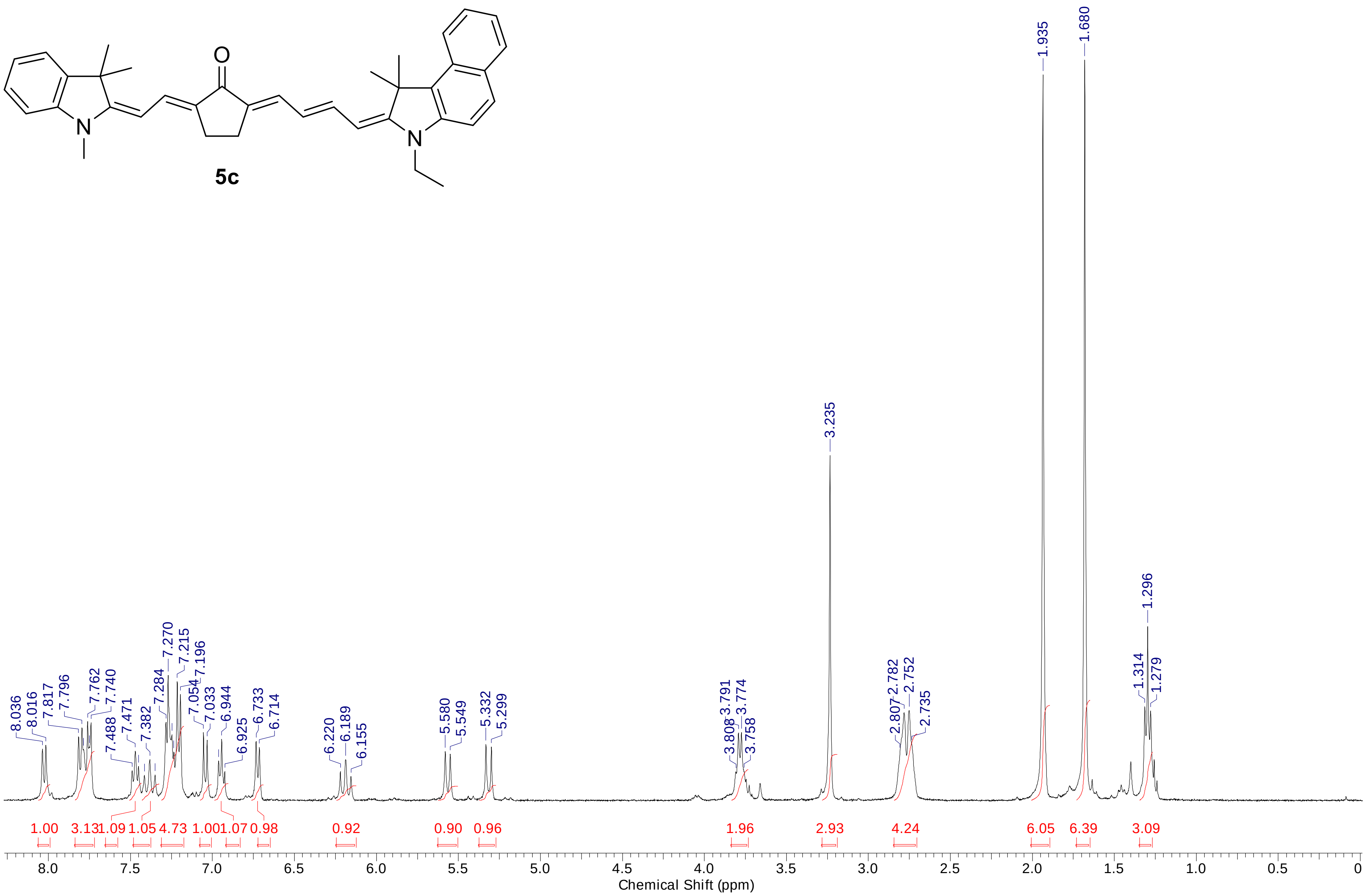
5c
O
N
N
8.036
8.016
7.817
7.796
7.762
7.740
7.488
7.471
7.382
7.284
7.270
7.215
7.196
7.054
7.033
6.944
6.925
6.733
6.714
6.220
6.189
6.155
5.580
5.549
5.332
5.299
3.808
3.791
3.774
3.758
3.235
2.807
2.782
2.752
2.735
1.935
1.680
1.314
1.296
1.279
1.00
3.13
1.09
1.05
4.73
1.00
1.07
0.98
0.92
0.90
0.96
1.96
2.93
4.24
6.05
6.39
3.09
8.0
7.5
7.0
6.5
6.0
5.5
5.0
4.5
4.0
3.5
3.0
2.5
2.0
1.5
1.0
0.5
0
Chemical Shift (ppm)

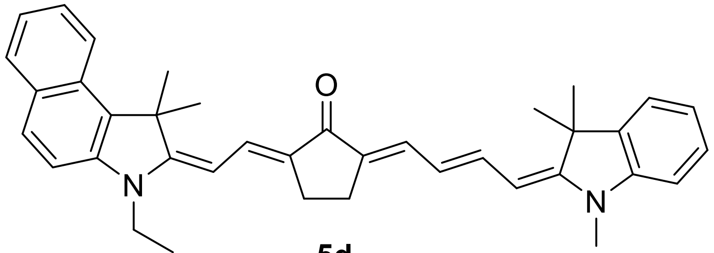

5d

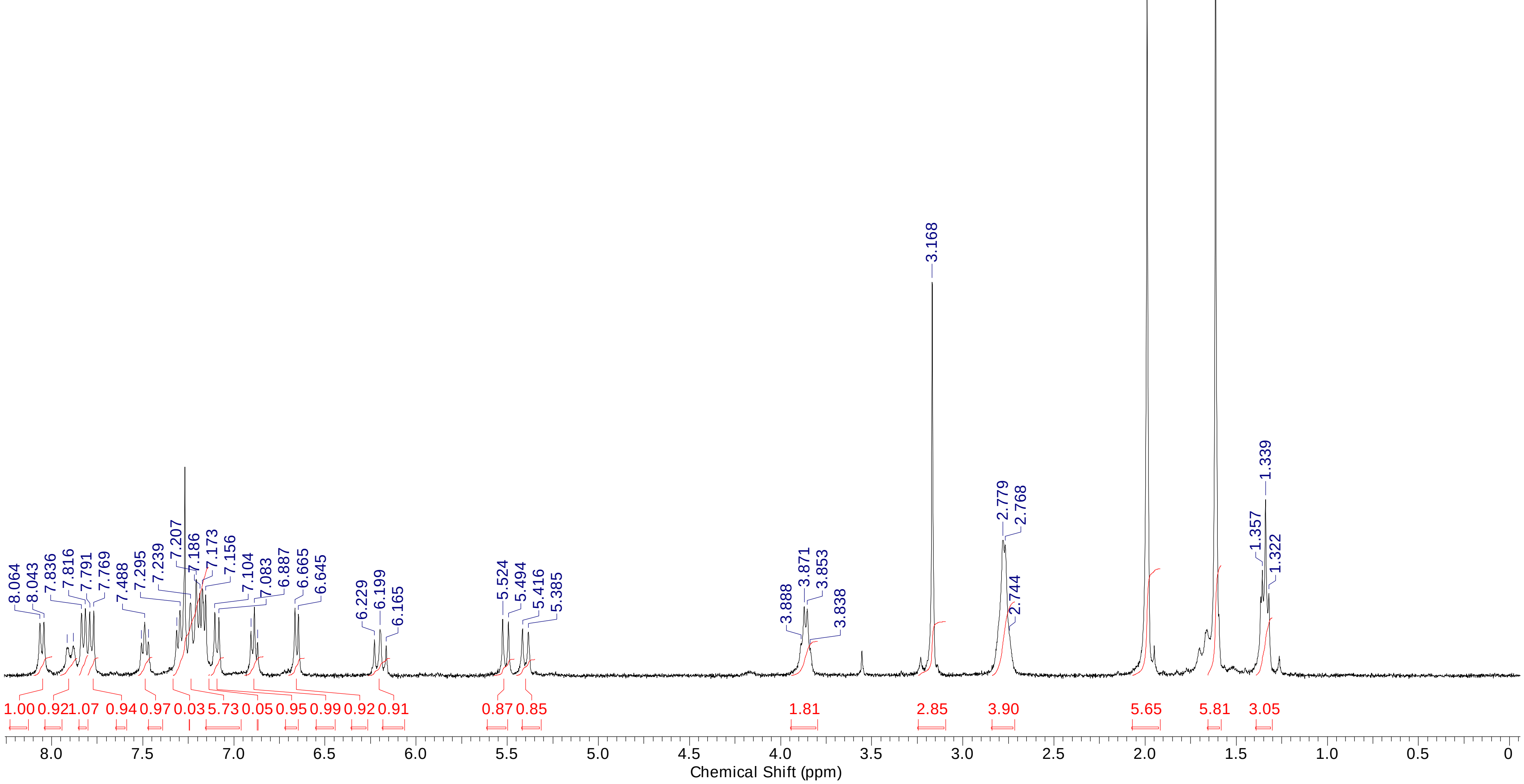

8.064
8.043
7.836
7.816
7.791
7.769
7.488
7.295
7.239
7.207
7.186
7.173
7.156
7.104
7.083
6.887
6.665
6.645
6.229
6.199
6.165
5.524
5.494
5.416
5.385
3.888
3.871
3.853
3.838
3.168
2.779
2.768
2.744
1.989
1.613
1.357
1.339
1.322
1.00
0.92
1.07
0.94
0.97
0.03
5.73
0.05
0.95
0.99
0.92
0.91
0.87
0.85
1.81
2.85
3.90
5.65
5.81
3.05
8.0
7.5
7.0
6.5
6.0
5.5
5.0
4.5
4.0
3.5
3.0
2.5
2.0
1.5
1.0
0.5
0
Chemical Shift (ppm)

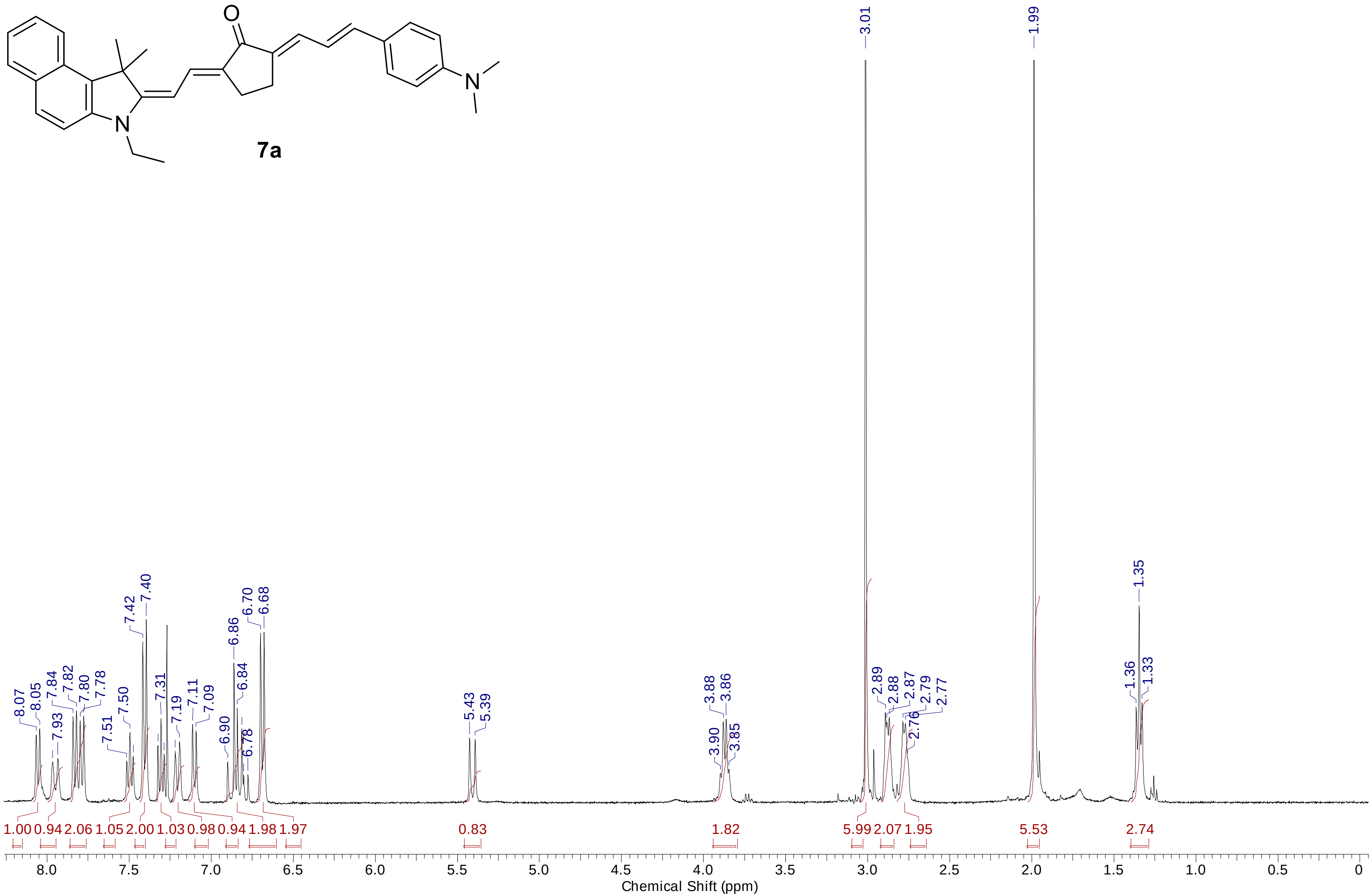
7a
8.07
8.05
7.93
7.84
7.82
7.80
7.78
7.51
7.50
7.42
7.40
7.31
7.19
7.11
7.09
6.90
6.86
6.84
6.78
6.70
6.68
5.43
5.39
3.90
3.88
3.86
3.85
3.01
2.89
2.88
2.87
2.79
2.77
2.76
1.99
1.36
1.35
1.33
1.00
0.94
2.06
1.05
2.00
1.03
0.98
0.94
1.98
1.97
0.83
1.82
5.99
2.07
1.95
5.53
2.74
8.0
7.5
7.0
6.5
6.0
5.5
5.0
4.5
4.0
3.5
3.0
2.5
2.0
1.5
1.0
0.5
0
Chemical Shift (ppm)

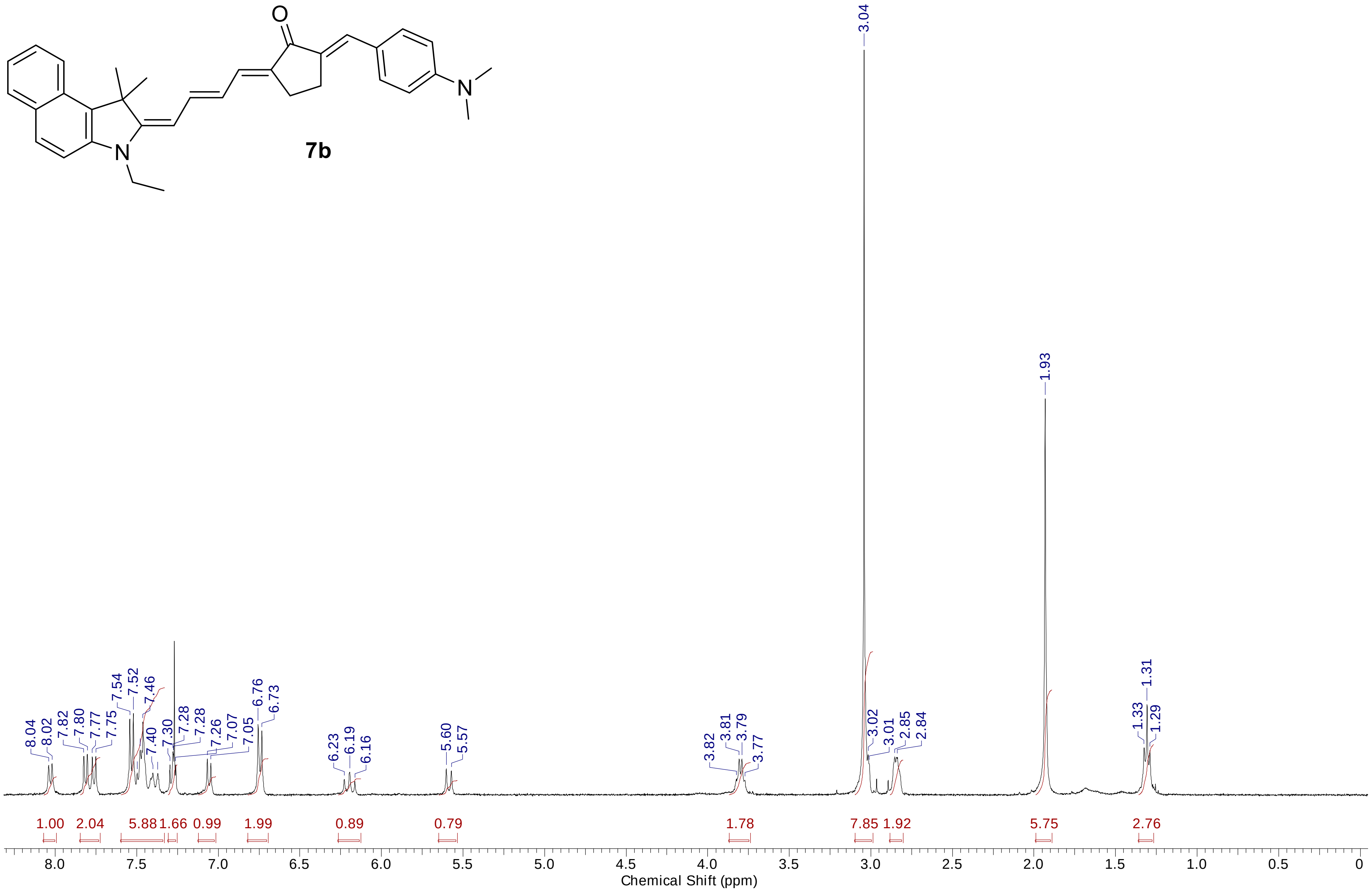

7b

8.04
8.02
7.82
7.80
7.77
7.75
7.54
7.52
7.46
7.40
7.30
7.28
7.28
7.26
7.07
7.05
6.76
6.73
6.23
6.19
6.16
5.60
5.57
3.82
3.81
3.79
3.77
3.04
3.02
3.01
2.85
2.84
1.93
1.33
1.31
1.29
1.00
2.04
5.88
1.66
0.99
1.99
0.89
0.79
1.78
7.85
1.92
5.75
2.76
8.0
7.5
7.0
6.5
6.0
5.5
5.0
4.5
4.0
3.5
3.0
2.5
2.0
1.5
1.0
0.5
0
Chemical Shift (ppm)

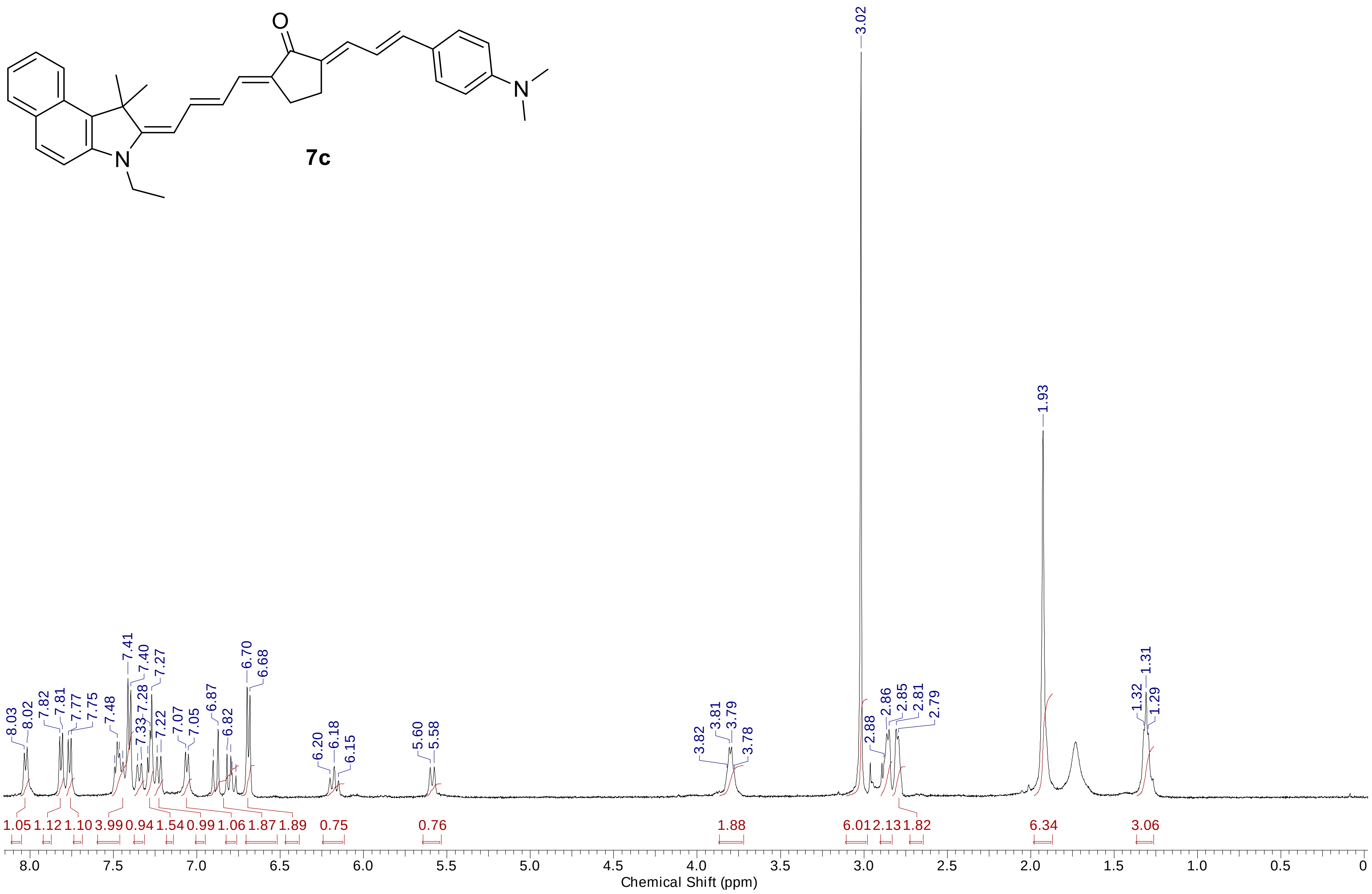
7c
Chemical Shift (ppm)

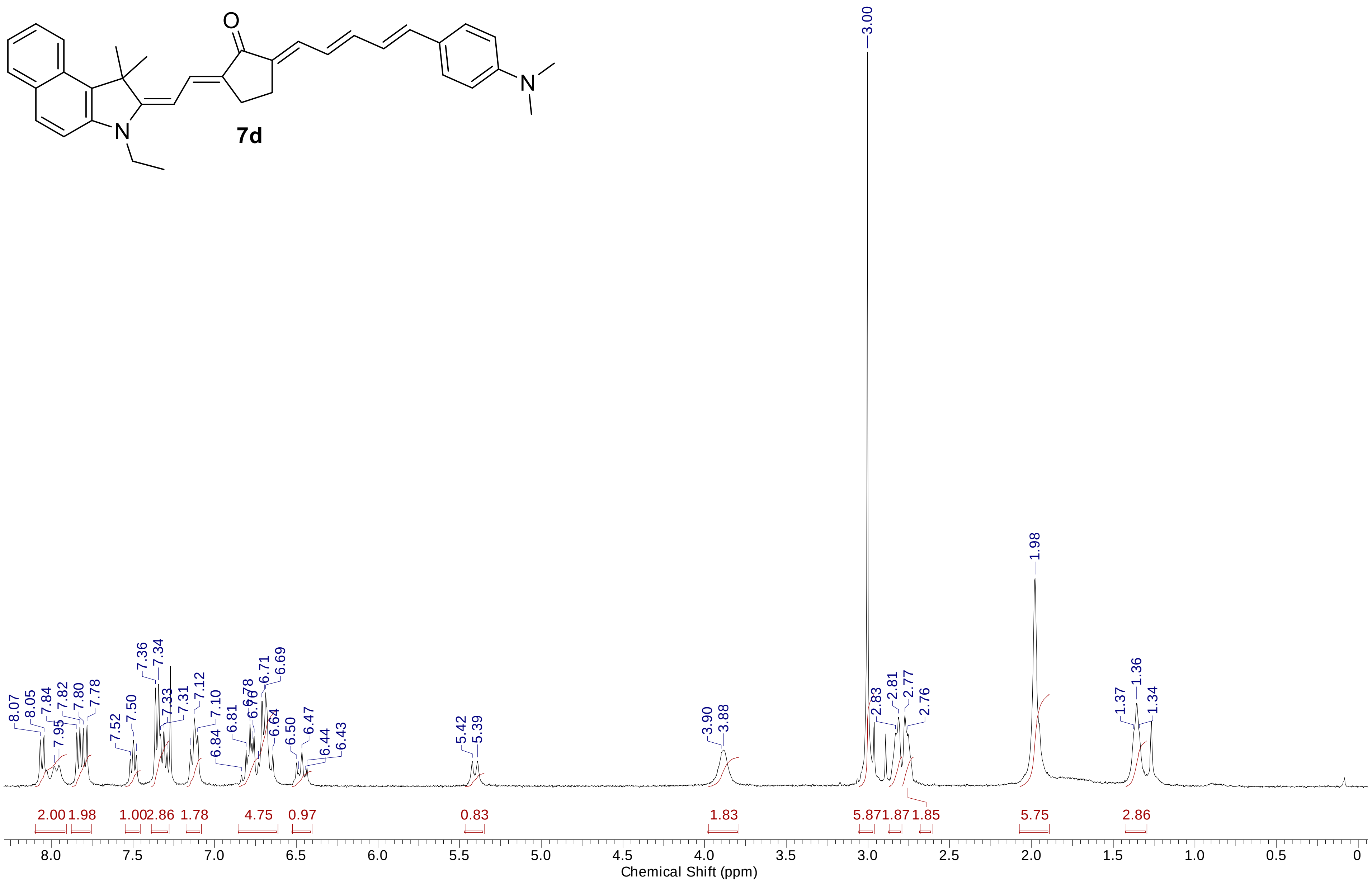
7d
O
N
8.07 8.05 7.95 7.84 7.82 7.80 7.78 7.52 7.50 7.36 7.34 7.33 7.31 7.12 7.10 6.84 6.81 6.78 6.76 6.71 6.69 6.64 6.50 6.47 6.44 6.43
5.42 5.39
3.90 3.88
3.00
2.83 2.81 2.77 2.76
1.98
1.37 1.36 1.34
2.00 1.98 1.00 2.86 1.78 4.75 0.97 0.83 1.83 5.87 1.87 1.85 5.75 2.86
8.0 7.5 7.0 6.5 6.0 5.5 5.0 4.5 4.0 3.5 3.0 2.5 2.0 1.5 1.0 0.5 0
Chemical Shift (ppm)

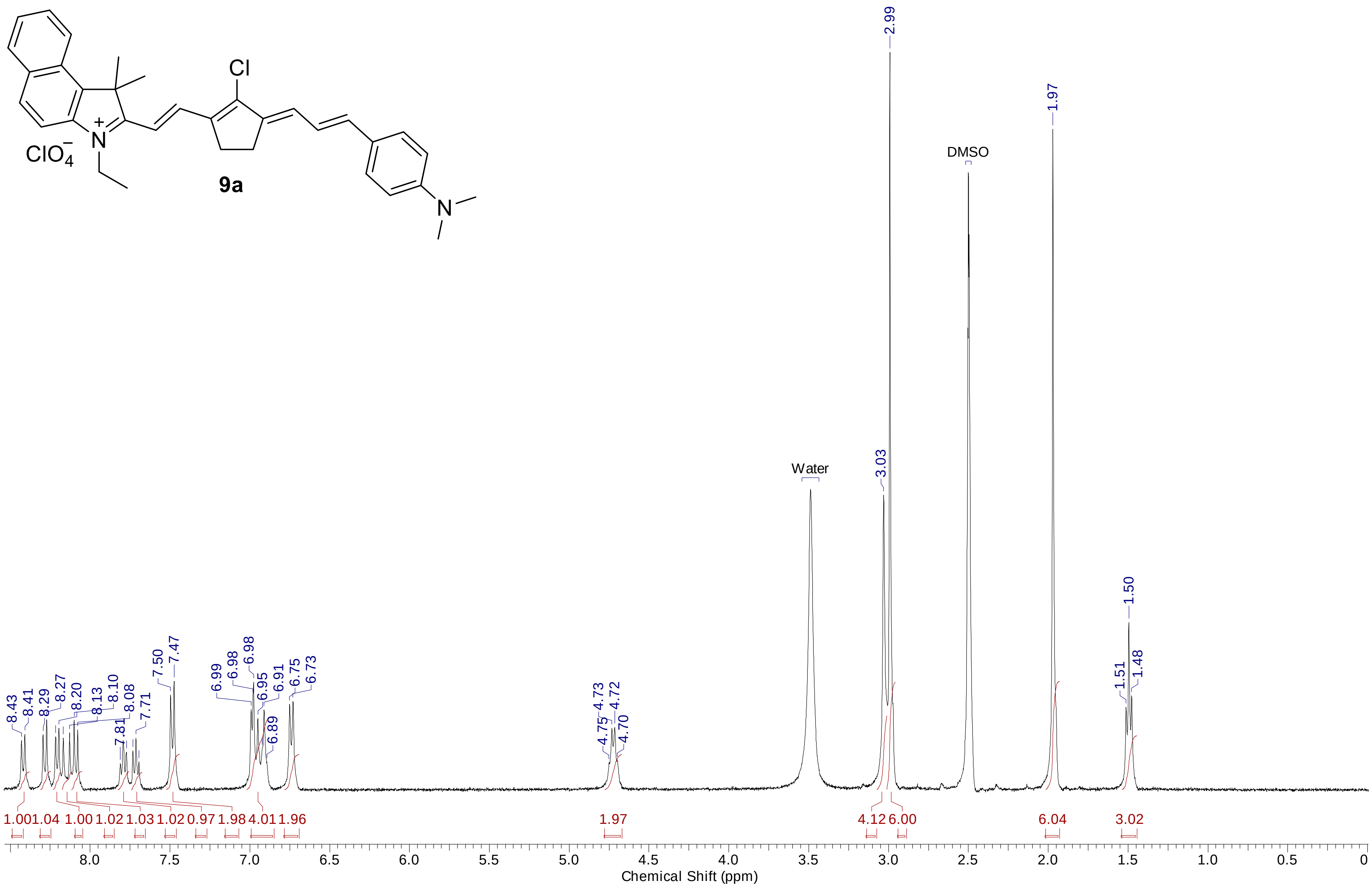
Cl
ClO_4^-
N
9a
Water
DMSO
2.99
3.03
1.97
1.50
1.51
1.48
4.73
4.72
4.75
4.70
8.43
8.41
8.29
8.27
8.20
8.13
8.10
8.08
7.81
7.71
7.50
7.47
6.99
6.98
6.98
6.95
6.91
6.89
6.75
6.73
1.00
1.04
1.00
1.02
1.03
1.02
0.97
1.98
4.01
1.96
1.97
4.12
6.00
6.04
3.02
8.0
7.5
7.0
6.5
6.0
5.5
5.0
4.5
4.0
3.5
3.0
2.5
2.0
1.5
1.0
0.5
0
Chemical Shift (ppm)

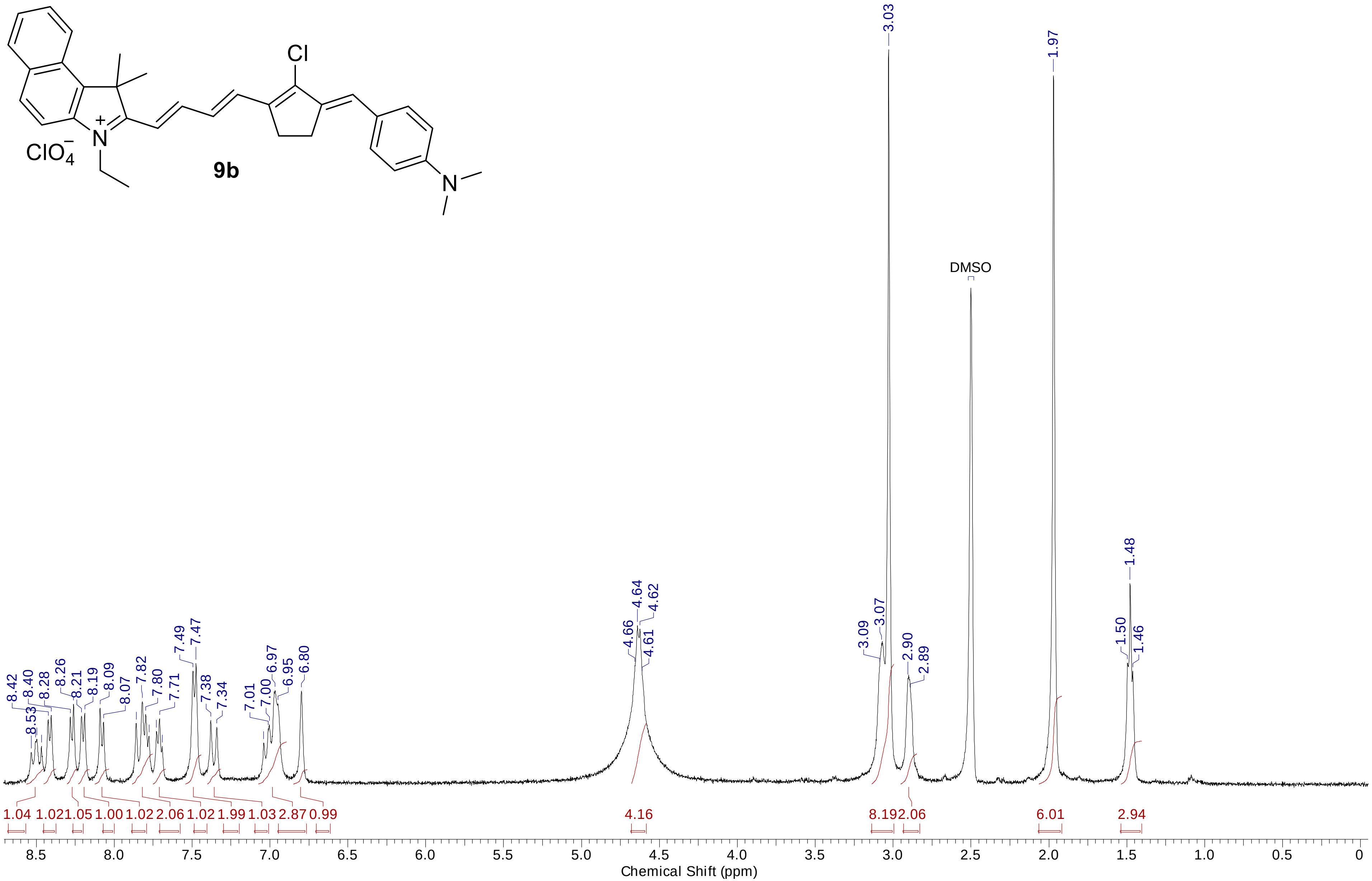
Cl
N
9b
DMSO
Chemical Shift (ppm)
1.04 1.02 1.05 1.00 1.02 2.06 1.02 1.99 1.03 2.87 0.99
4.16
8.19 2.06
6.01
2.94

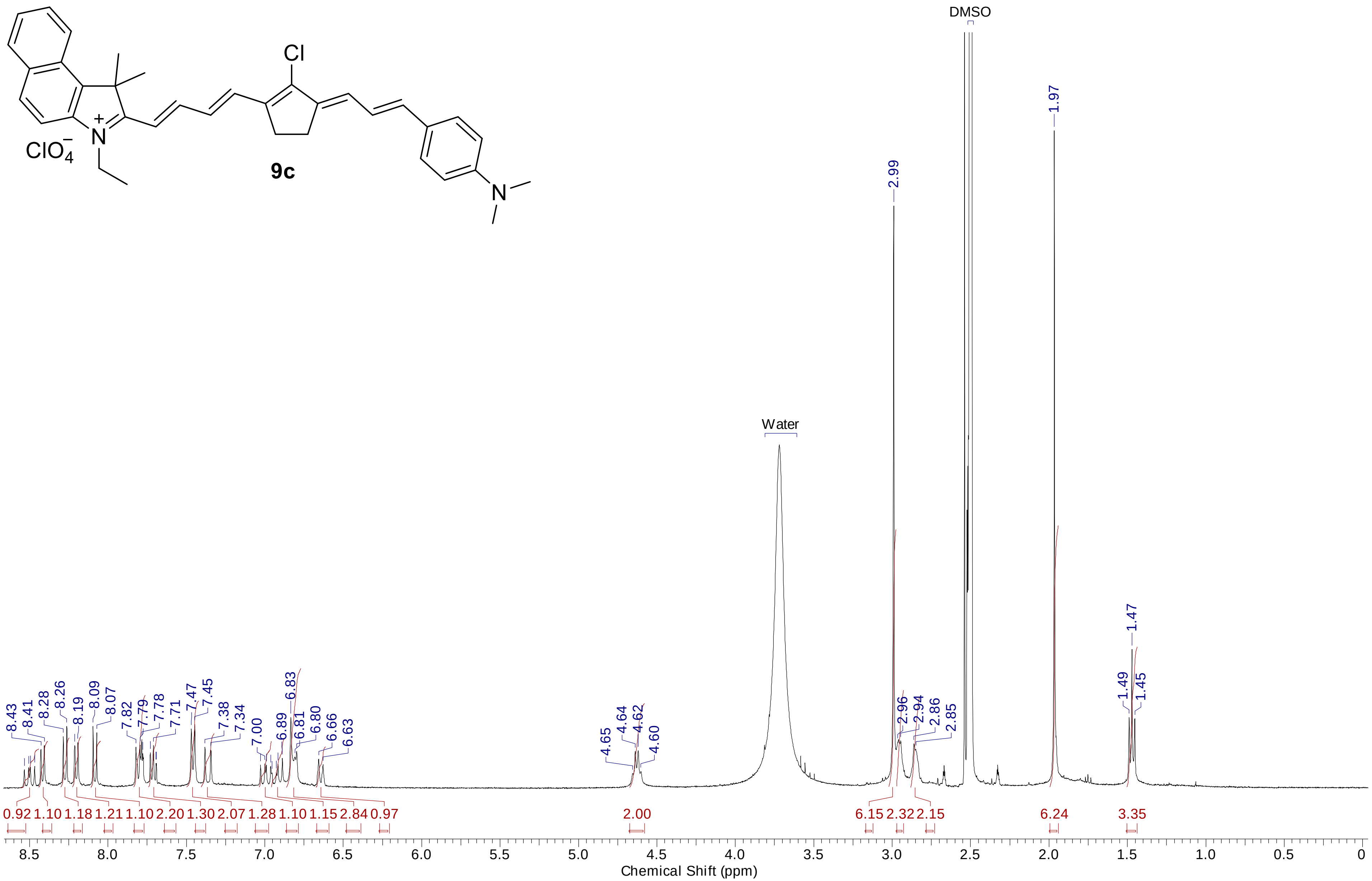

Cl
ClO4−
N+
N
9c
DMSO
Water
8.43 8.41 8.28 8.26 8.19 8.09 8.07 7.82 7.79 7.78 7.71 7.47 7.45 7.38 7.34 7.00 6.89 6.83 6.81 6.80 6.66 6.63
4.65 4.64 4.62 4.60
2.99 2.96 2.94 2.86 2.85
1.97
1.49 1.47 1.45
0.92 1.10 1.18 1.21 1.10 2.20 1.30 2.07 1.28 1.10 1.15 2.84 0.97
2.00
6.15 2.32 2.15
6.24
3.35
8.5 8.0 7.5 7.0 6.5 6.0 5.5 5.0 4.5 4.0 3.5 3.0 2.5 2.0 1.5 1.0 0.5 0
Chemical Shift (ppm)

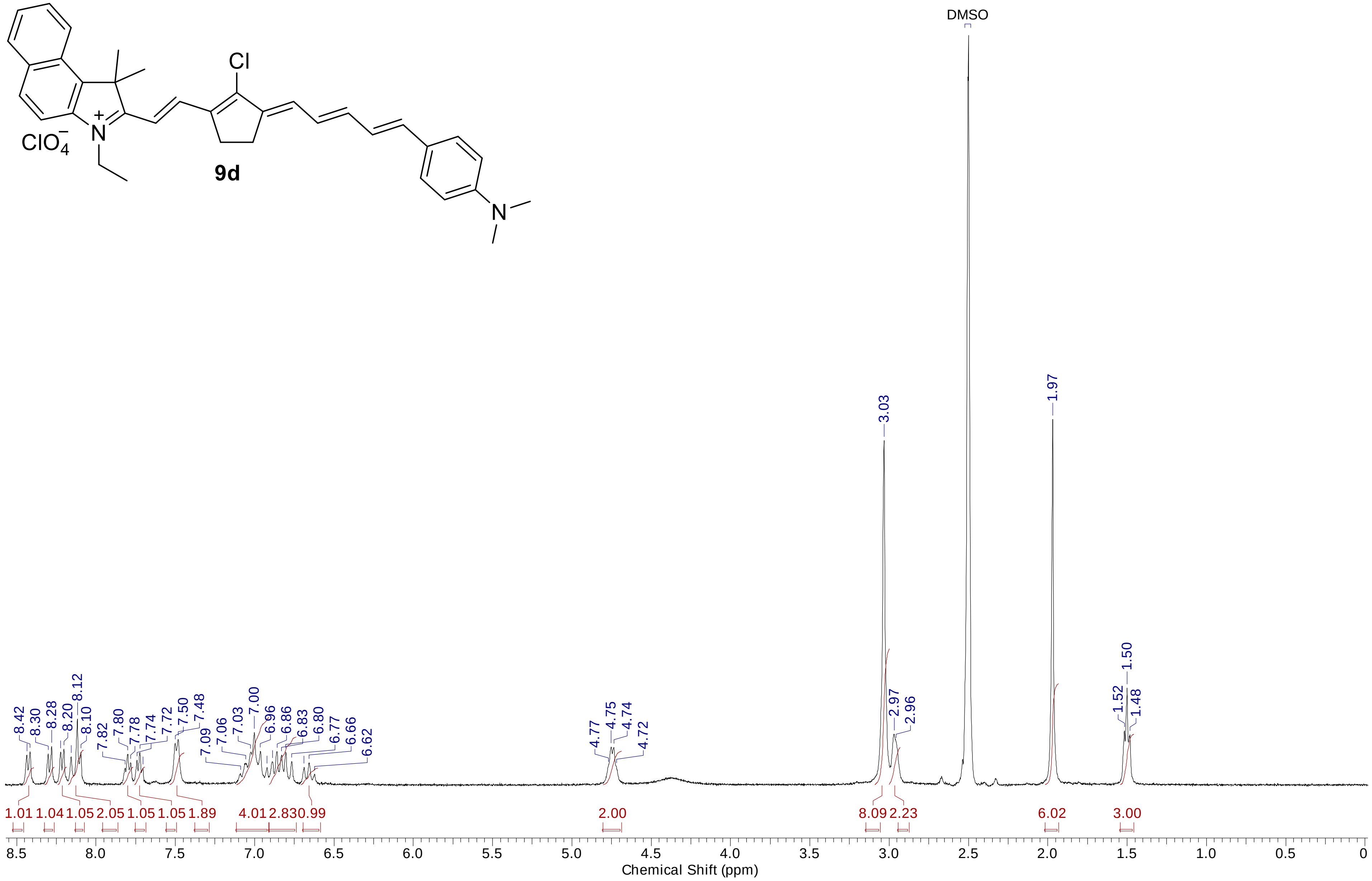
Cl
ClO4−
N
9d
DMSO
8.42 8.30 8.28 8.20 8.12 8.10 7.82 7.80 7.78 7.74 7.72 7.50 7.48 7.09 7.06 7.03 7.00 6.96 6.86 6.83 6.80 6.77 6.66 6.62
4.77 4.75 4.74 4.72
3.03 2.97 2.96
1.97
1.52 1.50 1.48
1.01 1.04 1.05 2.05 1.05 1.05 1.89 4.01 2.83 0.99 2.00 8.09 2.23 6.02 3.00
8.5 8.0 7.5 7.0 6.5 6.0 5.5 5.0 4.5 4.0 3.5 3.0 2.5 2.0 1.5 1.0 0.5 0
Chemical Shift (ppm)

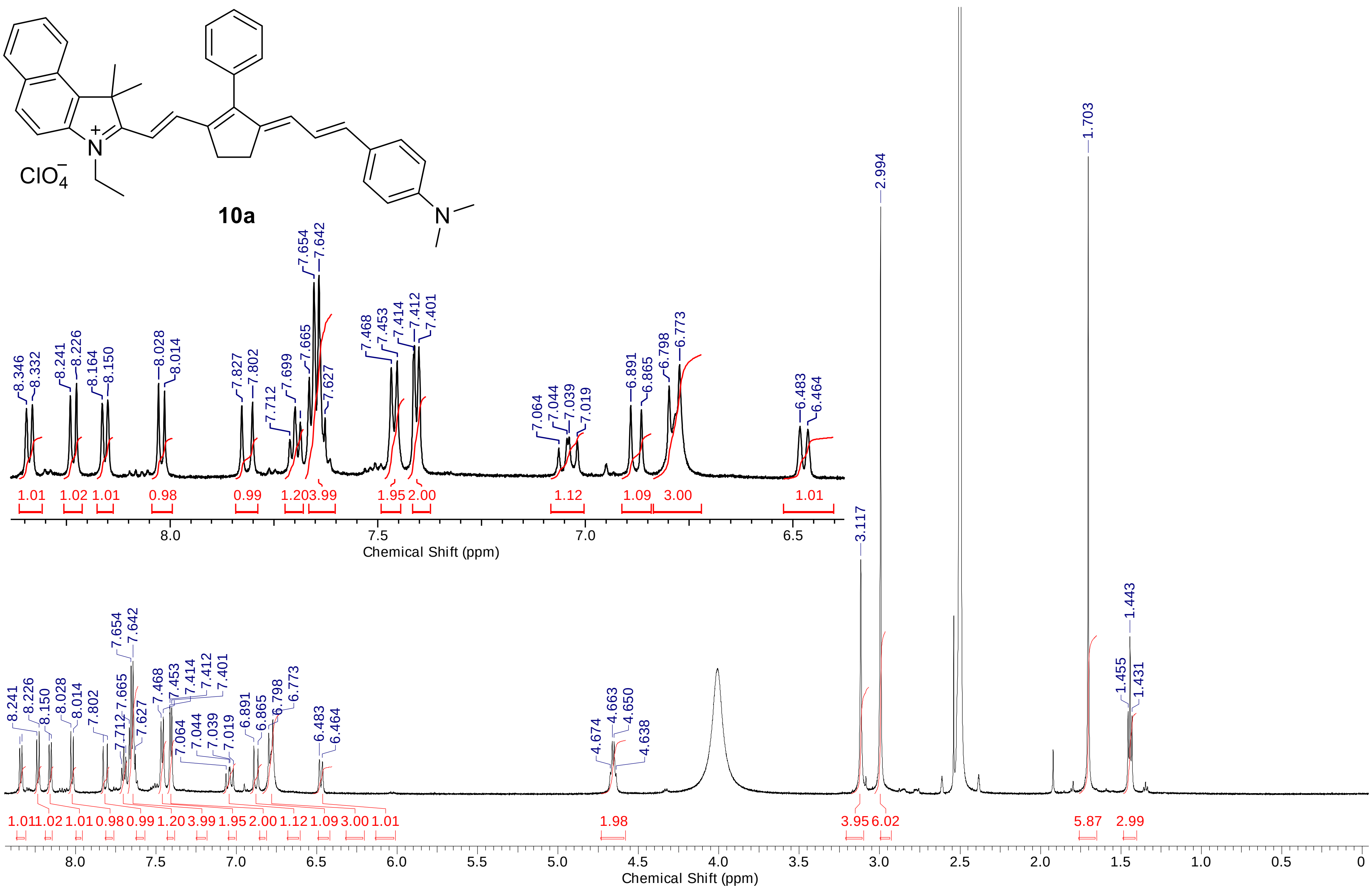

ClO4−
N+
N
10a
8.346
8.332
8.241
8.226
8.164
8.150
8.028
8.014
7.827
7.802
7.712
7.699
7.665
7.654
7.642
7.627
7.468
7.453
7.414
7.412
7.401
7.064
7.044
7.039
7.019
6.891
6.865
6.798
6.773
6.483
6.464
1.01 1.02 1.01 0.98 0.99 1.20 3.99 1.95 2.00 1.12 1.09 3.00 1.01
8.0 7.5 7.0 6.5
Chemical Shift (ppm)
4.674
4.663
4.650
4.638
3.117
2.994
1.703
1.455
1.443
1.431
1.01 1.02 1.01 0.98 0.99 1.20 3.99 1.95 2.00 1.12 1.09 3.00 1.01 1.98 3.95 6.02 5.87 2.99
8.0 7.5 7.0 6.5 6.0 5.5 5.0 4.5 4.0 3.5 3.0 2.5 2.0 1.5 1.0 0.5 0
Chemical Shift (ppm)

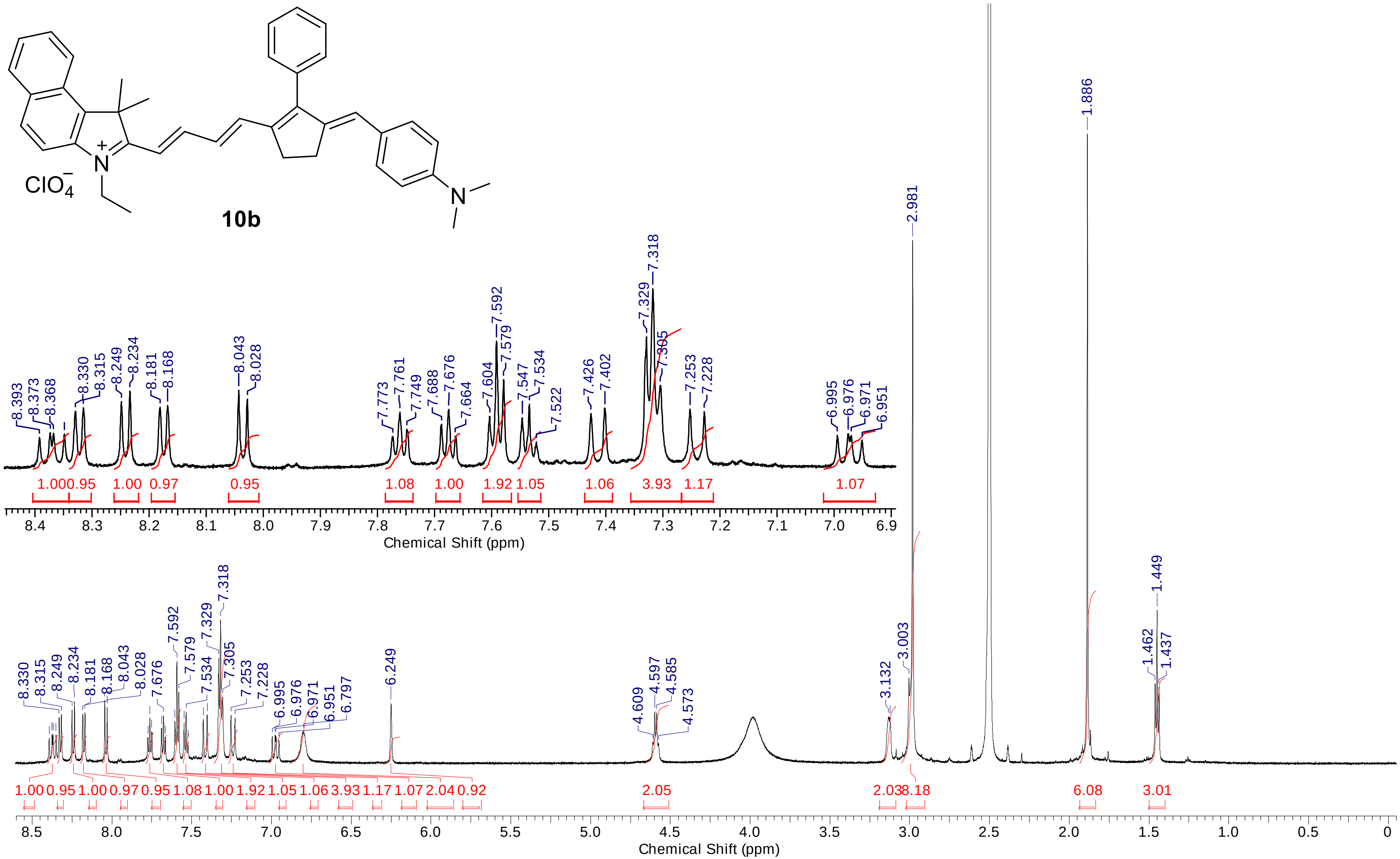
ClO4
N
10b
Chemical Shift (ppm)
Chemical Shift (ppm)

$^{13}C\{^1H\}$ NMR spectra of the synthesized compounds

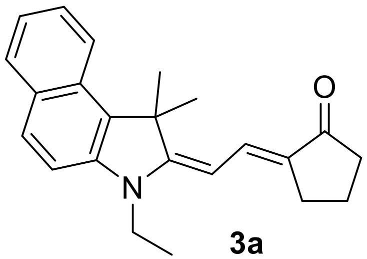

3a

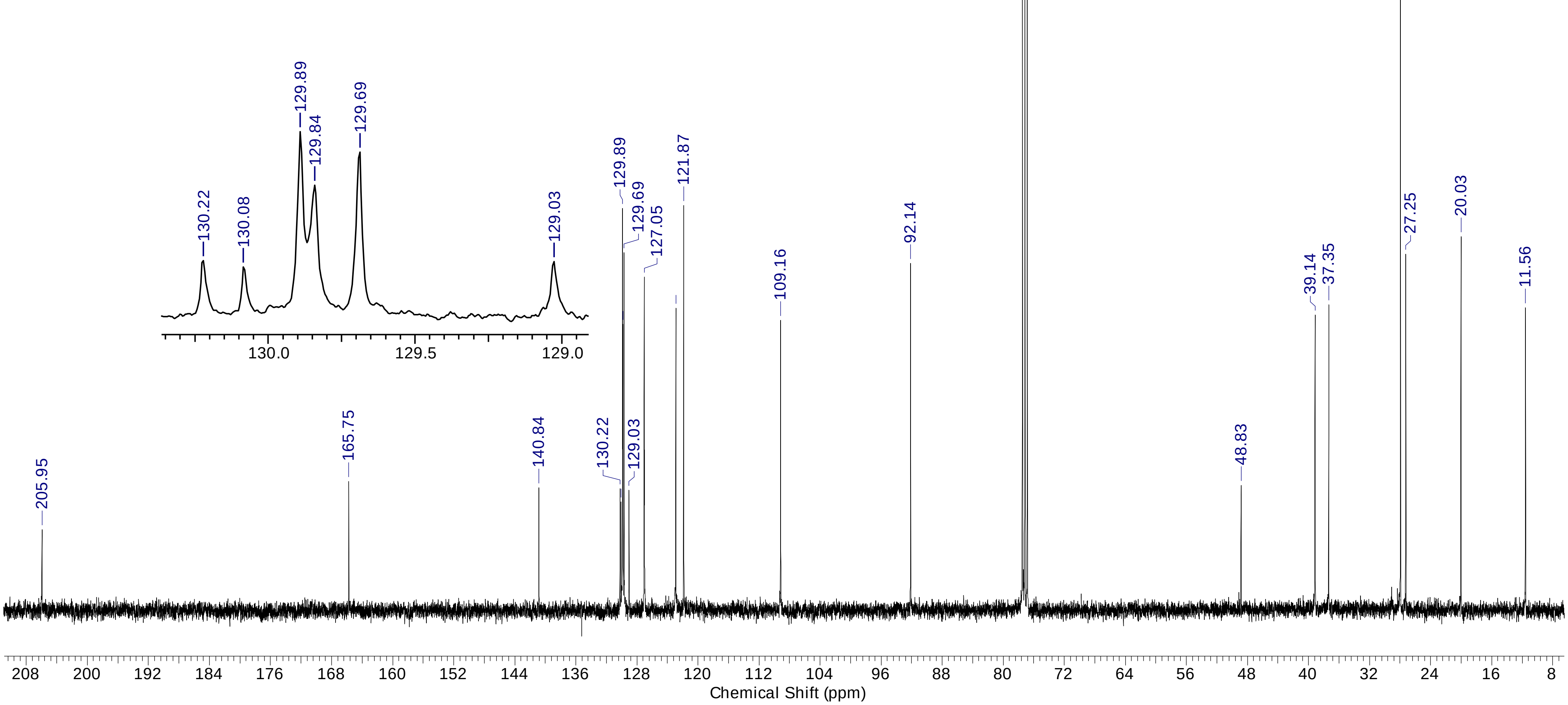

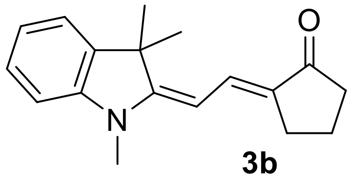

3b

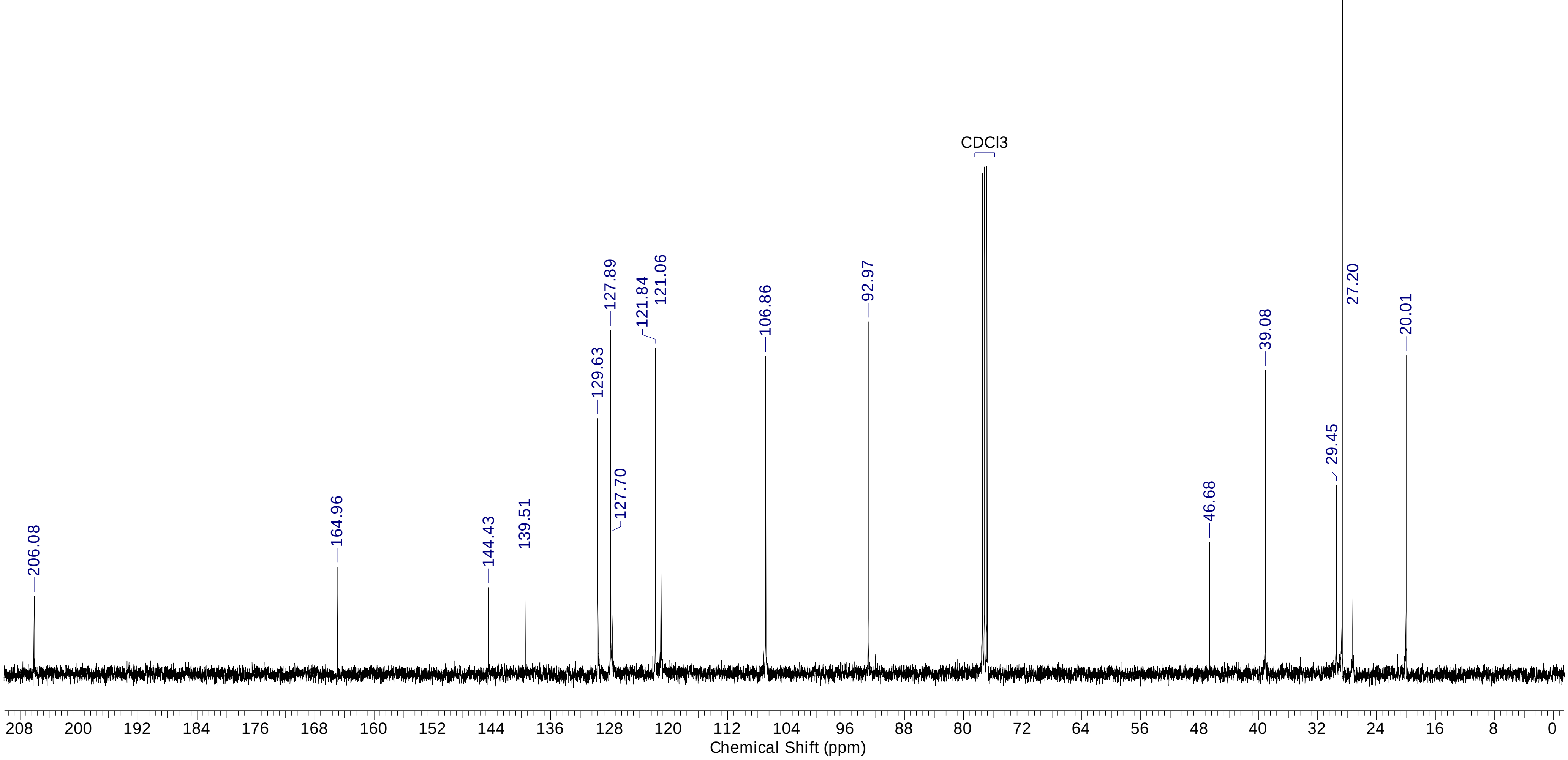

206.08
164.96
144.43
139.51
129.63
127.89
127.70
121.84
121.06
106.86
92.97
CDCl3
46.68
39.08
29.45
28.67
27.20
20.01
208 200 192 184 176 168 160 152 144 136 128 120 112 104 96 88 80 72 64 56 48 40 32 24 16 8 0
Chemical Shift (ppm)

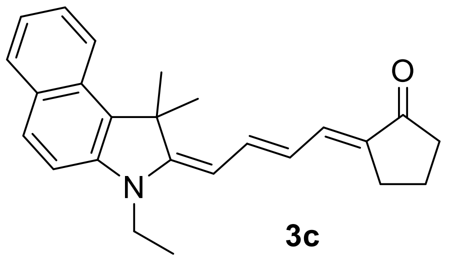

3c

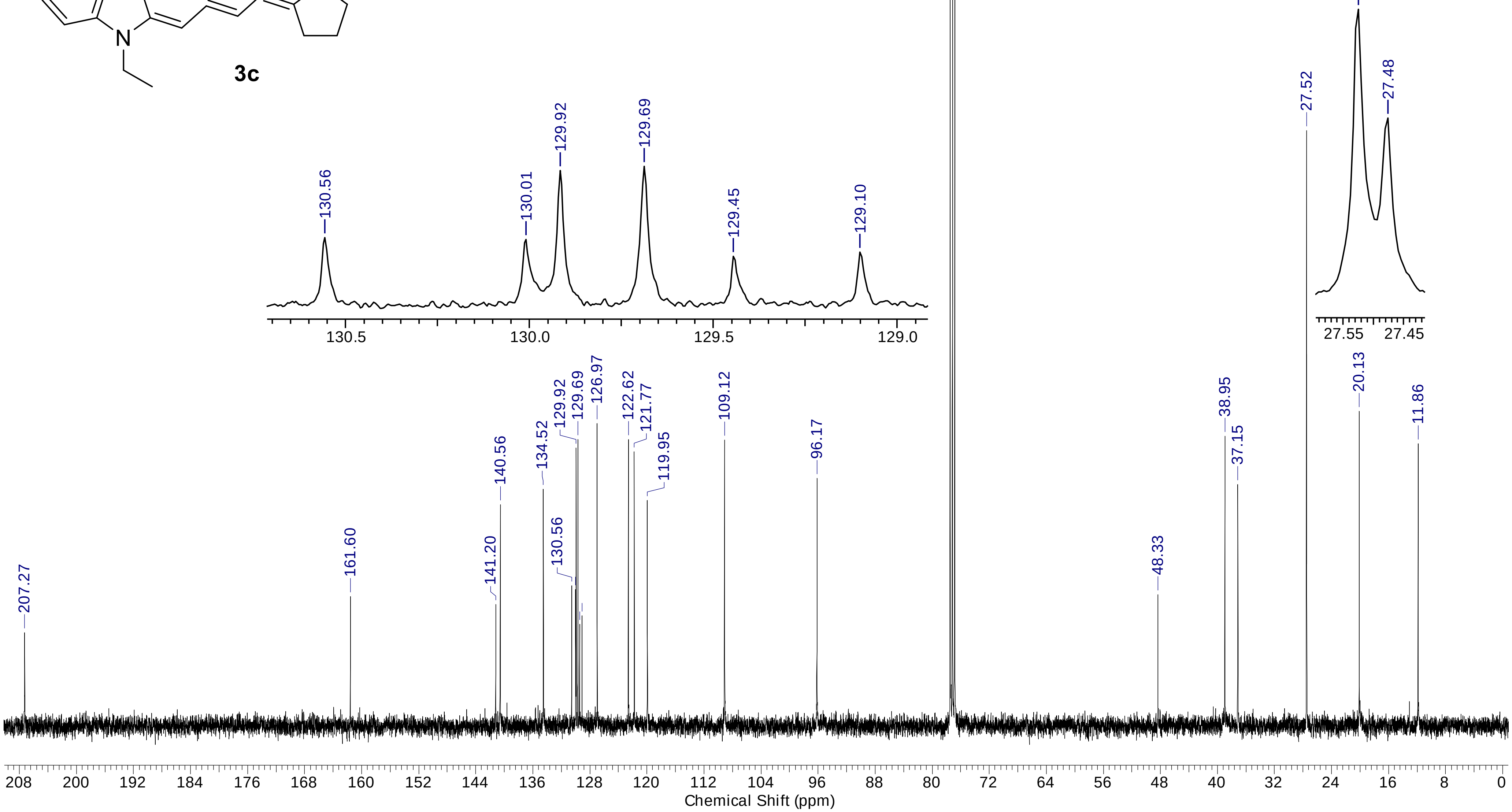
CDCl3
207.27
161.60
141.20
140.56
134.52
130.56
129.92
129.69
126.97
122.62
121.77
119.95
109.12
96.17
48.33
38.95
37.15
27.52
20.13
11.86
130.56
130.01
129.92
129.69
129.45
129.10
130.5
130.0
129.5
129.0
27.52
27.48
27.55
27.45
208 200 192 184 176 168 160 152 144 136 128 120 112 104 96 88 80 72 64 56 48 40 32 24 16 8 0
Chemical Shift (ppm)

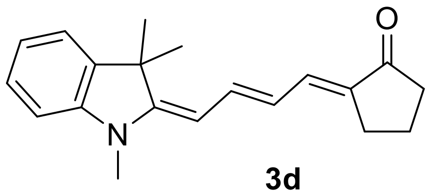
O
N

3d

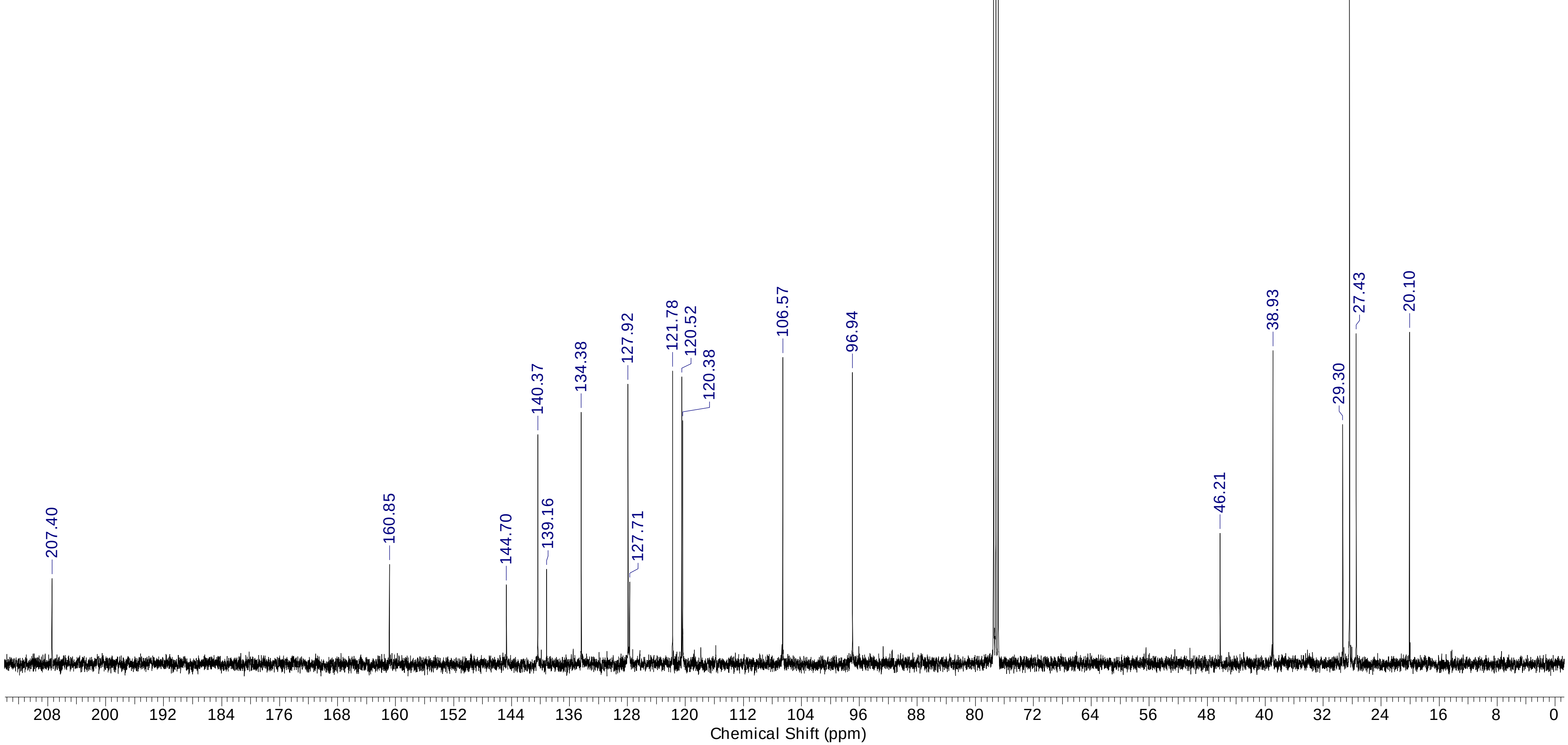
CDCl3
207.40
160.85
144.70
140.37
139.16
134.38
127.92
127.71
121.78
120.52
120.38
106.57
96.94
46.21
38.93
29.30
28.36
27.43
20.10
208 200 192 184 176 168 160 152 144 136 128 120 112 104 96 88 80 72 64 56 48 40 32 24 16 8 0
Chemical Shift (ppm)

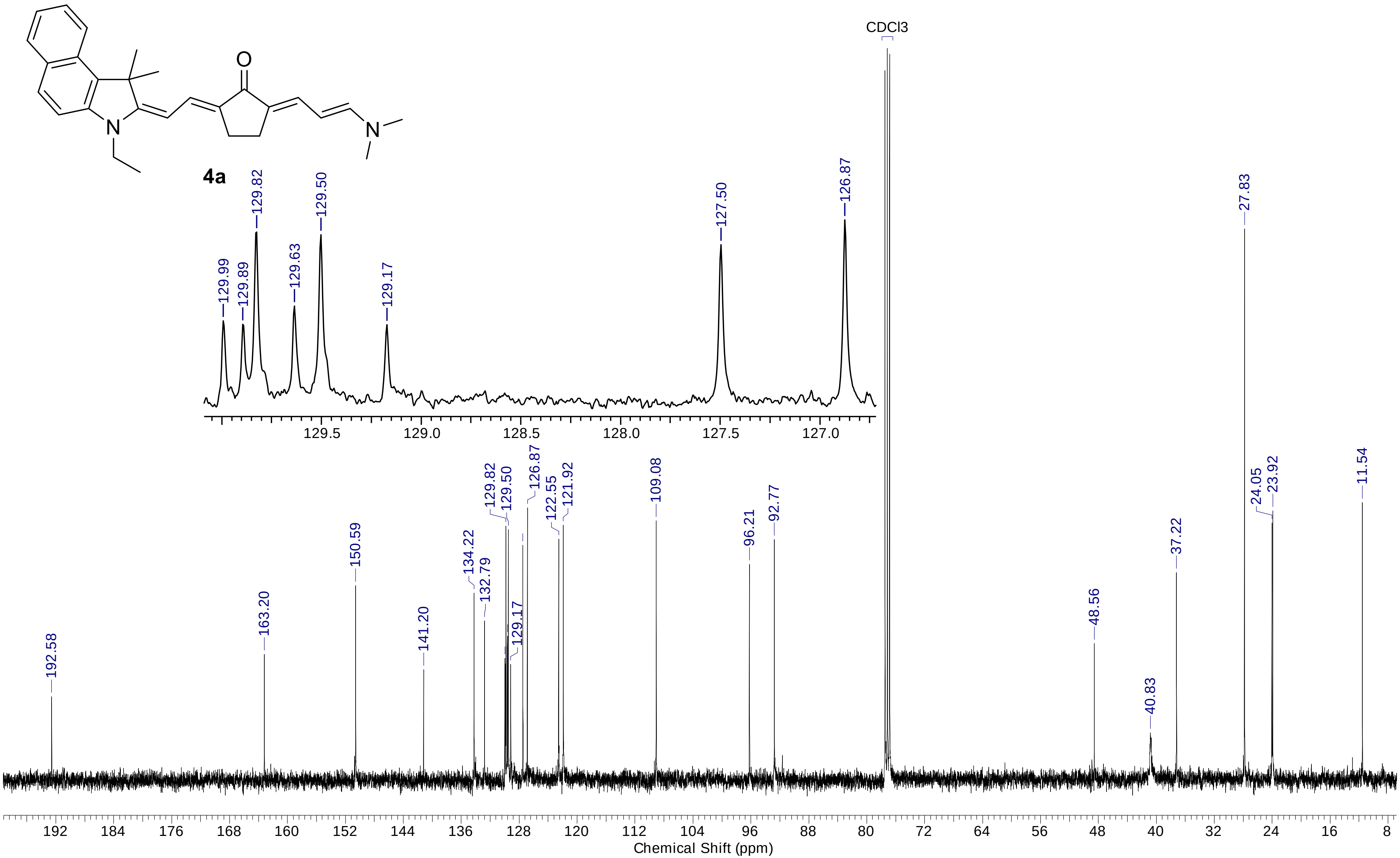
4a
CDCl3
129.99
129.89
129.82
129.63
129.50
129.17
127.50
126.87
192.58
163.20
150.59
141.20
134.22
132.79
129.82
129.50
129.17
126.87
122.55
121.92
109.08
96.21
92.77
48.56
40.83
37.22
27.83
24.05
23.92
11.54
Chemical Shift (ppm)

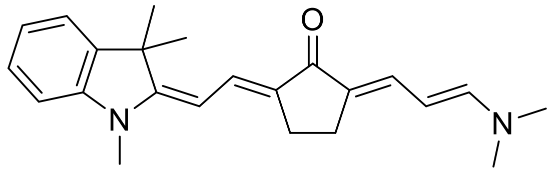
O
N
N

4b

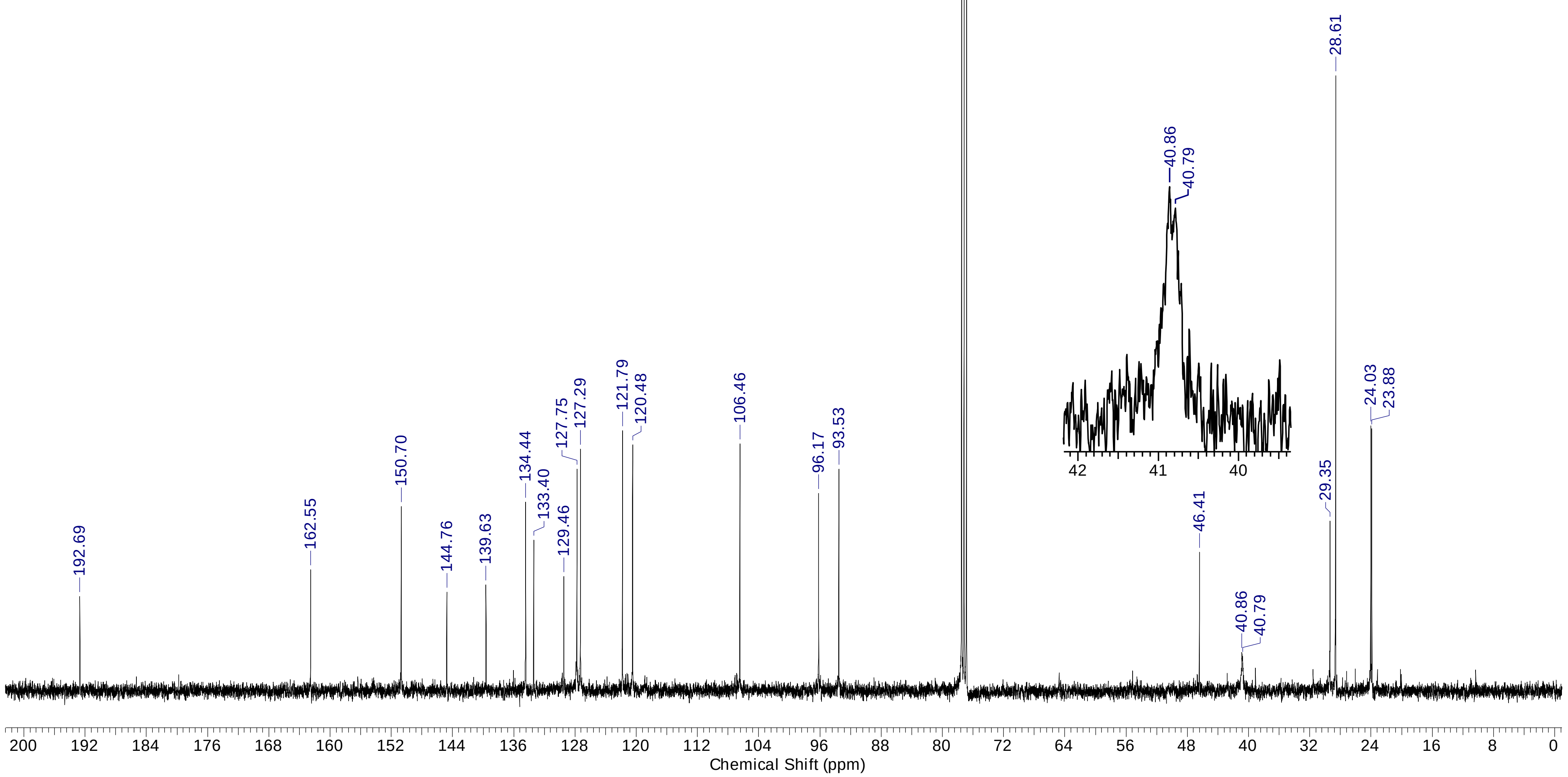
CDCl3
192.69
162.55
150.70
144.76
139.63
134.44
133.40
129.46
127.75
127.29
121.79
120.48
106.46
96.17
93.53
46.41
40.86
40.79
29.35
28.61
24.03
23.88
Chemical Shift (ppm)

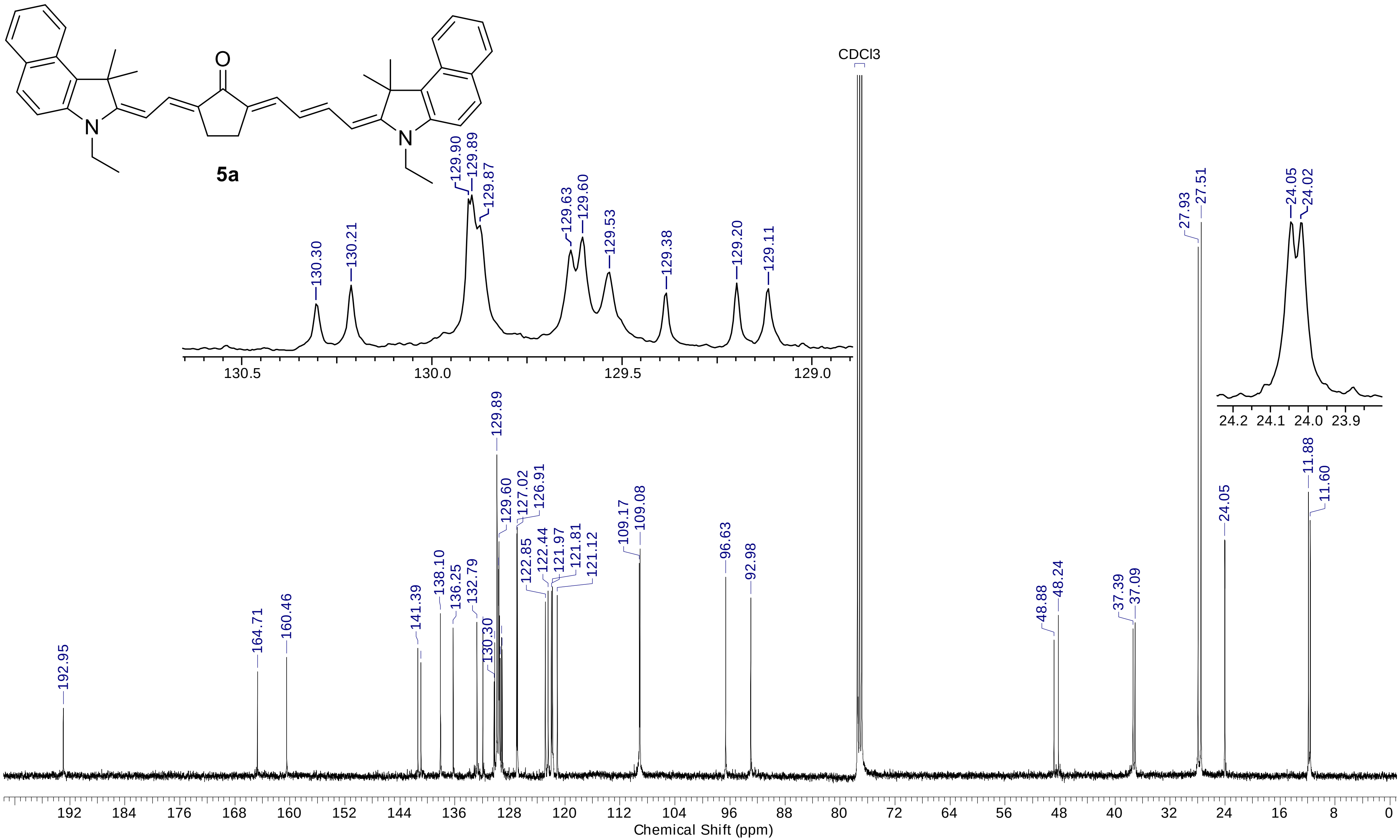

O
N
N
5a
CDCl3
130.30
130.21
129.90
129.89
129.87
129.63
129.60
129.53
129.38
129.20
129.11
130.5
130.0
129.5
129.0
27.93
27.51
24.05
24.02
24.2 24.1 24.0 23.9
192.95
164.71
160.46
141.39
138.10
136.25
132.79
130.30
129.89
129.60
127.02
126.91
122.85
122.44
121.97
121.81
121.12
109.17
109.08
96.63
92.98
48.88
48.24
37.39
37.09
24.05
11.88
11.60
192 184 176 168 160 152 144 136 128 120 112 104 96 88 80 72 64 56 48 40 32 24 16 8 0
Chemical Shift (ppm)

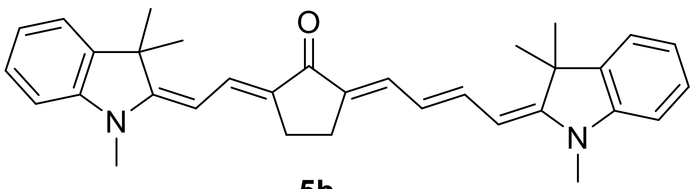
O
N
N

5b

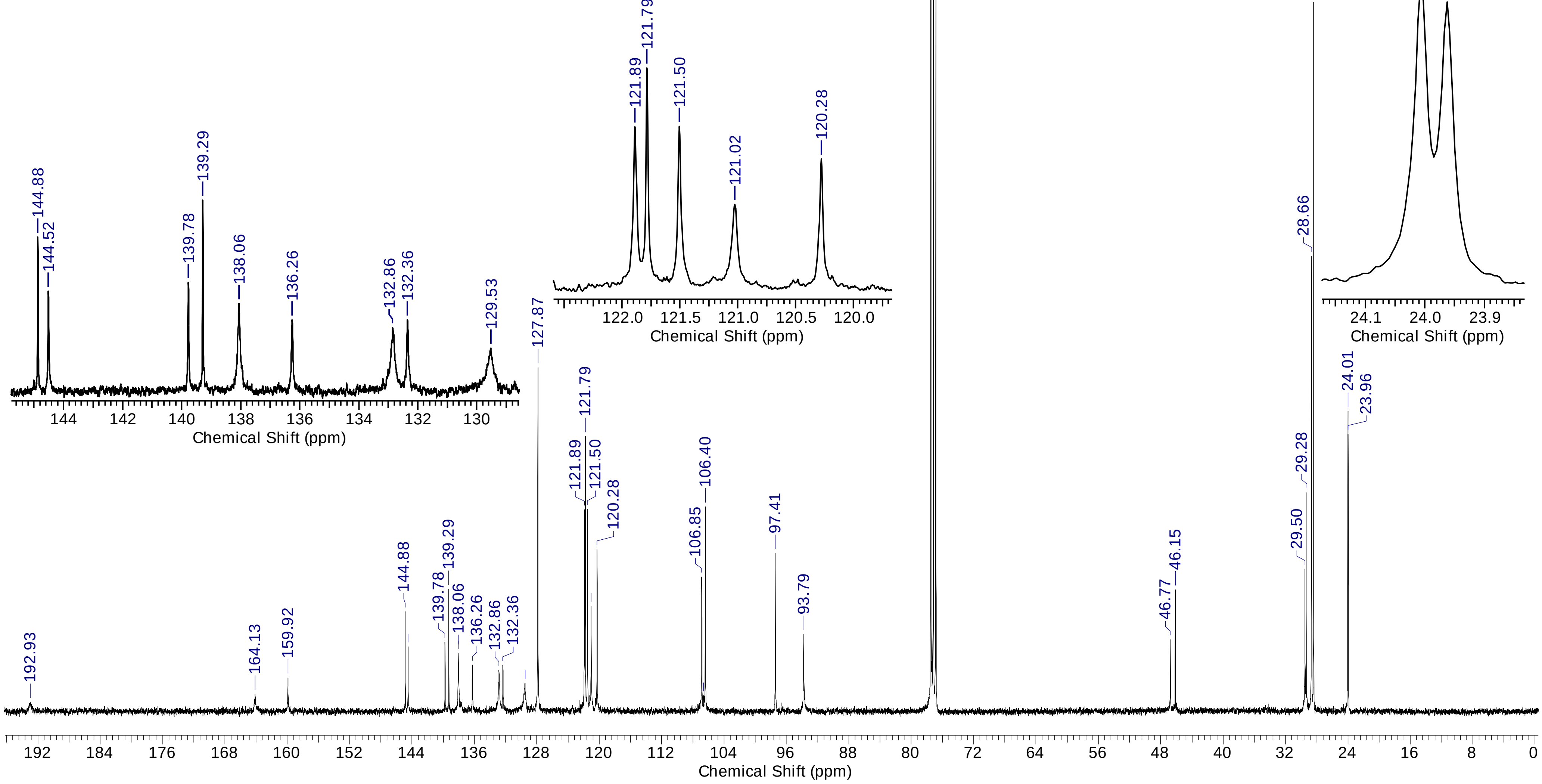
CDCl3
192.93
164.13
159.92
144.88
139.78
139.29
138.06
136.26
132.86
132.36
127.87
121.89
121.79
121.50
120.28
106.85
106.40
97.41
93.79
46.77
46.15
29.50
29.28
28.66
28.39
24.01
23.96
Chemical Shift (ppm)
144.52
129.53

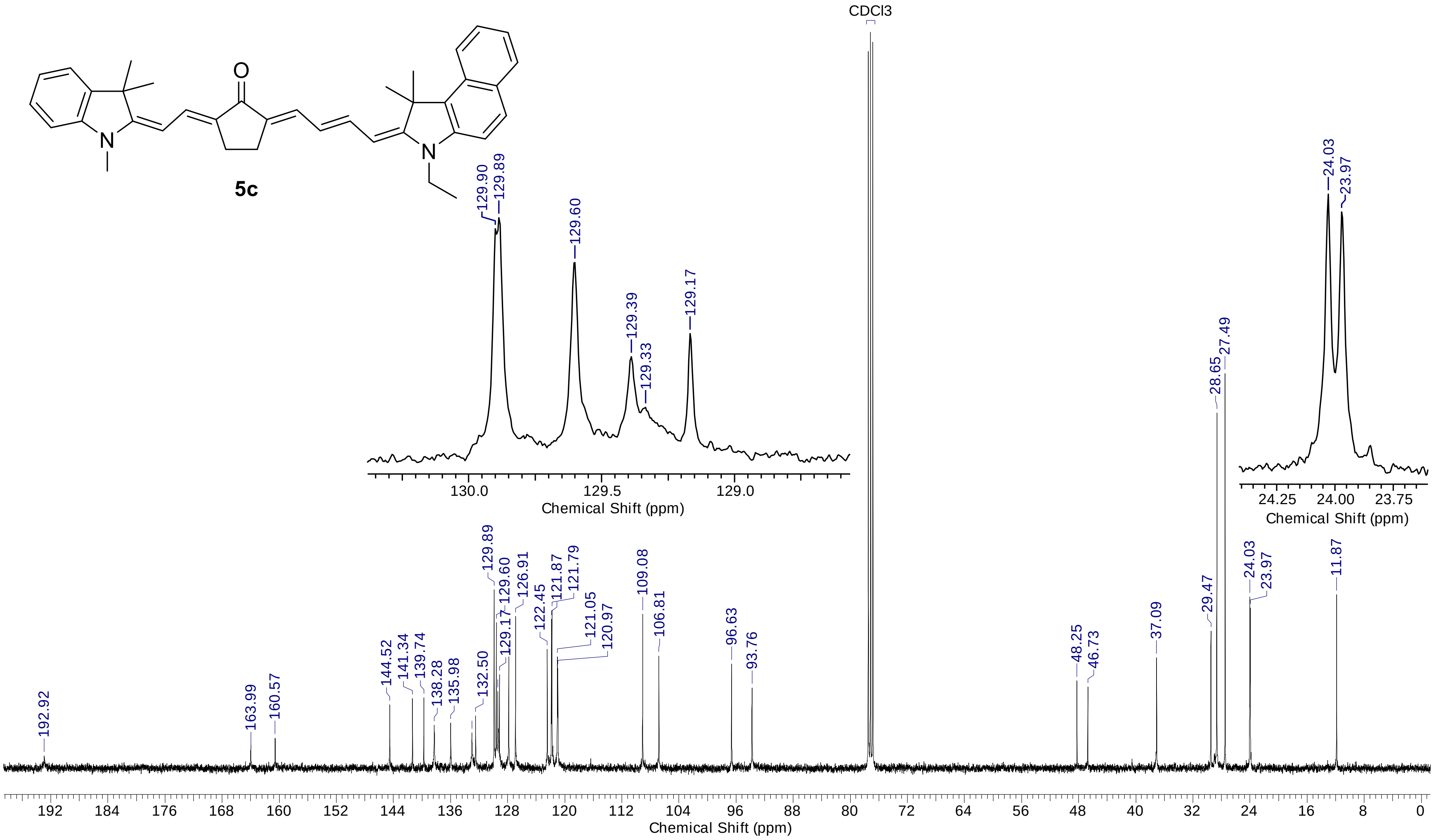
CDCl3
129.90
129.89
129.60
129.39
129.33
129.17
130.0
129.5
129.0
Chemical Shift (ppm)
24.03
23.97
24.25
24.00
23.75
Chemical Shift (ppm)
192.92
163.99
160.57
144.52
141.34
139.74
138.28
135.98
132.50
129.89
129.60
129.17
126.91
122.45
121.87
121.79
121.05
120.97
109.08
106.81
96.63
93.76
48.25
46.73
37.09
29.47
28.65
27.49
24.03
23.97
11.87
192
184
176
168
160
152
144
136
128
120
112
104
96
88
80
72
64
56
48
40
32
24
16
8
0
Chemical Shift (ppm)

5c

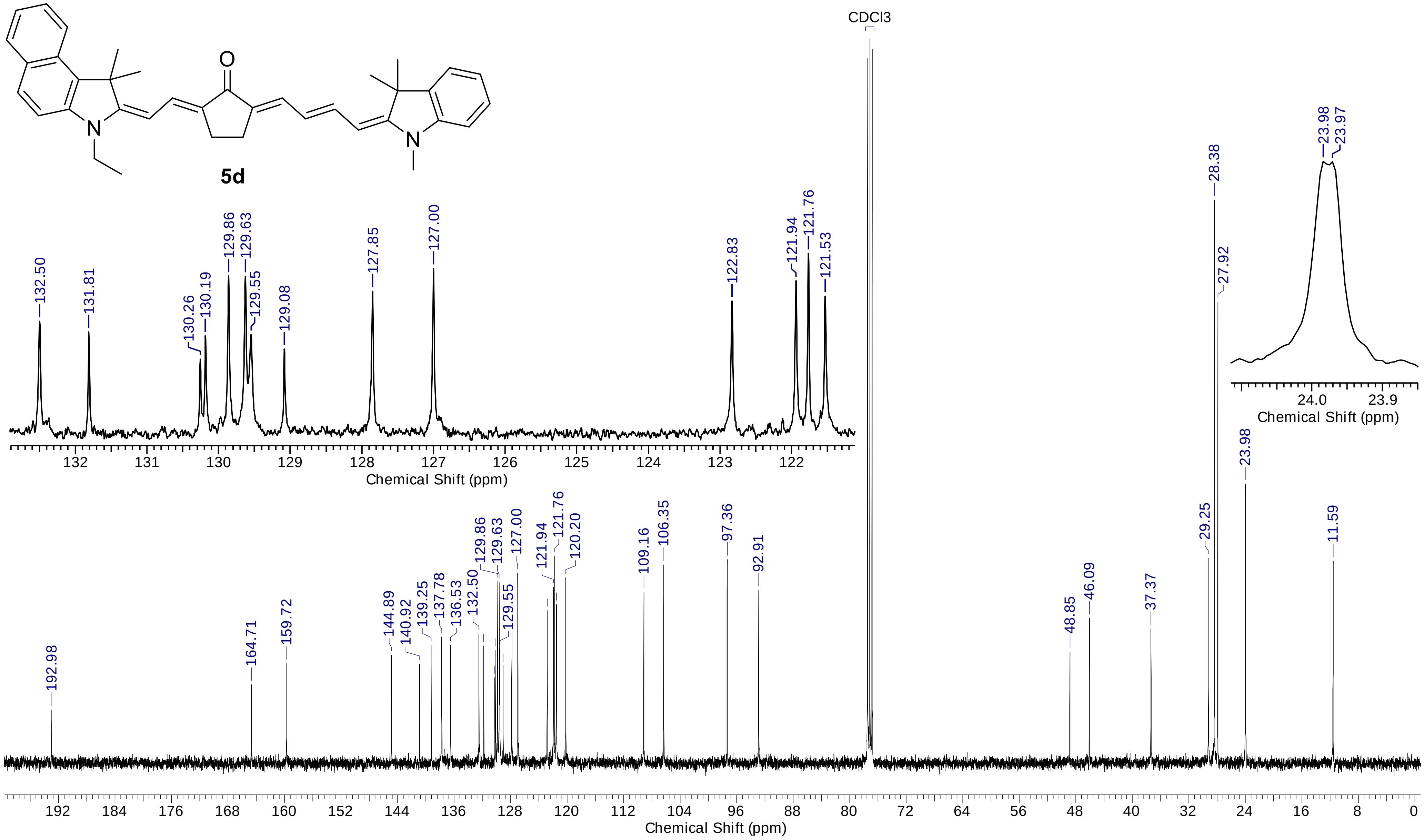
O
N
N
5d
CDCl3
132.50
131.81
130.26
130.19
129.86
129.63
129.55
129.08
127.85
127.00
122.83
121.94
121.76
121.53
Chemical Shift (ppm)
23.98
23.97
Chemical Shift (ppm)
192.98
164.71
159.72
144.89
140.92
139.25
137.78
136.53
132.50
129.86
129.63
129.55
127.00
121.94
121.76
120.20
109.16
106.35
97.36
92.91
48.85
46.09
37.37
29.25
28.38
27.92
23.98
11.59
Chemical Shift (ppm)

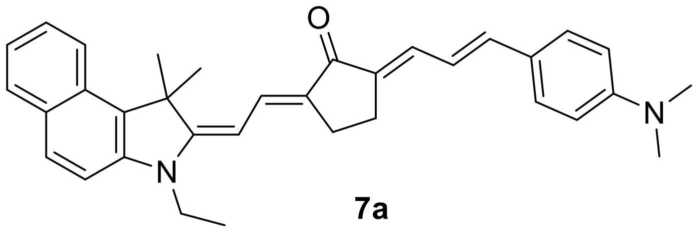
O
N
N

7a

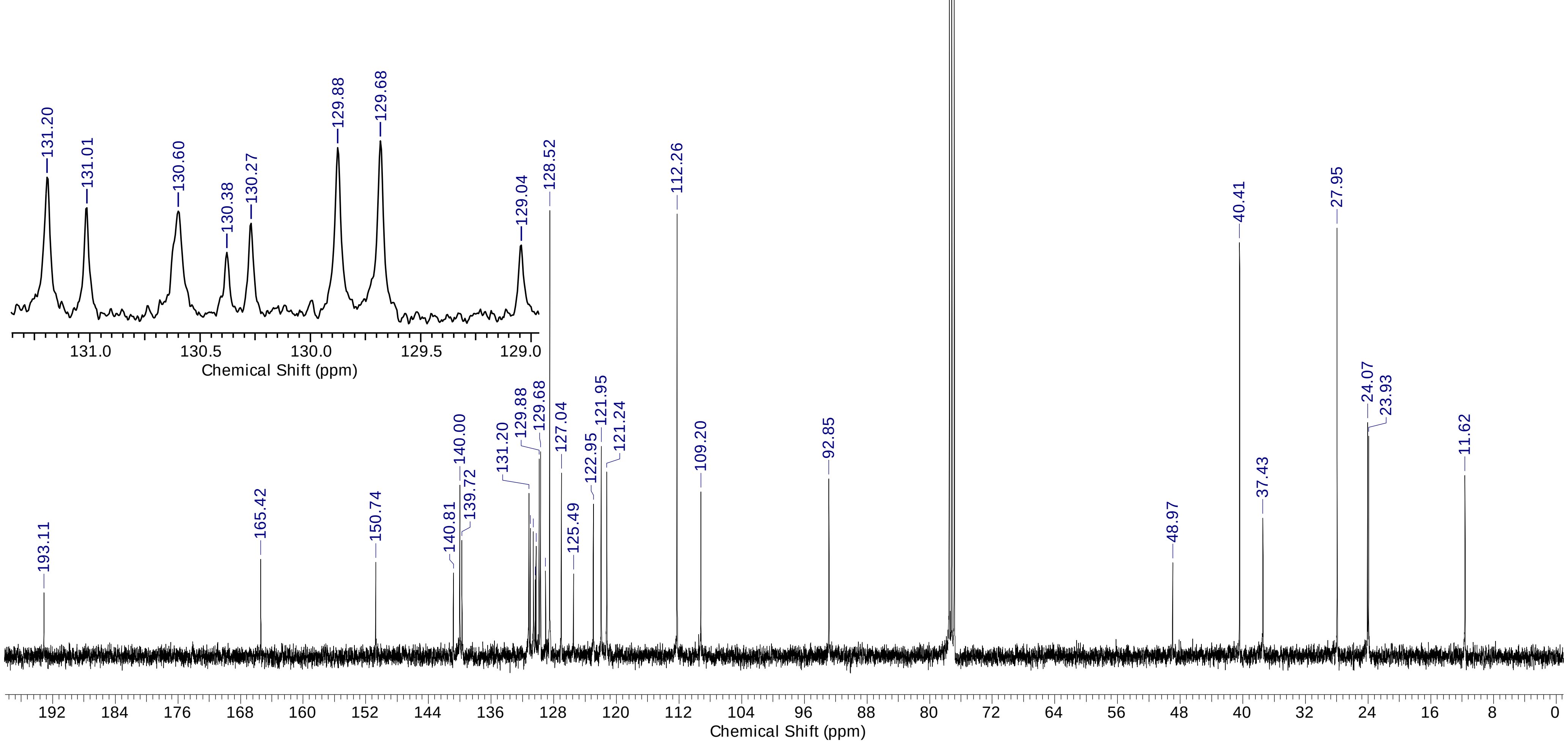
CDCl3
131.20
131.01
130.60
130.38
130.27
129.88
129.68
129.04
131.0
130.5
130.0
129.5
129.0
Chemical Shift (ppm)
193.11
165.42
150.74
140.81
140.00
139.72
131.20
129.88
129.68
128.52
127.04
125.49
122.95
121.95
121.24
112.26
109.20
92.85
48.97
40.41
37.43
27.95
24.07
23.93
11.62
192
184
176
168
160
152
144
136
128
120
112
104
96
88
80
72
64
56
48
40
32
24
16
8
0
Chemical Shift (ppm)

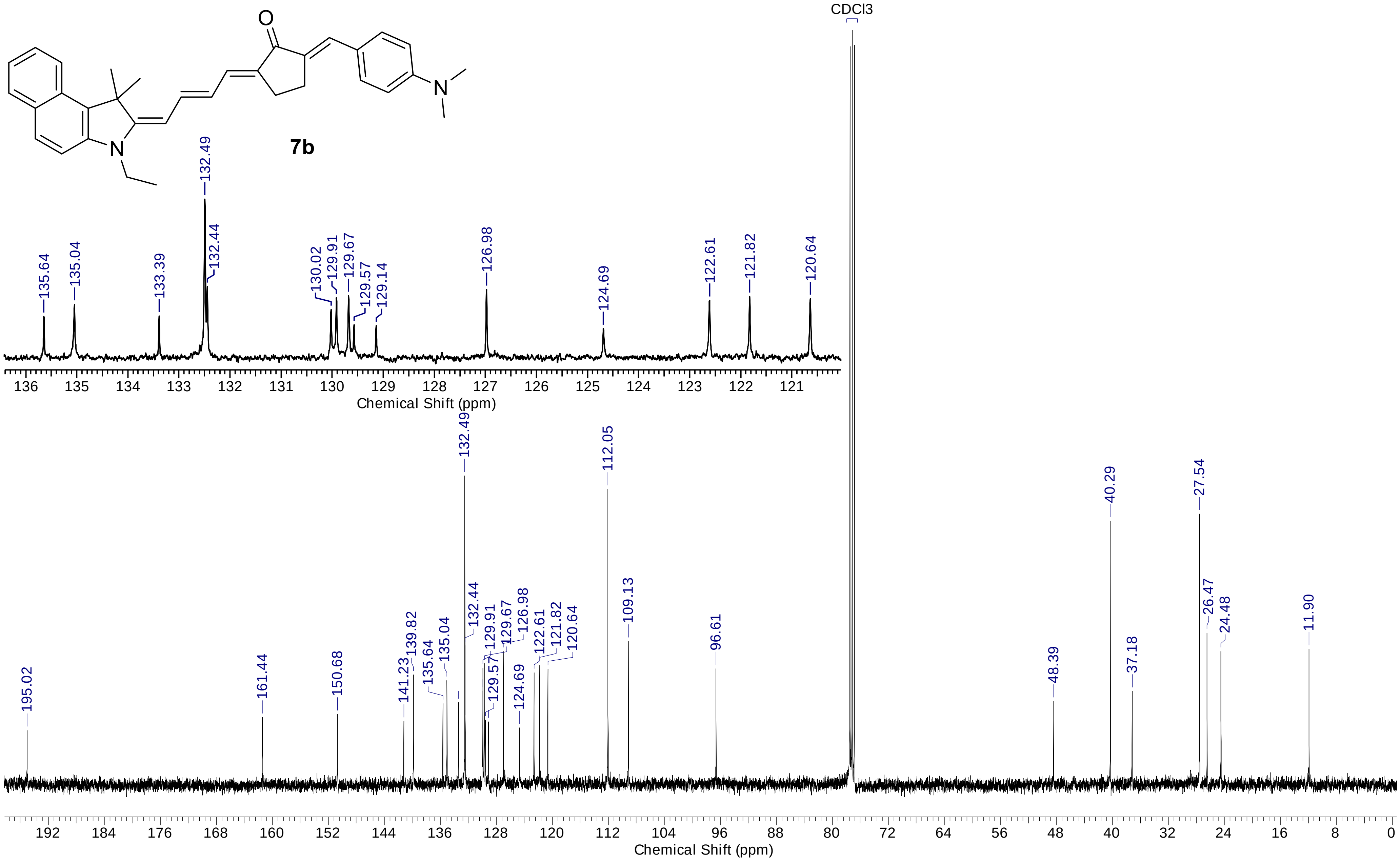
7b
O
N
CDCl3
135.64
135.04
133.39
132.49
132.44
130.02
129.91
129.67
129.57
129.14
126.98
124.69
122.61
121.82
120.64
Chemical Shift (ppm)
195.02
161.44
150.68
141.23
139.82
112.05
109.13
96.61
48.39
40.29
37.18
27.54
26.47
24.48
11.90
Chemical Shift (ppm)

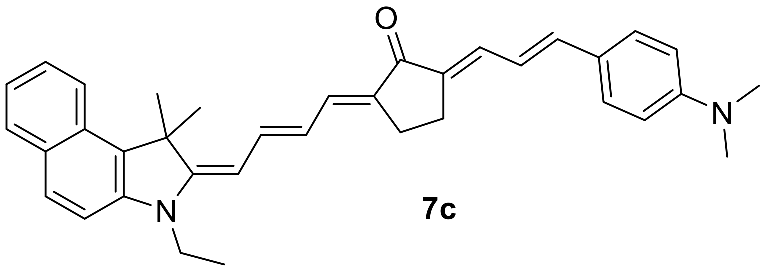

O
N
N
7c

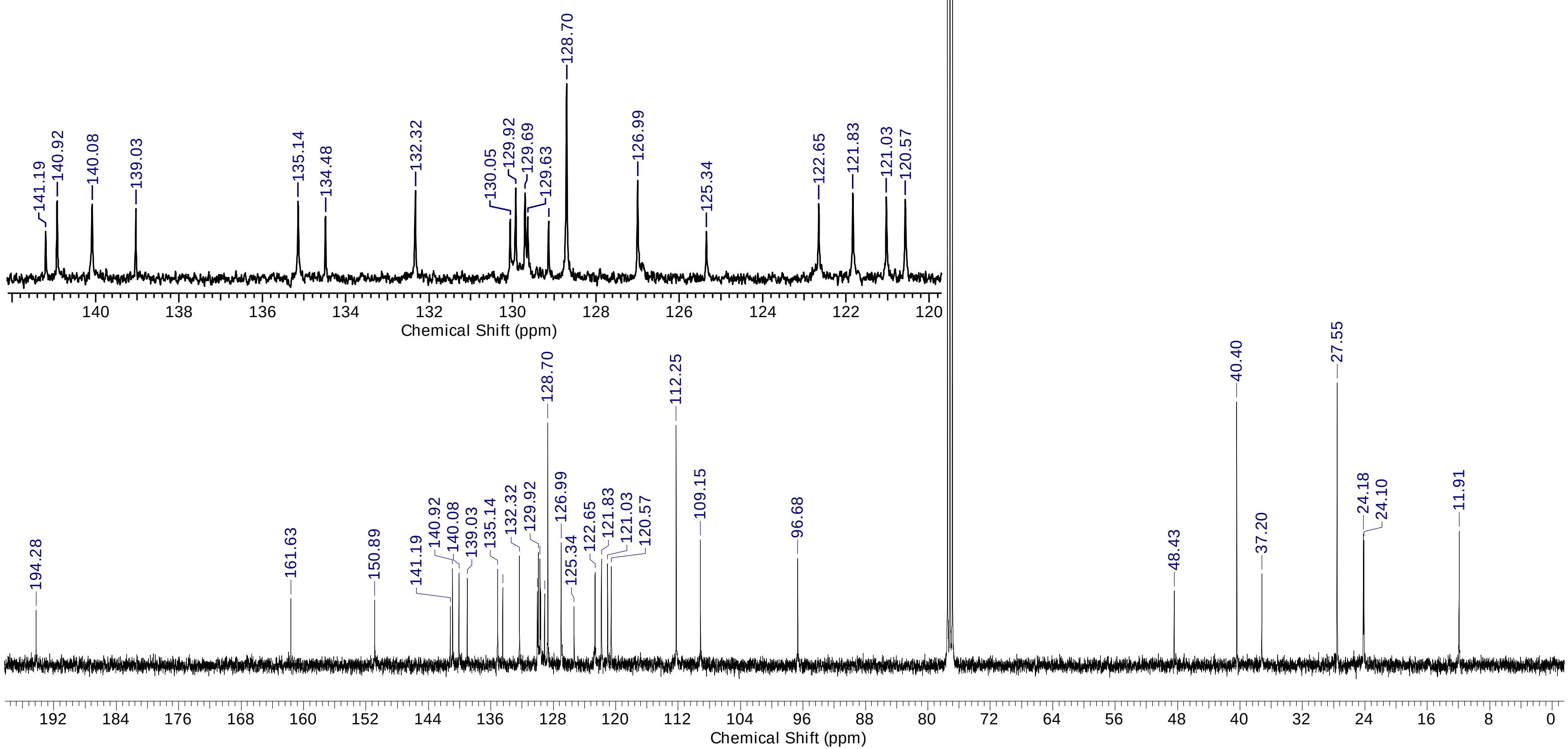

CDCl3
141.19
140.92
140.08
139.03
135.14
134.48
132.32
130.05
129.92
129.69
129.63
128.70
126.99
125.34
122.65
121.83
121.03
120.57
Chemical Shift (ppm)
194.28
161.63
150.89
112.25
109.15
96.68
48.43
40.40
37.20
27.55
24.18
24.10
11.91
Chemical Shift (ppm)

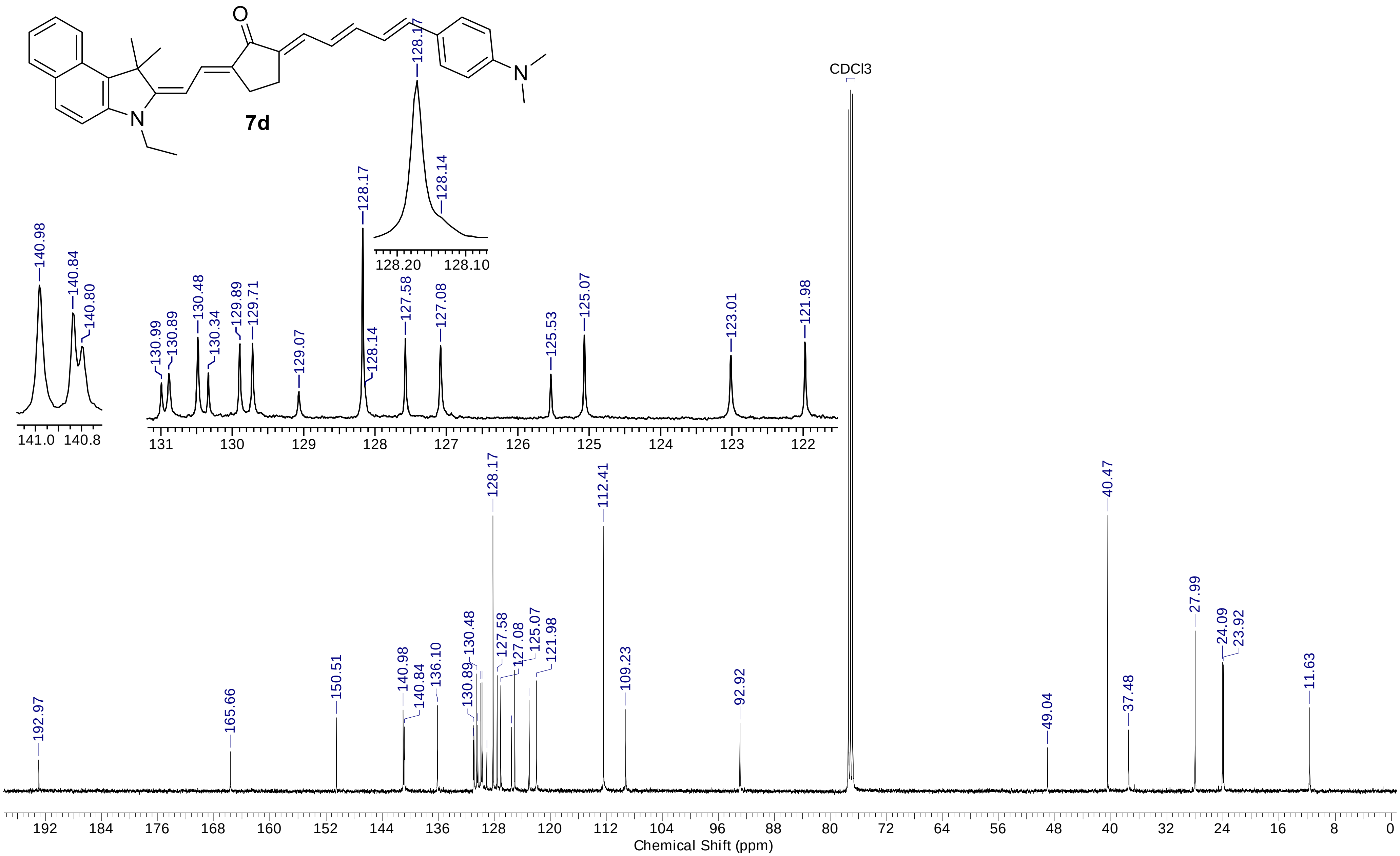
O
N
N
7d
CDCl3
192.97
165.66
150.51
140.98
140.84
140.80
136.10
130.99
130.89
130.48
130.34
129.89
129.71
129.07
128.17
128.14
127.58
127.08
125.53
125.07
123.01
121.98
112.41
109.23
92.92
49.04
40.47
37.48
27.99
24.09
23.92
11.63
141.0 140.8
131 130 129 128 127 126 125 124 123 122
128.20 128.10
192 184 176 168 160 152 144 136 128 120 112 104 96 88 80 72 64 56 48 40 32 24 16 8 0
Chemical Shift (ppm)

HRMS spectra of the synthesized compounds

**Data File**	29.d	**Sample Name**	MSO00936
**Sample Type**	Sample	**Position**	P1-D2
**Instrument Name**	Instrument 1	**User Name**	Denis V.Bylina
**Acq Method**	Fast_Gradient_HRMS_pos_Lock_01312023.m	**Acquired Time**	6/8/2024 7:49:22 PM (UTC+03:00)
**IRM Calibration Status**	Success	**DA Method**	1.m
**Comment**	S.Melnichuk		
**Sample Group**		**Info.**	Agilent 6224 TOF LC/MS
**MFC**	C23H25NO	**Stream Name**	LC 1
**Acquisition Time (Local)**	6/8/2024 7:49:22 PM (UTC+03:00)	**Acquisition SW Version**	6200 series TOF/6500 series Q-TOF B.08.00 (B8058.0)
**TOF Driver Version**	8.00.00	**TOF Firmware Version**	8.643
**Tune Mass Range Max.**	1700		

Will not repeat this information heading for the following compounds

Compound Table

Label	Tgt Score	Mass Error (ppm)	Tgt Formula	Obs. RT	Ref. Mass	Obs. Mass
Cpd 1: C23 H25 N O; 3.270	98.98	-0.59	C23 H25 N O	3.27	331.19361	331.19342

Obs. *m/z*	Obs. RT	Obs. Mass	Tgt Formula	Tgt Mass	Tgt Mass Error (ppm)	RT Diff.	Find Cpds Algorithm
332.20078	3.27	331.19342	C23 H25 N O	331.19361	-0.59	Find By Formula	

Compound Chromatograms

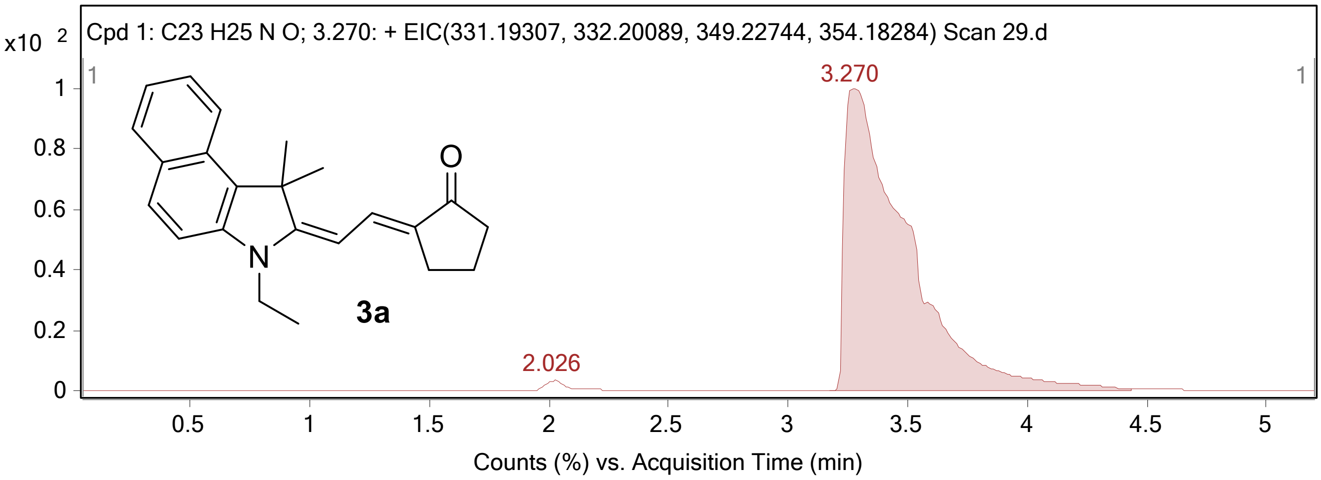

MS Zoomed Spectrum

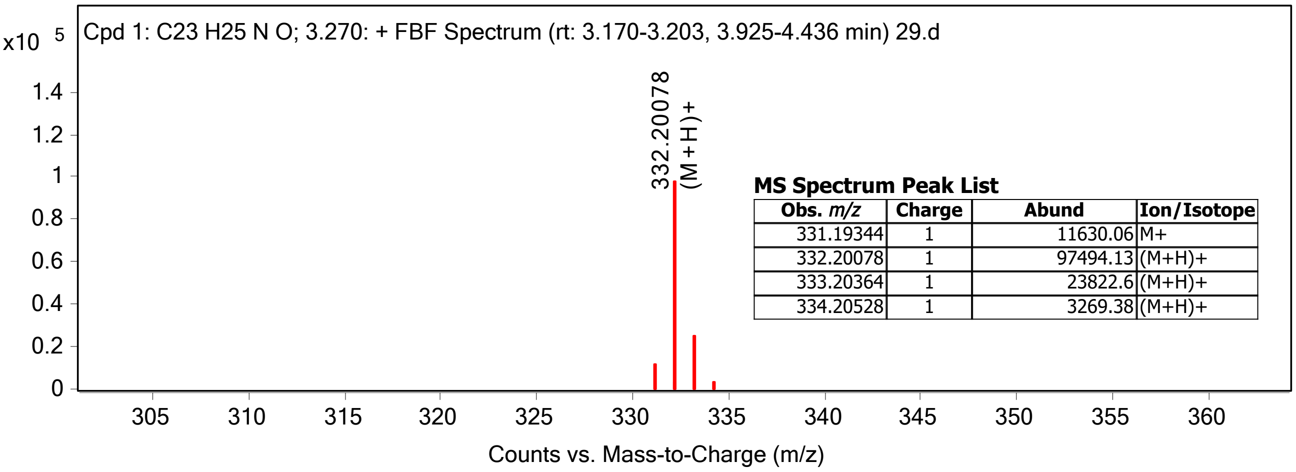

MS Spectrum Peak List

Obs. *m/z*	Charge	Abund	Ion/Isotope
331.19344	1	11630.06	M+
332.20078	1	97494.13	(M+H)+
333.20364	1	23822.6	(M+H)+
334.20528	1	3269.38	(M+H)+

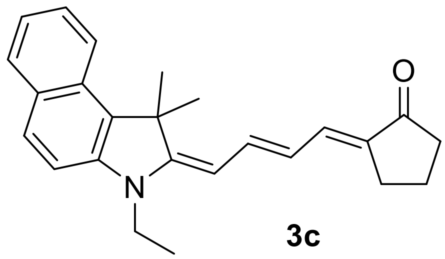

3c

Compound Table

Label	Tgt Score	Mass Error (ppm)	Tgt Formula	Obs. RT	Ref. Mass	Obs. Mass
Cpd 1: C25 H27 N O; 3.539	96.03	-3.35	C25 H27 N O	3.539	357.20926	357.20807

Obs. *m/z*	Obs. RT	Obs. Mass	Tgt Formula	Tgt Mass	Tgt Mass Error (ppm)	RT Diff.	Find Cpds Algorithm
358.21512	3.539	357.20807	C25 H27 N O	357.20926	-3.35	Find By Formula	

Compound Chromatograms

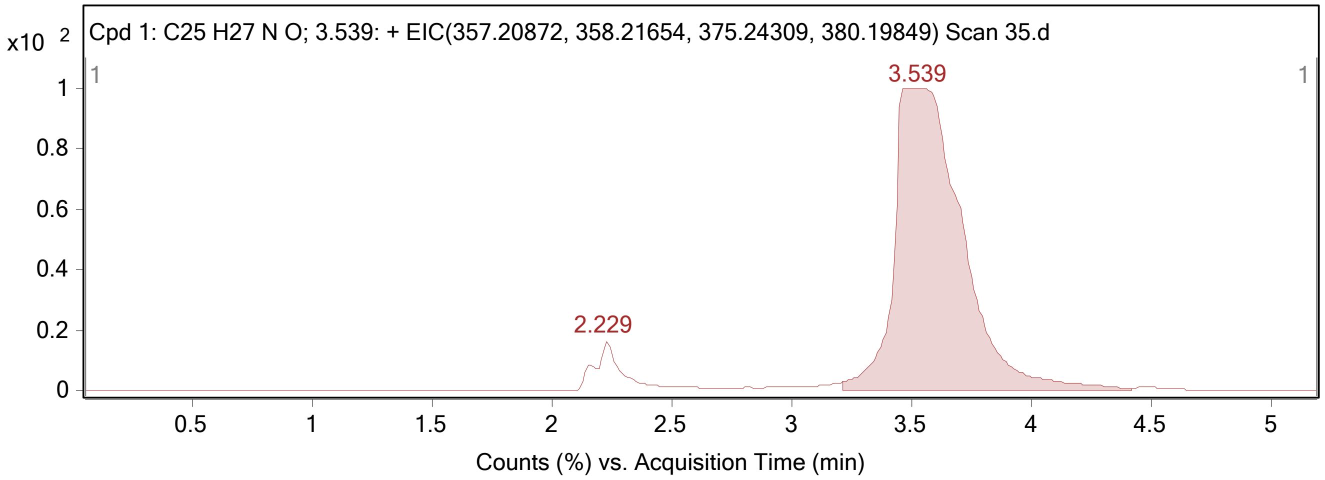

MS Zoomed Spectrum

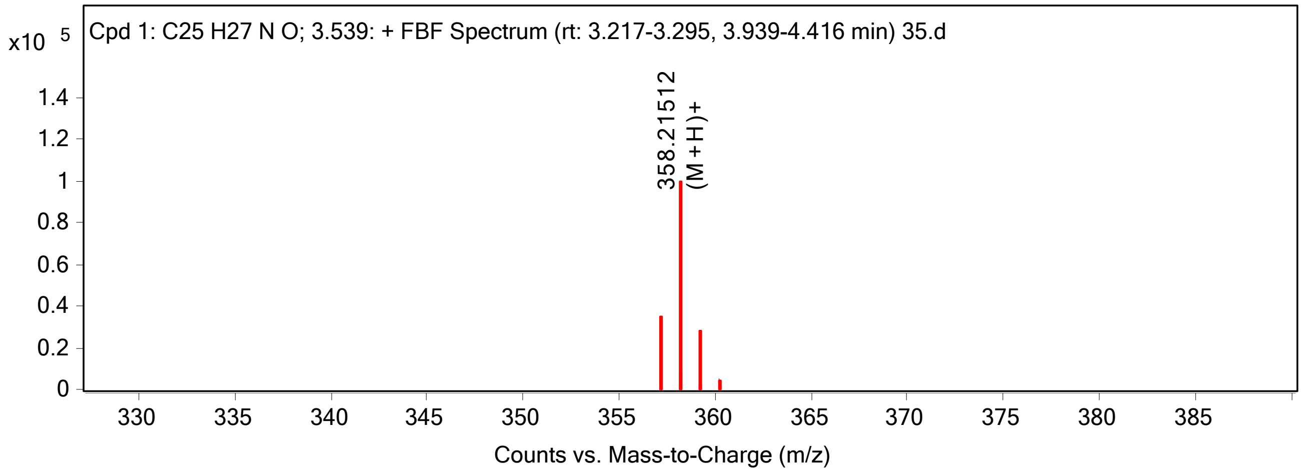

MS Spectrum Peak List

Obs. *m/z*	Charge	Abund	Ion/Isotope
357.20776	1	34583.02	M+
358.21512	1	100026.84	(M+H)+
359.21872	1	25275.77	(M+H)+
360.2241	1	5211.28	(M+H)+

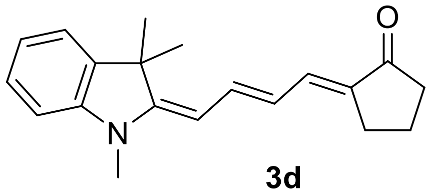

3d

Compound Table

Label	Tgt Score	Mass Error (ppm)	Tgt Formula	Obs. RT	Ref. Mass	Obs. Mass
Cpd 2: C20 H23 N O; 3.178	94.58	-3.87	C20 H23 N O	3.178	293.17796	293.17683

Obs. *m/z*	Obs. RT	Obs. Mass	Tgt Formula	Tgt Mass	Tgt Mass Error (ppm)	RT Diff.	Find Cpds Algorithm
294.18385	3.178	293.17683	C20 H23 N O	293.17796	-3.87	Find By Formula	

Compound Chromatograms

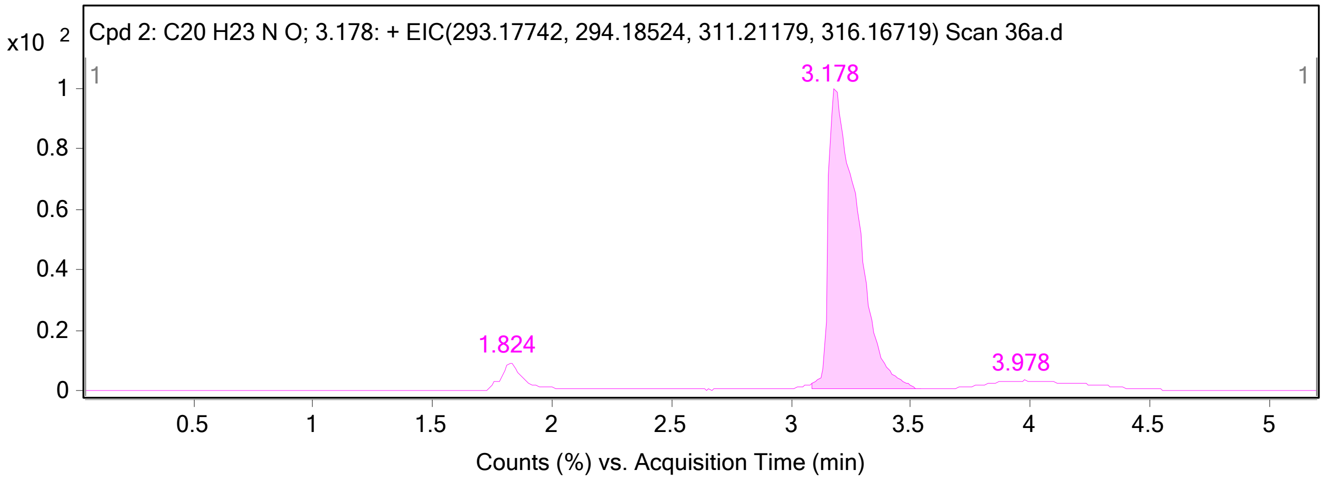

MS Zoomed Spectrum

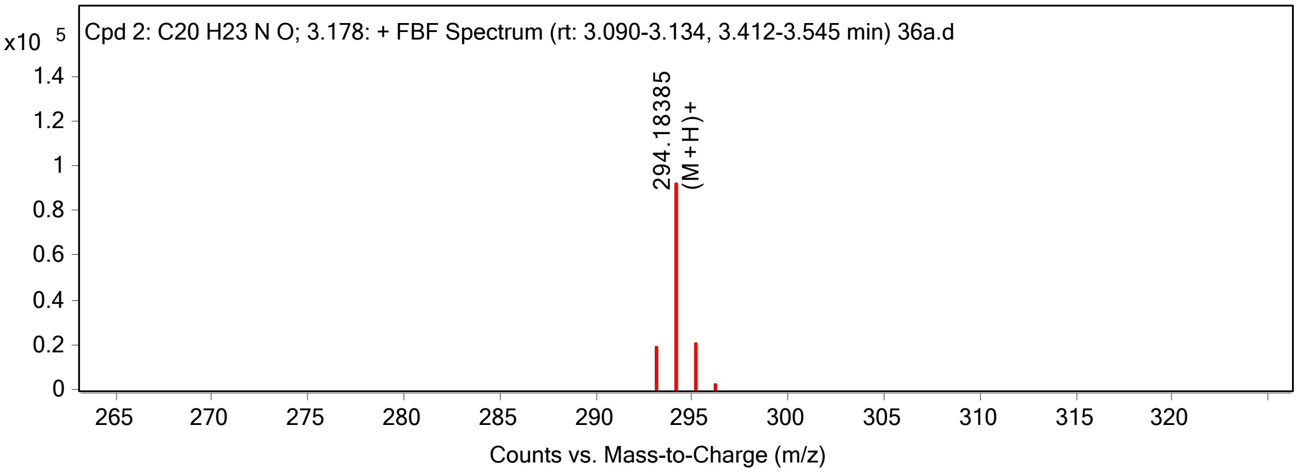

MS Spectrum Peak List

Obs. *m/z*	Charge	Abund	Ion/Isotope
293.1767	1	18954.4	M+
294.18385	1	92285.8	(M+H)+
295.18804	1	18990.9	(M+H)+
296.19221	1	2250.06	(M+H)+

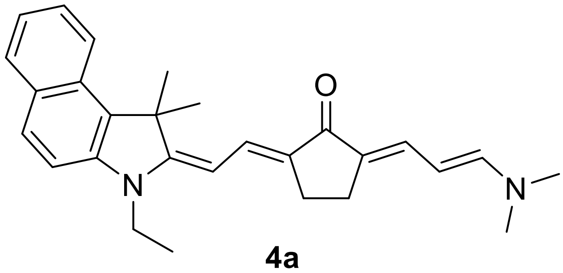

4a

Compound Table

Label	Tgt Score	Mass Error (ppm)	Tgt Formula	Obs. RT	Ref. Mass	Obs. Mass
Cpd 1: C28 H32 N2 O; 2.830	99.2	-4.58	C28 H32 N2 O	2.83	412.25146	412.24958

Obs. *m/z*	Obs. RT	Obs. Mass	Tgt Formula	Tgt Mass	Tgt Mass Error (ppm)	RT Diff.	Find Cpds Algorithm
413.25805	2.83	412.24958	C28 H32 N2 O	412.25146	-4.58	Find By Formula	

Compound Chromatograms

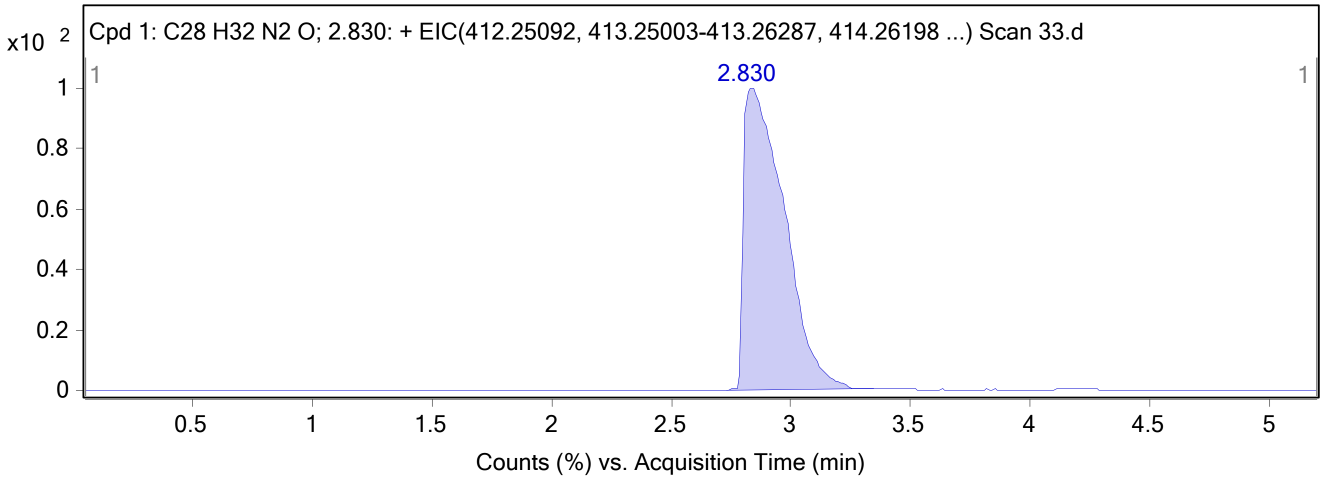

MS Zoomed Spectrum

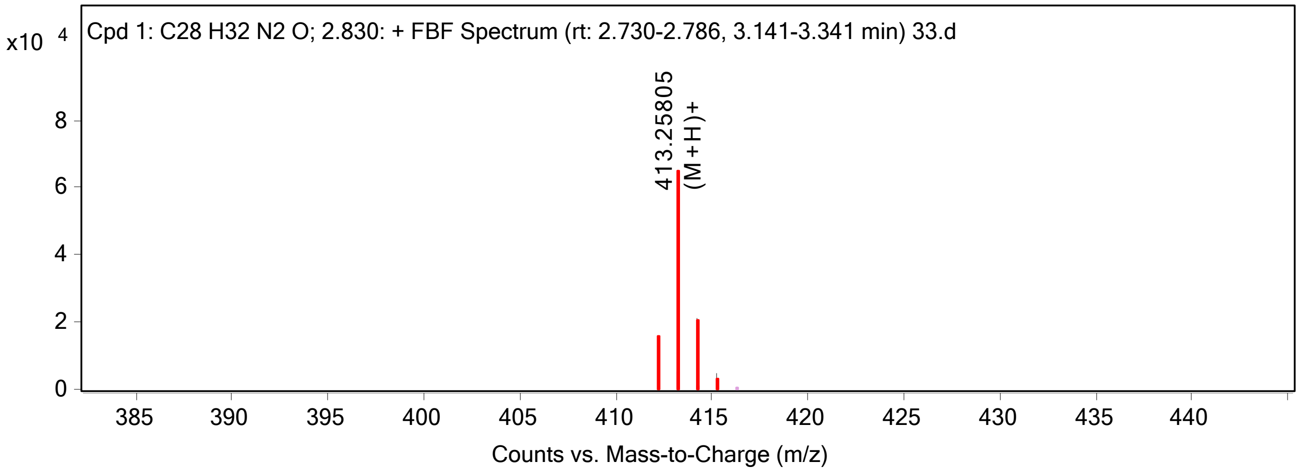

MS Spectrum Peak List

Obs. *m/z*	Charge	Abund	Ion/Isotope
412.25025	1	16023.18	M+
413.25805	1	62584.09	(M+H)+
414.25705	1	21404.27	(M+H)+
415.25713	1	4804.47	(M+H)+

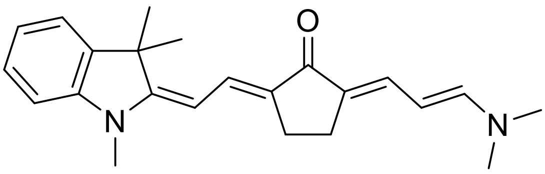

4b

Compound Table

Label	Tgt Score	Mass Error (ppm)	Tgt Formula	Obs. RT	Ref. Mass	Obs. Mass
Cpd 1: C23 H28 N2 O; 2.543	97.34	-2.56	C23 H28 N2 O	2.543	348.22016	348.21927

Obs. *m/z*	Obs. RT	Obs. Mass	Tgt Formula	Tgt Mass	Tgt Mass Error (ppm)	RT Diff.	Find Cpds Algorithm
349.22704	2.543	348.21927	C23 H28 N2 O	348.22016	-2.56	Find By Formula	

Compound Chromatograms

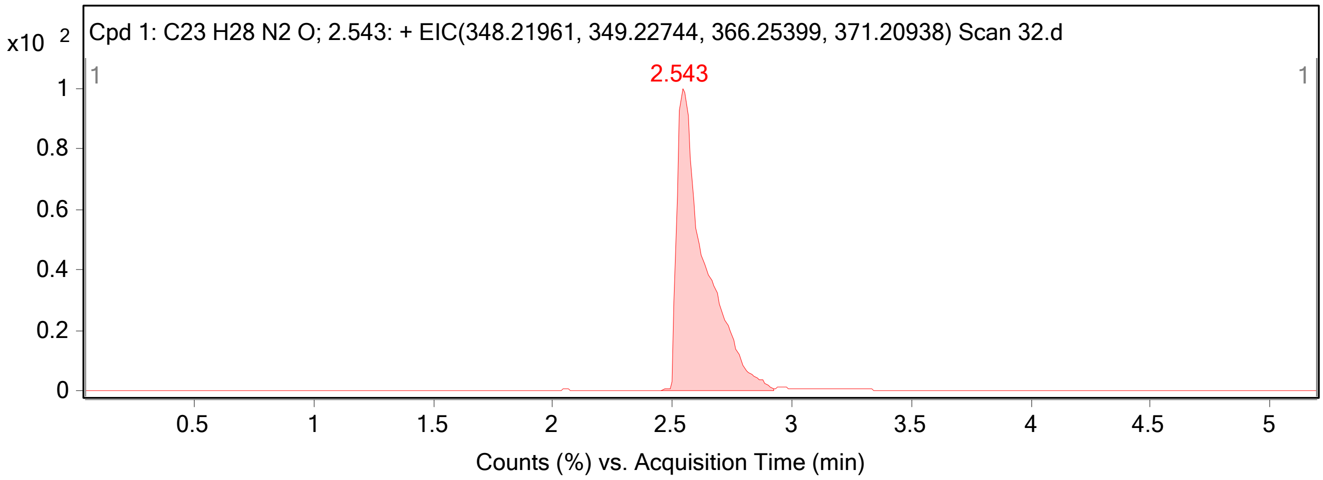

MS Zoomed Spectrum

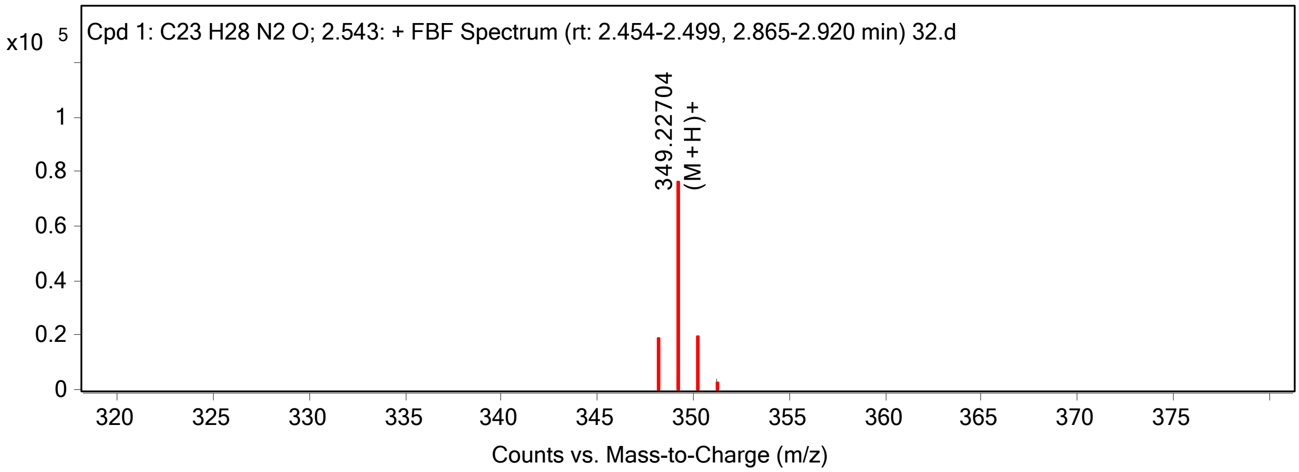

MS Spectrum Peak List

Obs. *m/z*	Charge	Abund	Ion/Isotope
348.21921	1	18733.21	M+
349.22704	1	76408.77	(M+H)+
350.22938	1	18545.08	(M+H)+
351.22208	1	3690.16	(M+H)+

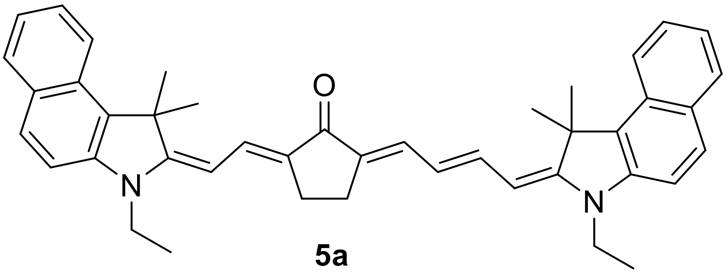

5a

Compound Table

Label	Tgt Score	Mass Error (ppm)	Tgt Formula	Obs. RT	Ref. Mass	Obs. Mass
Cpd 1: C43 H44 N2 O; 3.974	98.68	-2.48	C43 H44 N2 O	3.974	604.34536	604.34387

Obs. *m/z*	Obs. RT	Obs. Mass	Tgt Formula	Tgt Mass	Tgt Mass Error (ppm)	RT Diff.	Find Cpds Algorithm
604.34163	3.974	604.34387	C43 H44 N2 O	604.34536	-2.48	Find By Formula	

Compound Chromatograms

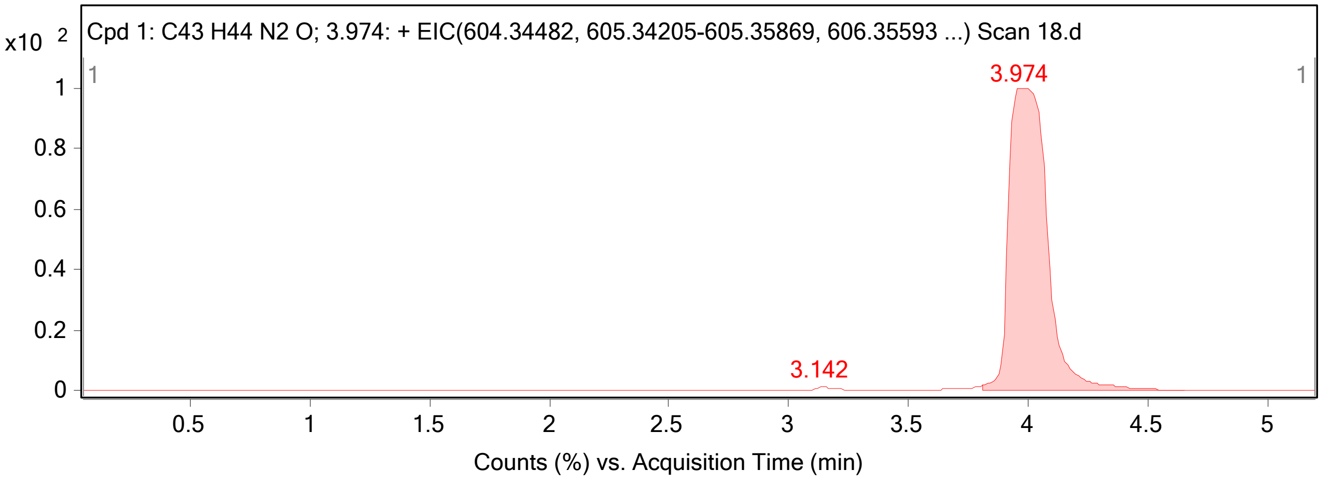

MS Zoomed Spectrum

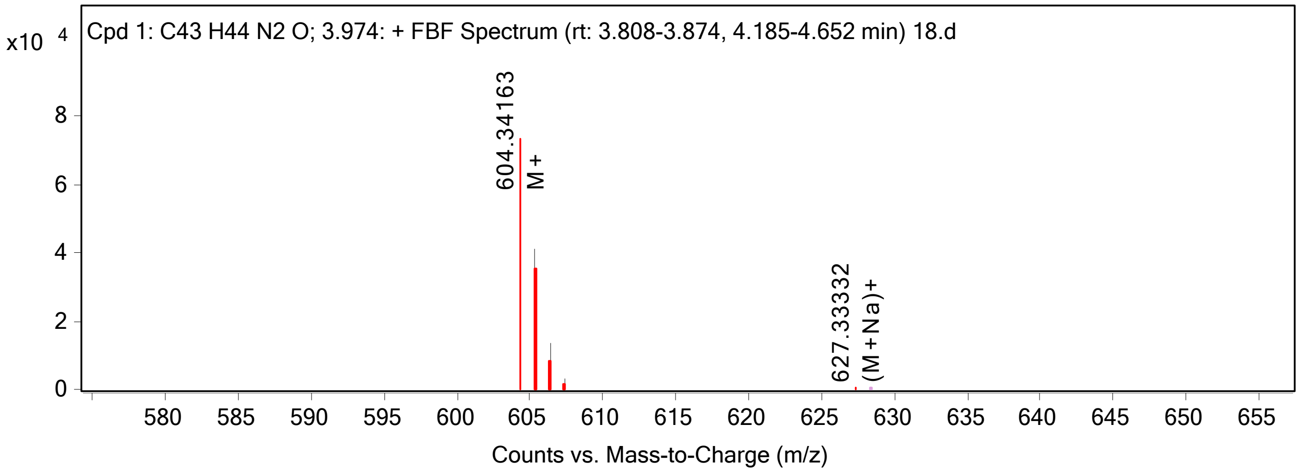

Obs. *m/z*	Charge	Abund	Ion/Isotope
604.34163	1	60204.38	M+
605.34846	1	41254.68	M+
606.35231	1	13699.16	M+
607.35056	1	3349.22	M+
627.33332	1	667.74	(M+Na)+

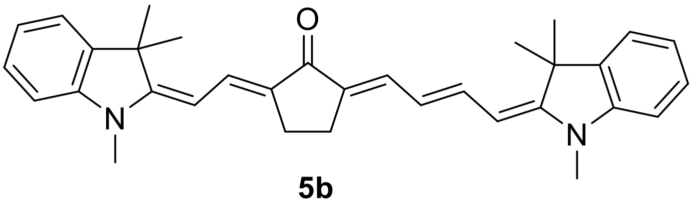

5b

Compound Table

Label	Tgt Score	Mass Error (ppm)	Tgt Formula	Obs. RT	Ref. Mass	Obs. Mass
Cpd 1: C33 H36 N2 O; 3.481	98.27	-3.85	C33 H36 N2 O	3.481	476.28276	476.28093

Obs. *m/z*	Obs. RT	Obs. Mass	Tgt Formula	Tgt Mass	Tgt Mass Error (ppm)	RT Diff.	Find Cpds Algorithm
477.2882	3.481	476.28093	C33 H36 N2 O	476.28276	-3.85	Find By Formula	

Compound Chromatograms

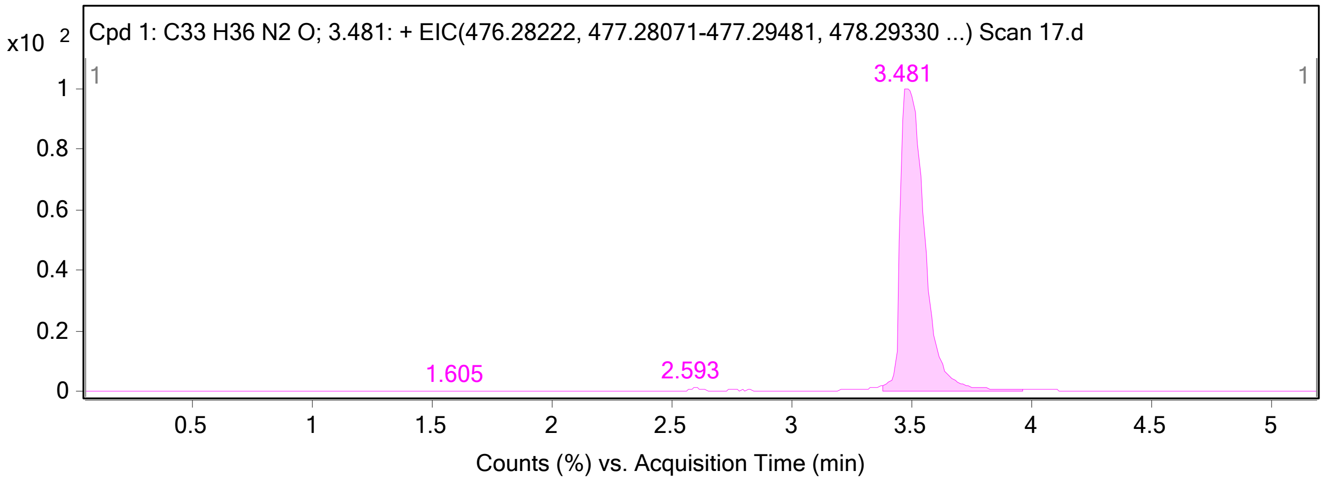

MS Zoomed Spectrum

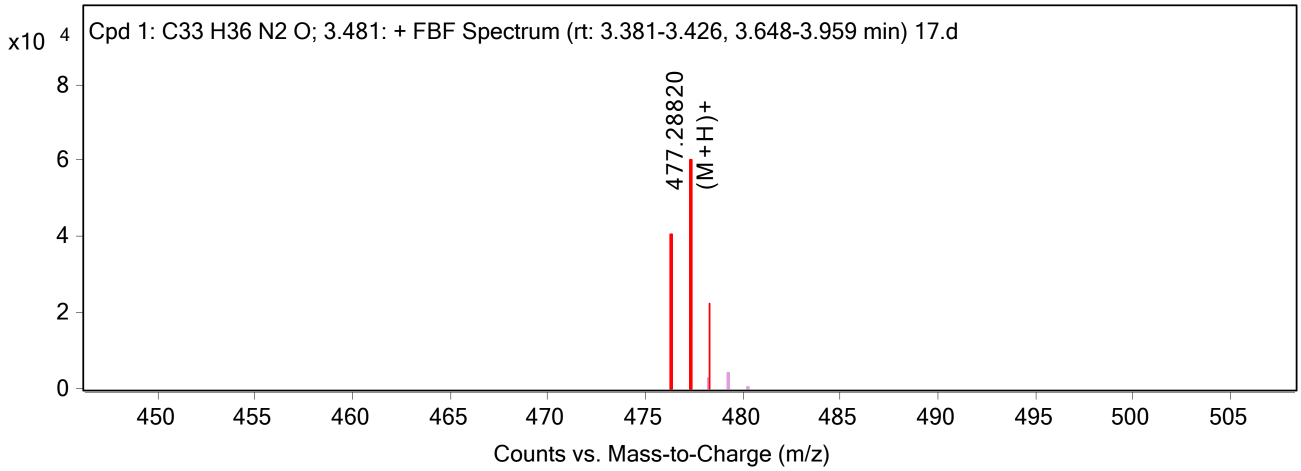

MS Spectrum Peak List

Obs. *m/z*	Charge	Abund	Ion/Isotope
476.2802	1	40691.73	M+
477.2882	1	60371.11	(M+H)+
478.29188	1	18717.99	(M+H)+

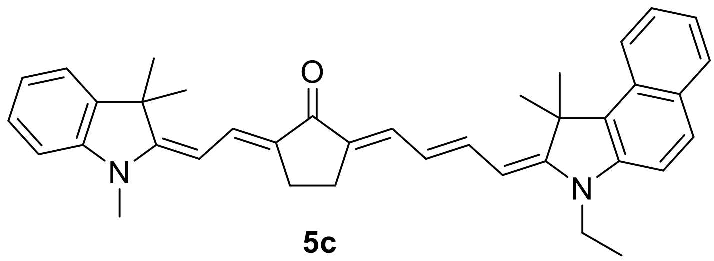

5c

Compound Table

Label	Tgt Score	Mass Error (ppm)	Tgt Formula	Obs. RT	Ref. Mass	Obs. Mass
Cpd 1: C38 H40 N2 O; 4.536	98.65	-4.73	C38 H40 N2 O	4.536	540.3141	540.3115

Obs. *m/z*	Obs. RT	Obs. Mass	Tgt Formula	Tgt Mass	Tgt Mass Error (ppm)	RT Diff.	Find Cpds Algorithm
541.3193	4.536	540.3115	C38 H40 N2 O	540.3141	-4.73	Find By Formula	

Compound Chromatograms

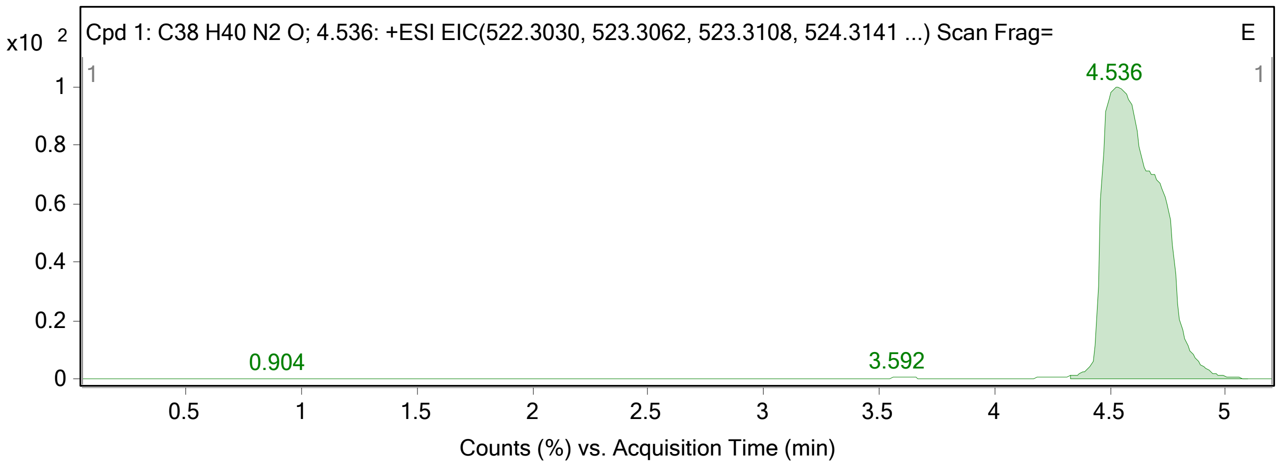

MS Zoomed Spectrum

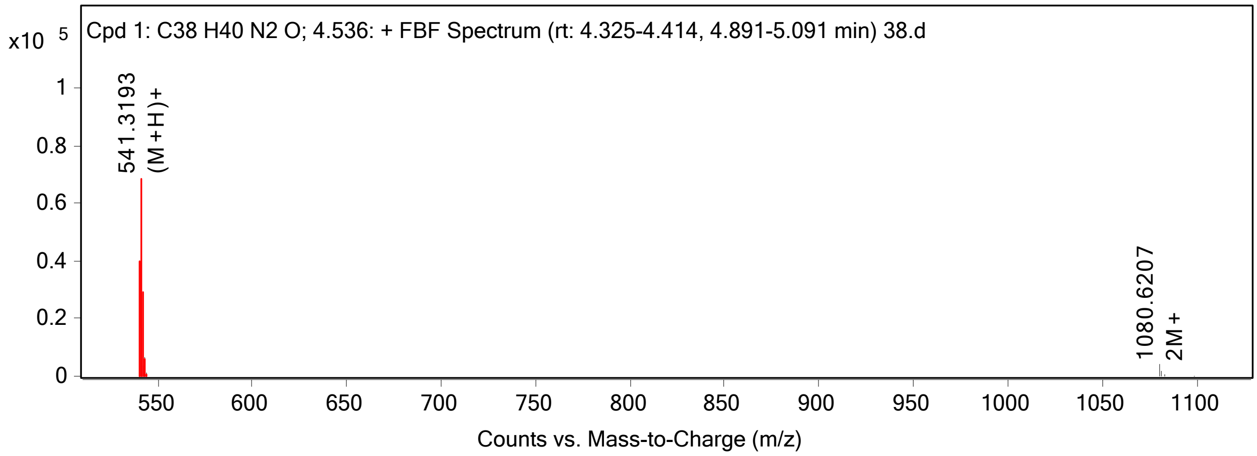

MS Spectrum Peak List

Obs. *m/z*	Charge	Abund	Ion/Isotope
540.3122	1	39805.49	M+
541.3193	1	68751.88	(M+H)+
542.3217	1	24942.31	(M+H)+
543.3201	1	6359.73	(M+H)+
544.3174	1	1101.91	(M+H)+
1080.6207	1	3928.14	2M+
1081.6228	1	1720.28	2M+
1082.625	1	637.99	2M+
1098.6547	1	105.56	(2M+NH4)+

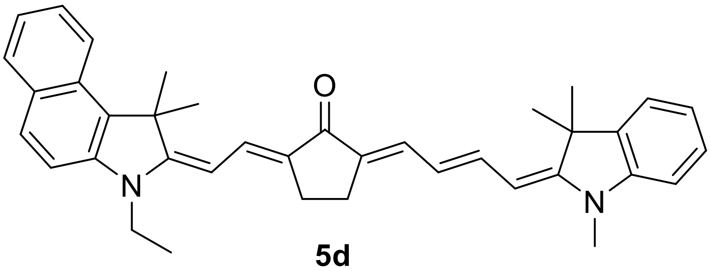

5d

Compound Table

Label	Tgt Score	Mass Error (ppm)	Tgt Formula	Obs. RT	Ref. Mass	Obs. Mass
Cpd 1: C38 H40 N2 O; 4.496	98.74	-4.36	C38 H40 N2 O	4.496	540.3141	540.3117

Obs. *m/z*	Obs. RT	Obs. Mass	Tgt Formula	Tgt Mass	Tgt Mass Error (ppm)	RT Diff.	Find Cpds Algorithm
541.3195	4.496	540.3117	C38 H40 N2 O	540.3141	-4.36	Find By Formula	

Compound Chromatograms

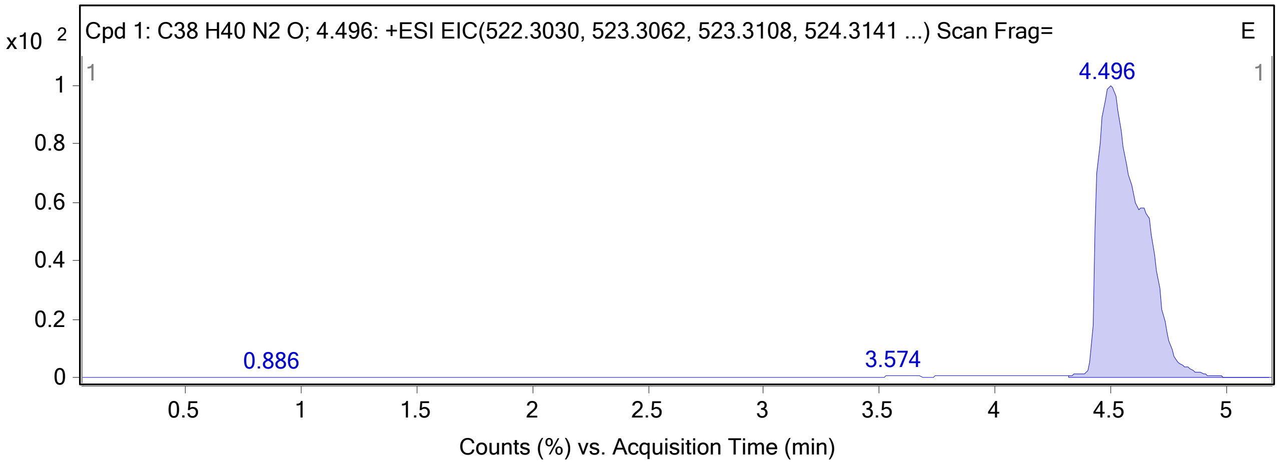

MS Zoomed Spectrum

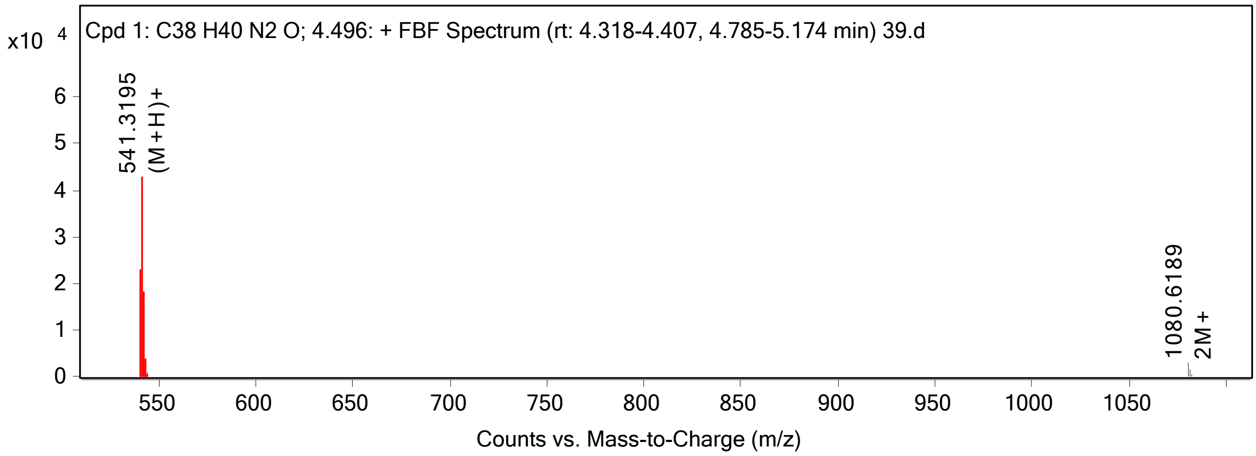

MS Spectrum Peak List

Obs. *m/z*	Charge	Abund	Ion/Isotope
540.312	1	23041.62	M+
541.3195	1	42654.46	(M+H)+
542.3228	1	15189.36	(M+H)+
543.3233	1	3680.05	(M+H)+
544.3222	1	624.85	(M+H)+
1080.6189	1	2821.99	2M+
1081.6199	1	1351.41	2M+
1082.6201	1	514.52	2M+

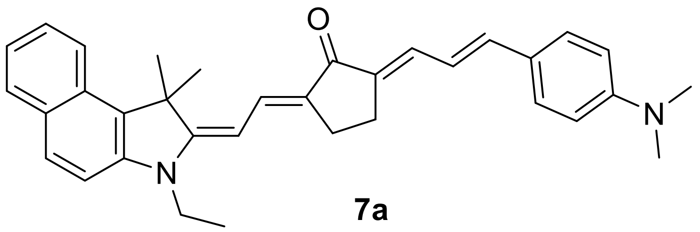

7a

Compound Chromatograms

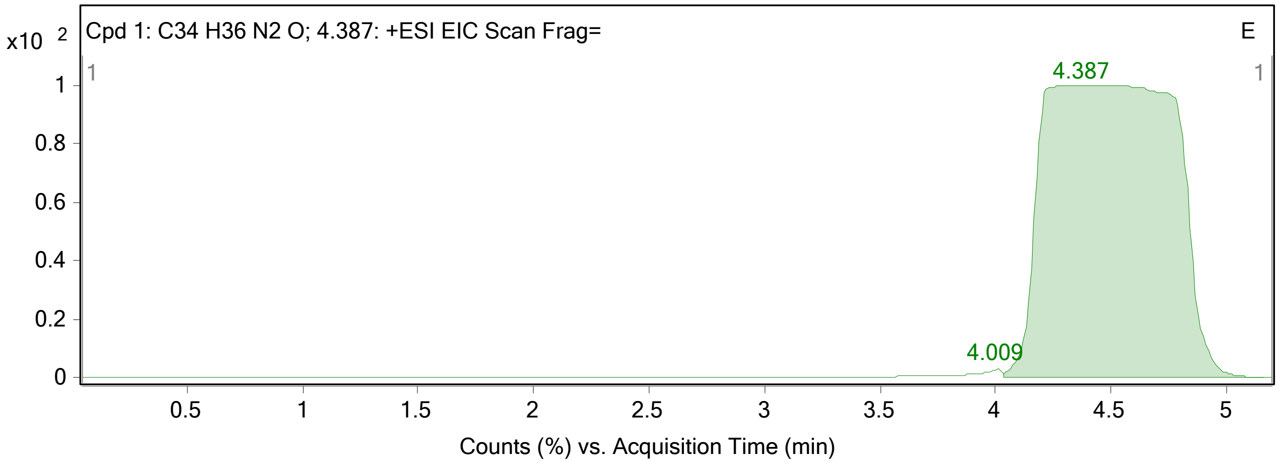

Spectra

Fragmentor Voltage	Collision Energy	Ionization Mode
120	0	ESI

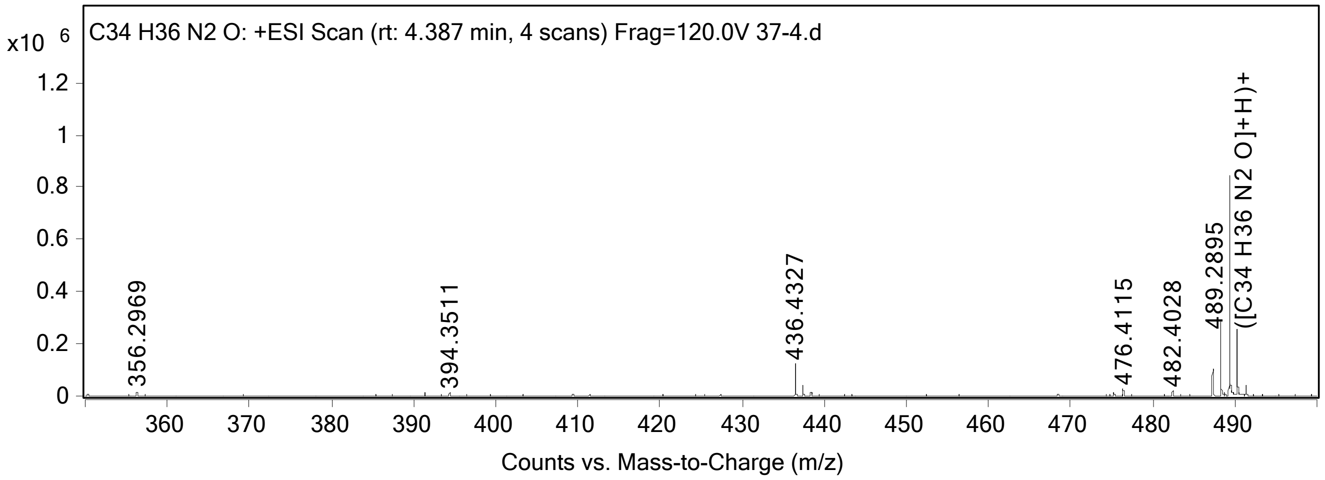

Peak List

m/z	z	Abund	Formula	Ion
121.0508	1	356066.92		
223.1354	1	230683.99		
245.1491	2	927238.21		
245.6505	2	314006.53		
488.2811		288102.47	C34 H36 N2 O	M+
489.2895	1	854425.53	C34 H36 N2 O	(M+H)+
490.2927	1	258251.68	C34 H36 N2 O	(M+H)+
521.2795	1	413242.54		
522.2827	1	139067.35		
922.0098	1	264562		

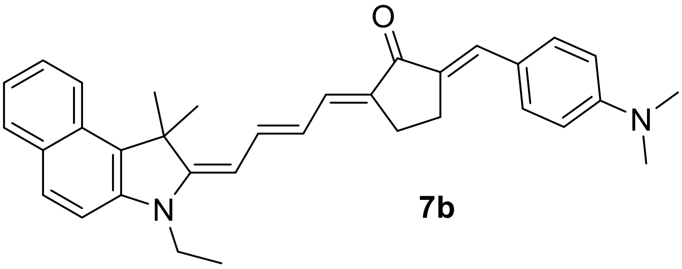

7b

Compound Table

Label	Tgt Score	Mass Error (ppm)	Tgt Formula	Obs. RT	Ref. Mass	Obs. Mass
Cpd 1: C34 H36 N2 O; 4.510	99.27	-1.74	C34 H36 N2 O	4.51	488.2828	488.2819

Obs. *m/z*	Obs. RT	Obs. Mass	Tgt Formula	Tgt Mass	Tgt Mass Error (ppm)	RT Diff.	Find Cpds Algorithm
489.2892	4.51	488.2819	C34 H36 N2 O	488.2828	-1.74	Find By Formula	

Compound Chromatograms

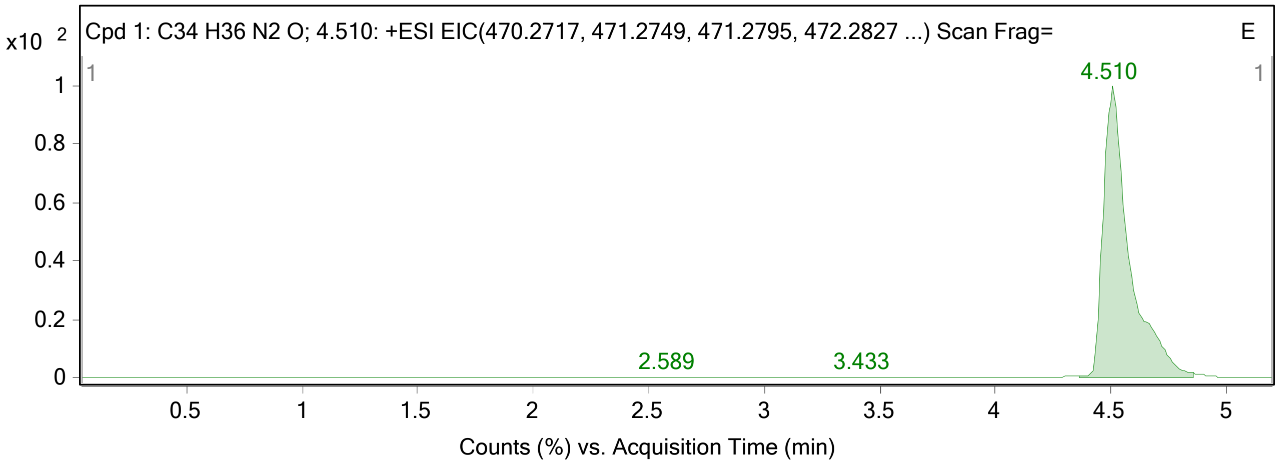

MS Zoomed Spectrum

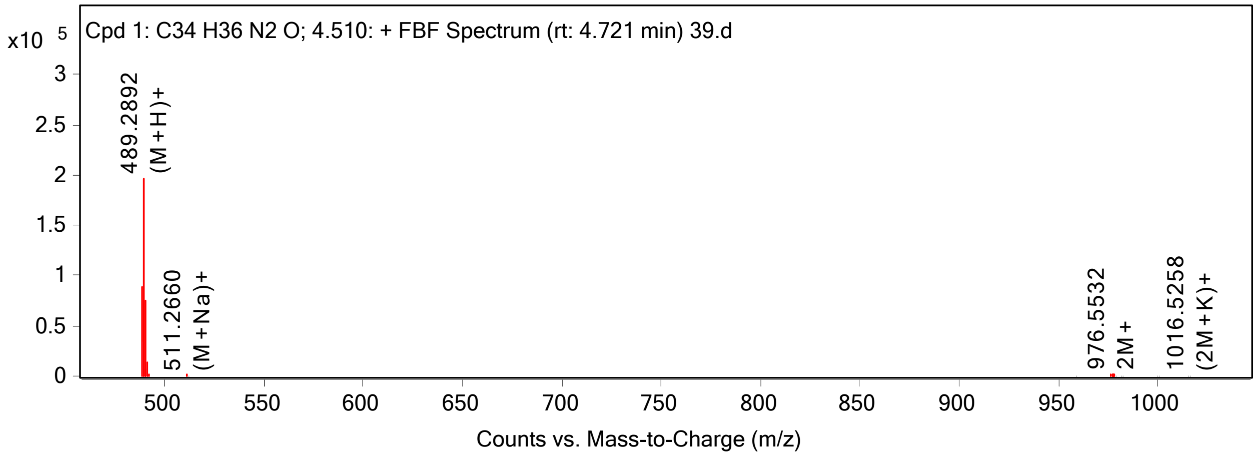

MS Spectrum Peak List

Obs. *m/z*	Charge	Abund	Ion/Isotope
488.2814	1	89226.56	M+
489.2892	1	196664.59	(M+H)+
490.2926	1	57612.74	(M+H)+
491.2962	1	11214.79	(M+H)+
492.2995	1	1581.71	(M+H)+
511.266	1	843.01	(M+Na)+
512.2764	1	522.01	(M+Na)+
976.5532	1	2014.15	2M+
977.5606	1	991.01	2M+
978.5548	1	338.4	2M+

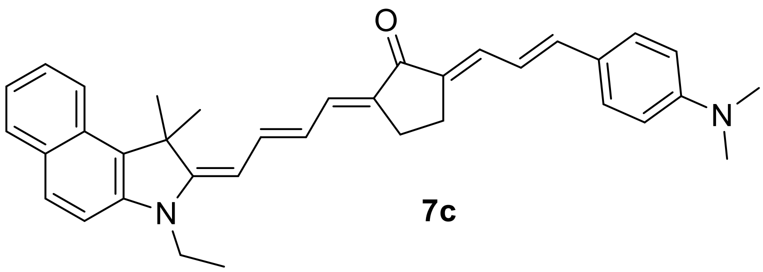

7c

Compound Table

Label	Tgt Score	Mass Error (ppm)	Tgt Formula	Obs. RT	Ref. Mass	Obs. Mass
Cpd 2: C36 H38 N2 O; 3.922	71.14	-1.98	C36 H38 N2 O	3.922	514.29841	514.29739

Obs. *m/z*	Obs. RT	Obs. Mass	Tgt Formula	Tgt Mass	Tgt Mass Error (ppm)	RT Diff.	Find Cpds Algorithm
515.30456	3.922	514.29739	C36 H38 N2 O	514.29841	-1.98	Find By Formula	

Compound Chromatograms

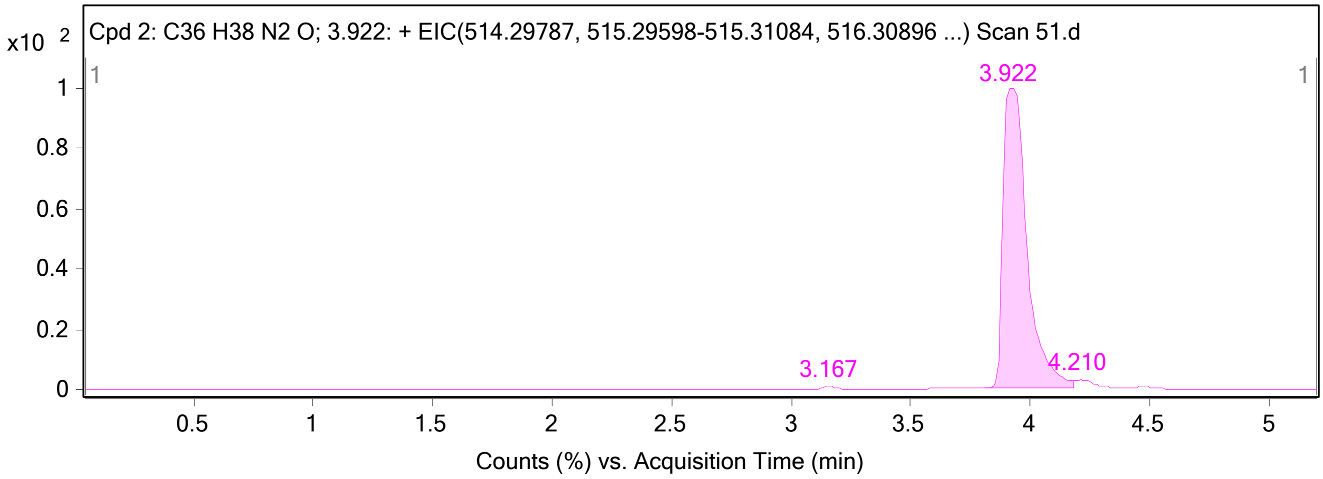

MS Zoomed Spectrum

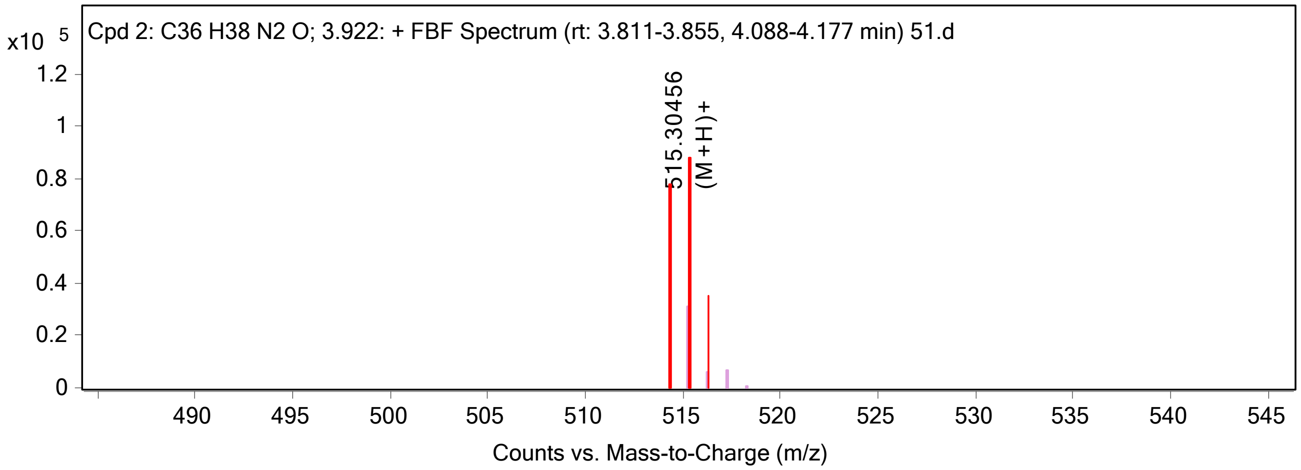

MS Spectrum Peak List

Obs. *m/z*	Charge	Abund	Ion/Isotope
514.29661	1	78056.5	M+
515.30456	1	87741.19	(M+H)+
516.30893	1	27778.92	(M+H)+

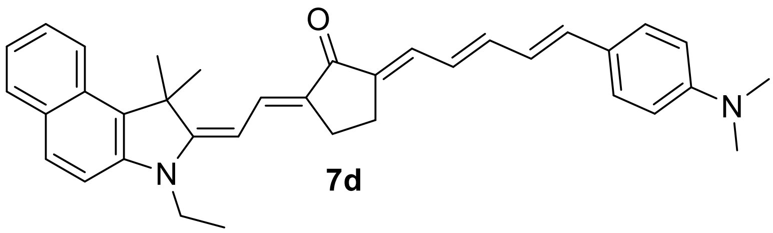

7d

Compound Table

Label	Tgt Score	Mass Error (ppm)	Tgt Formula	Obs. RT	Ref. Mass	Obs. Mass
Cpd 1: C36 H38 N2 O; 3.862	96.73	-1.27	C36 H38 N2 O	3.862	514.29841	514.29776

Obs. *m/z*	Obs. RT	Obs. Mass	Tgt Formula	Tgt Mass	Tgt Mass Error (ppm)	RT Diff.	Find Cpds Algorithm
515.30501	3.862	514.29776	C36 H38 N2 O	514.29841	-1.27	Find By Formula	

Compound Chromatograms

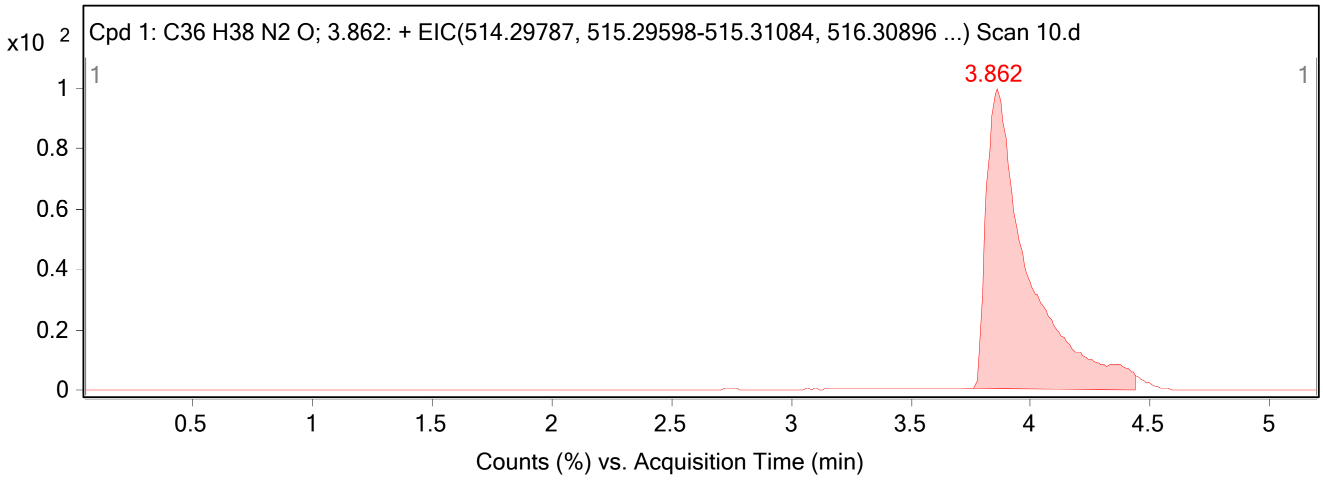

MS Zoomed Spectrum

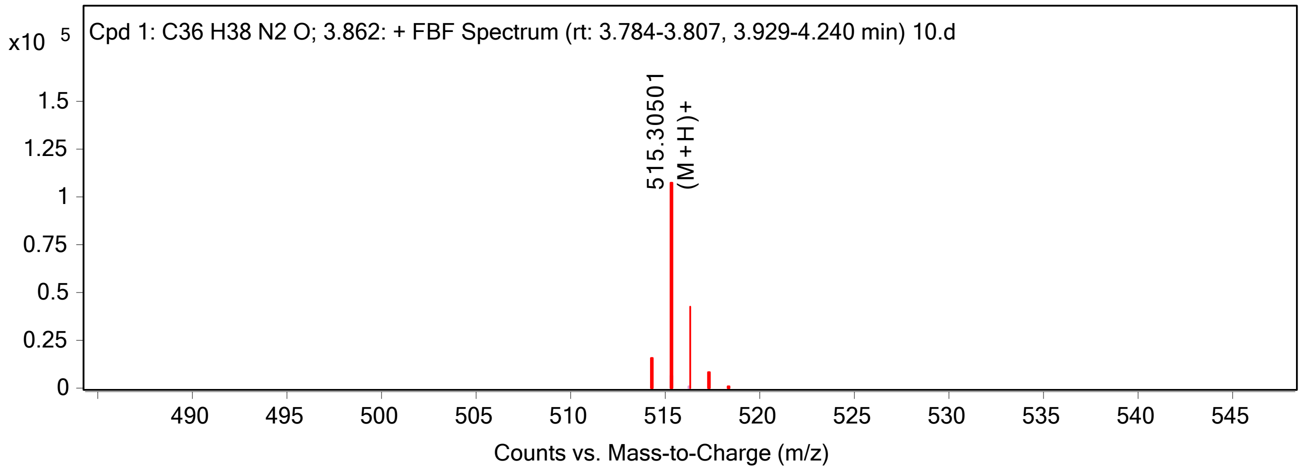

MS Spectrum Peak List

Obs. *m/z*	Charge	Abund	Ion/Isotope
514.29602	1	16179.56	M+
515.30501	1	107233.98	(M+H)+
516.30832	1	38805.86	(M+H)+
517.31354	1	7839.83	(M+H)+
518.31952	1	1207.94	(M+H)+

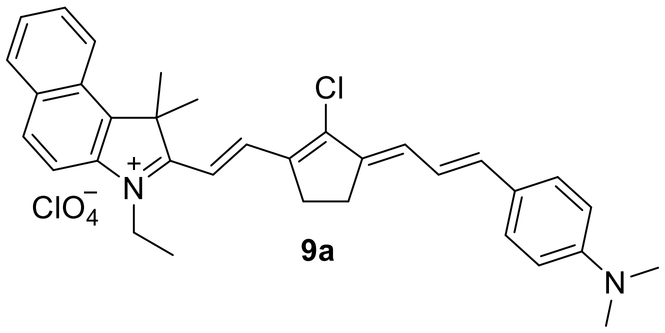

9a

Compound Table

Label	Tgt Score	Mass Error (ppm)	Tgt Formula	Obs. RT	Ref. Mass	Obs. Mass
Cpd 1: C34 H36 Cl N2; 3.368	94.78	1.33	C34 H36 Cl N2	3.368	507.2567	507.2574

Obs. *m/z*	Obs. RT	Obs. Mass	Tgt Formula	Tgt Mass	Tgt Mass Error (ppm)	RT Diff.	Find Cpds Algorithm
507.2563	3.368	507.2574	C34 H36 Cl N2	507.2567	1.33	Find By Formula	

Compound Chromatograms

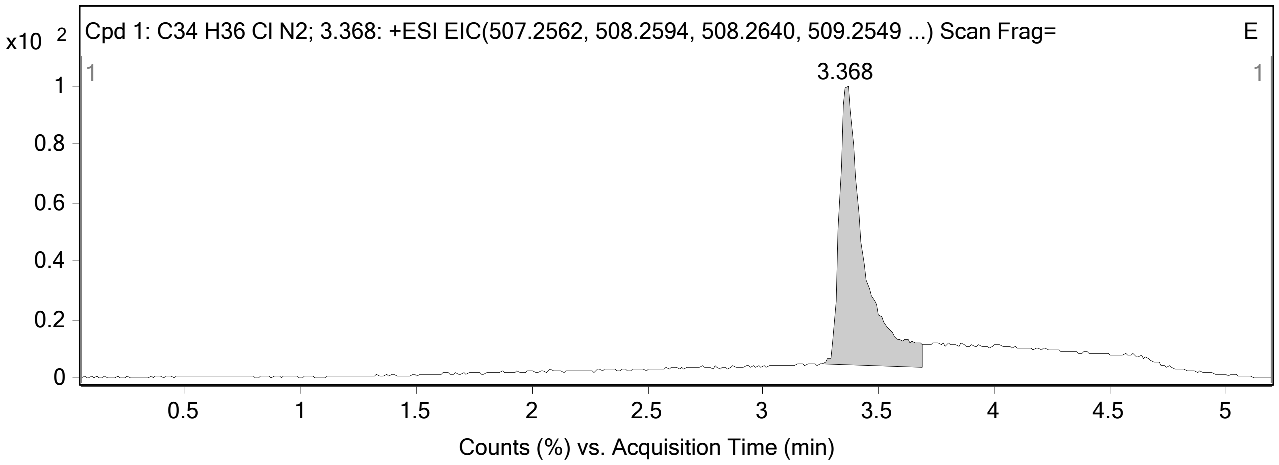

MS Zoomed Spectrum

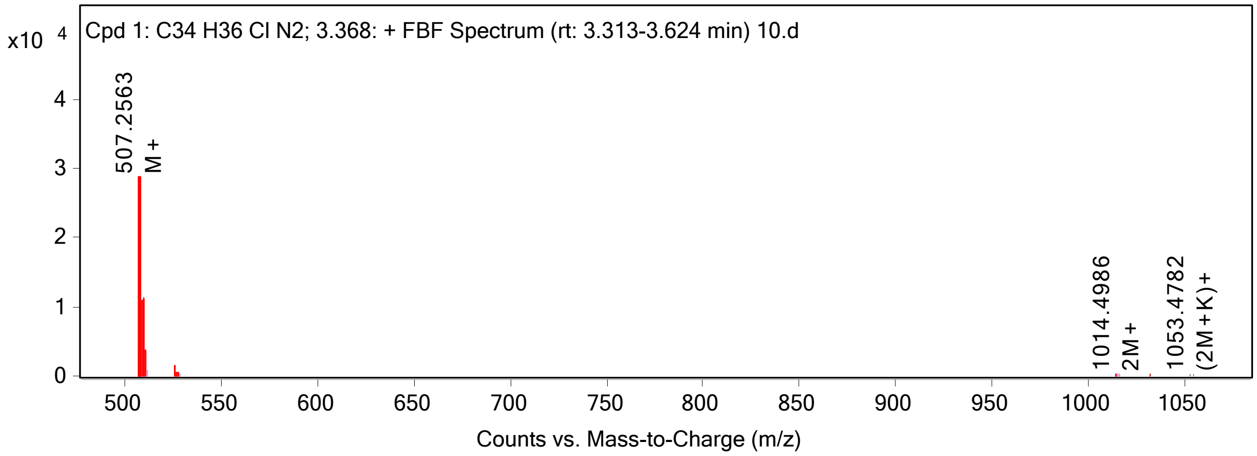

MS Spectrum Peak List

Obs. *m/z*	Charge	Abund	Ion/Isotope
507.2563	1	28660.1	M+
508.2595	1	10870.25	M+
509.2581	1	11393.04	M+
510.2581	1	3727.74	M+
525.2859	1	1379.55	(M+NH4)+
526.3022	1	635.2	(M+NH4)+
527.278	1	539.51	(M+NH4)+
1014.4986	1	195.14	2M+
1053.4782	1	213.51	(2M+K)+
1054.4792	1	190.26	(2M+K)+

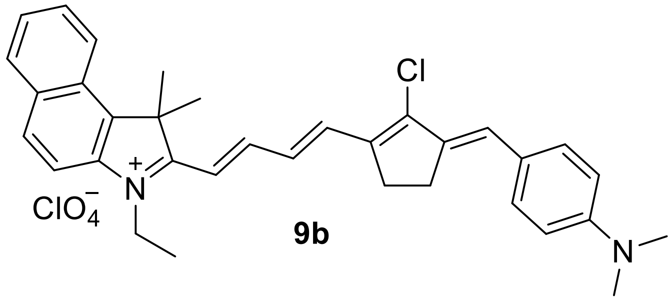

9b

Compound Table

Label	Tgt Score	Mass Error (ppm)	Tgt Formula	Obs. RT	Ref. Mass	Obs. Mass
Cpd 1: C34 H36 Cl N2; 3.959	96.12	0.54	C34 H36 Cl N2	3.959	507.2567	507.257

Obs. *m/z*	Obs. RT	Obs. Mass	Tgt Formula	Tgt Mass	Tgt Mass Error (ppm)	RT Diff.	Find Cpds Algorithm
507.2556	3.959	507.257	C34 H36 Cl N2	507.2567	0.54	Find By Formula	

Compound Chromatograms

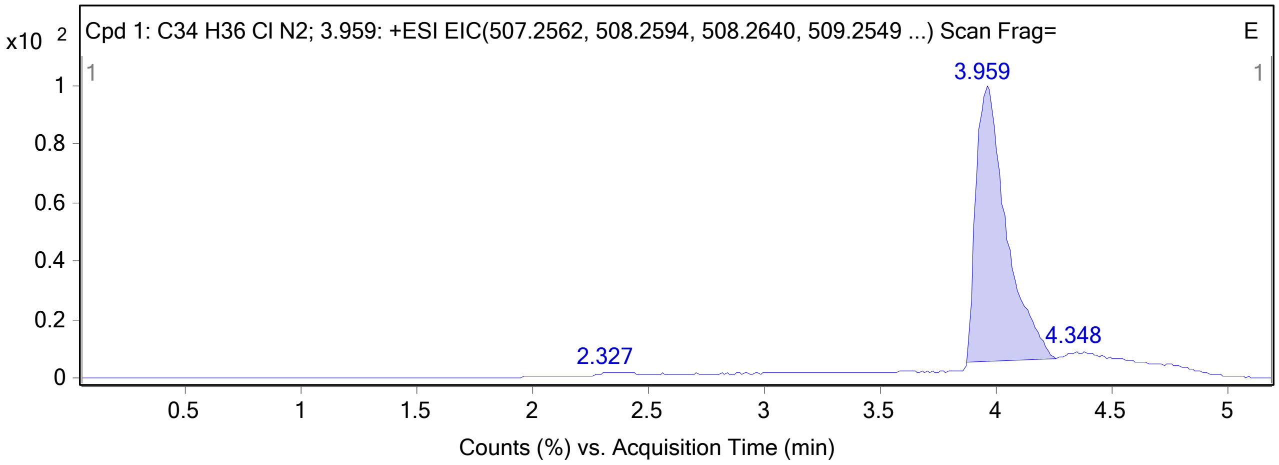

MS Zoomed Spectrum

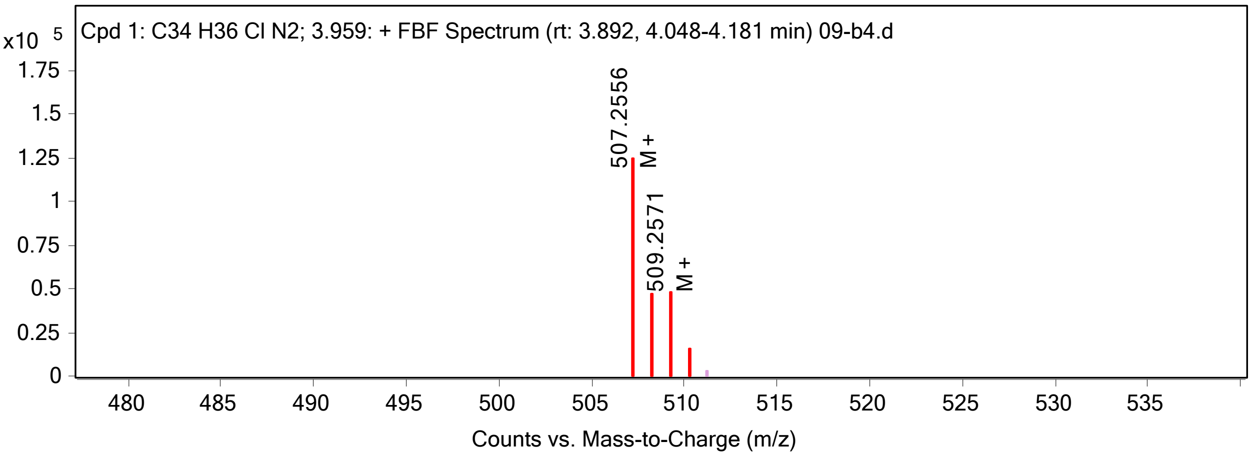

MS Spectrum Peak List

Obs. *m/z*	Charge	Abund	Ion/Isotope
507.2556	1	124402.63	M+
508.2596	1	44424.54	M+
509.2571	1	45817.75	M+
510.259	1	14950.01	M+

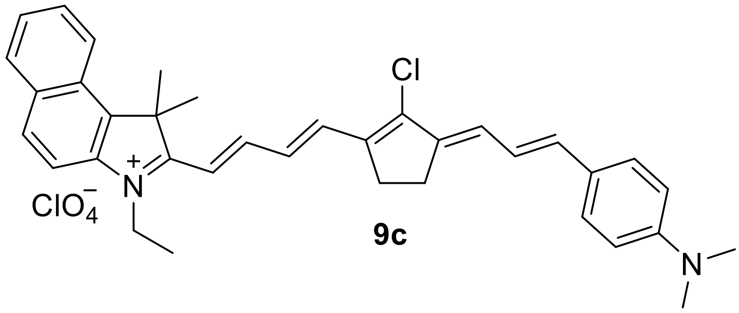

9c

Compound Table

Label	Tgt Score	Mass Error (ppm)	Tgt Formula	Obs. RT	Ref. Mass	Obs. Mass
Cpd 1: C36 H38 Cl N2; 3.851	82.26	-1.03	C36 H38 Cl N2	3.851	533.27235	533.2718

Obs. *m/z*	Obs. RT	Obs. Mass	Tgt Formula	Tgt Mass	Tgt Mass Error (ppm)	RT Diff.	Find Cpds Algorithm
533.27116	3.851	533.2718	C36 H38 Cl N2	533.27235	-1.03	Find By Formula	

Compound Chromatograms

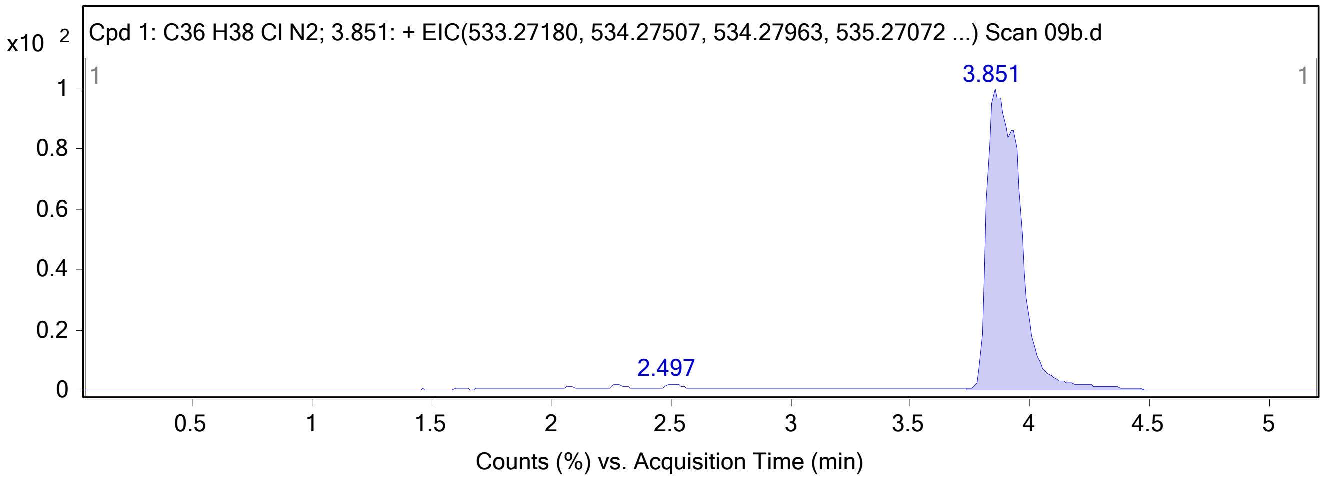

MS Zoomed Spectrum

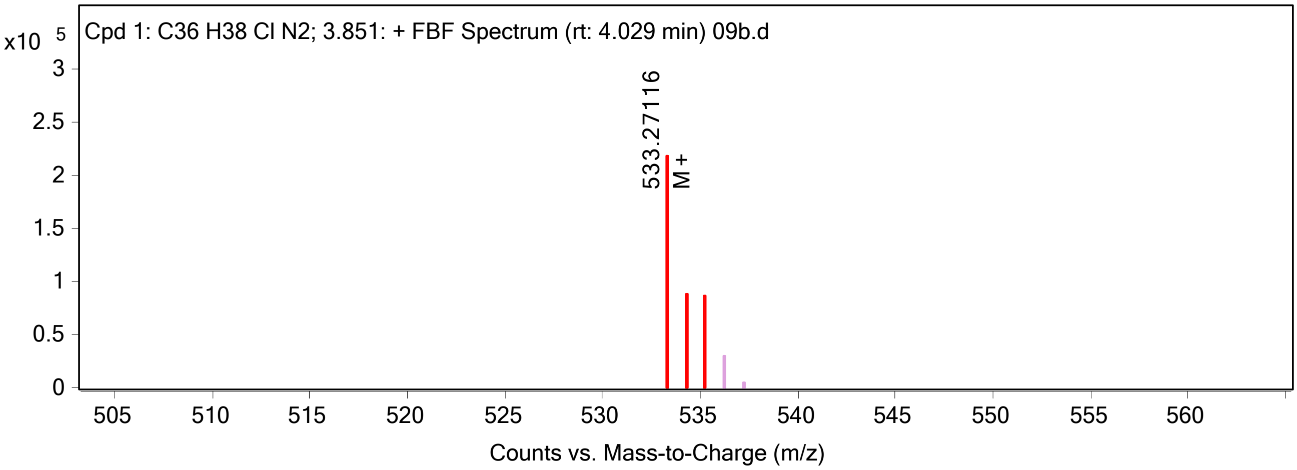

MS Spectrum Peak List

Obs. *m/z*	Charge	Abund	Ion/Isotope
533.27116	1	218961.78	M+
534.27463	1	76679.16	M+
535.27035	1	75918.51	M+

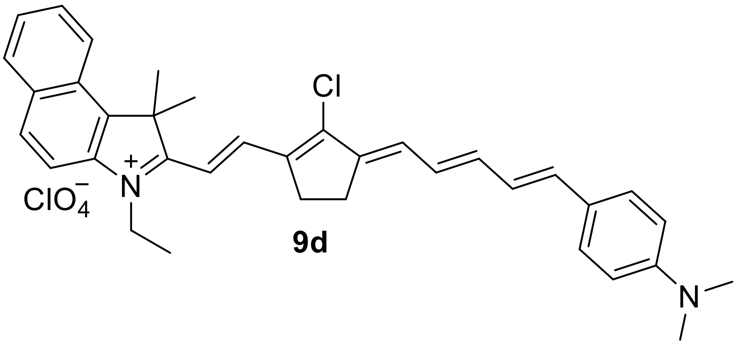

9d

Compound Table

Label	Tgt Score	Mass Error (ppm)	Tgt Formula	Obs. RT	Ref. Mass	Obs. Mass
Cpd 1: C36 H38 Cl N2; 3.851	82.26	-1.03	C36 H38 Cl N2	3.851	533.27235	533.2718

Obs. *m/z*	Obs. RT	Obs. Mass	Tgt Formula	Tgt Mass	Tgt Mass Error (ppm)	RT Diff.	Find Cpds Algorithm
533.27116	3.851	533.2718	C36 H38 Cl N2	533.27235	-1.03	Find By Formula	

Compound Chromatograms

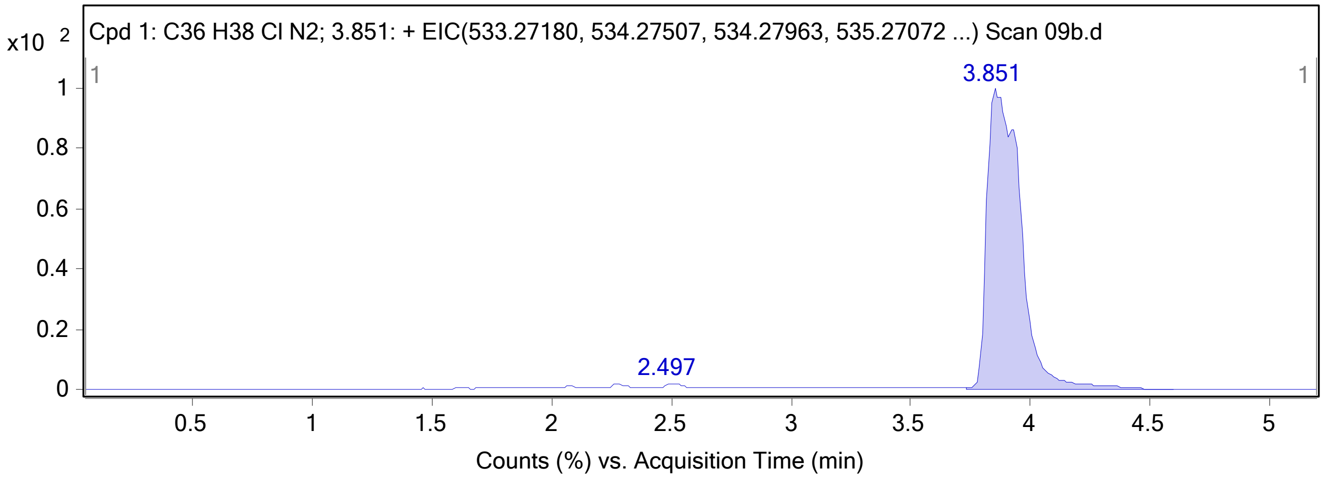

MS Zoomed Spectrum

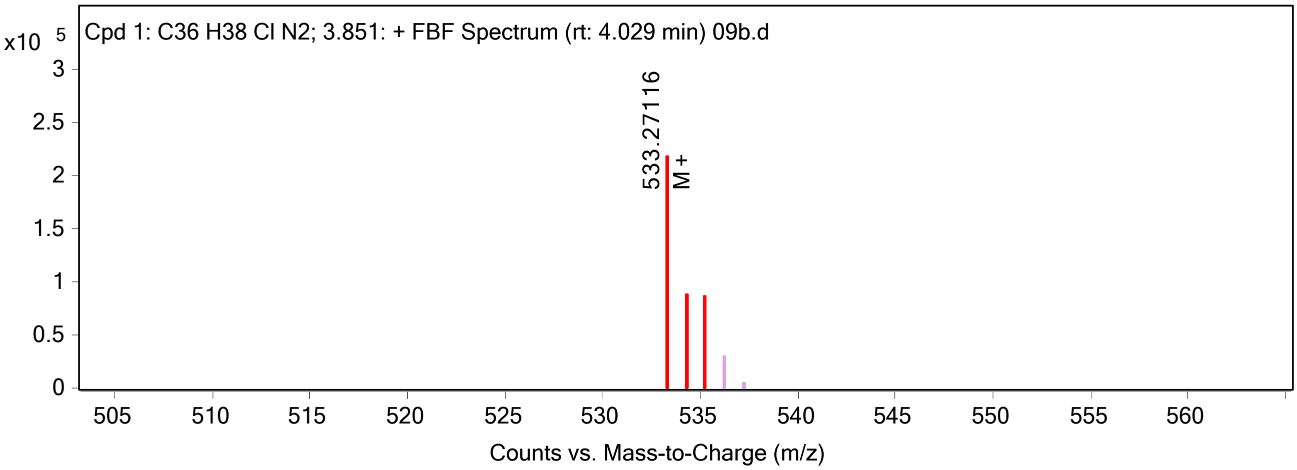

MS Spectrum Peak List

Obs. *m/z*	Charge	Abund	Ion/Isotope
533.27116	1	218961.78	M+
534.27463	1	76679.16	M+
535.27035	1	75918.51	M+

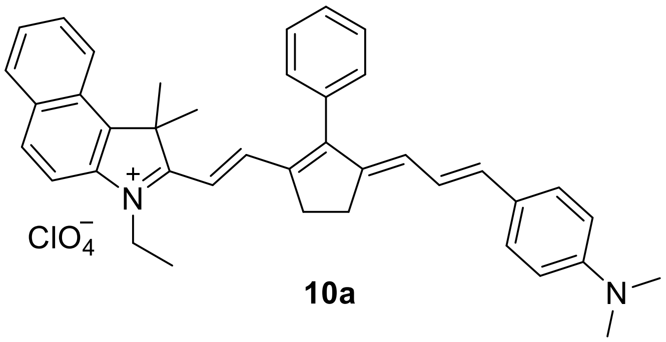

10a

Compound Summary

Cpd	Name	Formula	CAS	RT	Mass	Mass (Tgt)	Diff (Tgt, ppm)	Score	Algorithm
1		C40 H41 N2		4,616	549,3259	549,3270	-2,00	94,78	FBF

Compound Details

Cpd. 1: C40 H41 N2

Name	Formula	RT	RI	Mass	Diff (Tgt, ppm)	CAS	ID Source	Score	Algorithm
	C40 H41 N2	4,616		549,3259	-2,00		FBF	94,78	FBF

Species	m/z	Score (Tgt)	Score (Lib)	Score (DB)	Score (MFG)	Score (RT)
M+	549,3254	94,78				

Compound Chromatograms (overlaid)

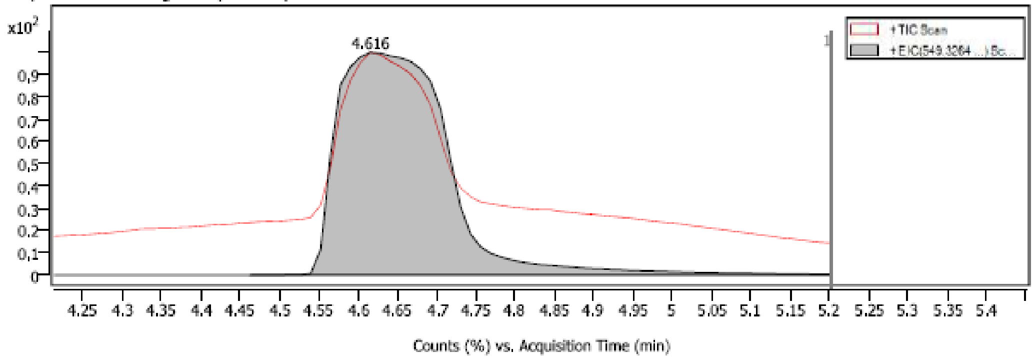

Structure

Compound Spectra (overlaid)

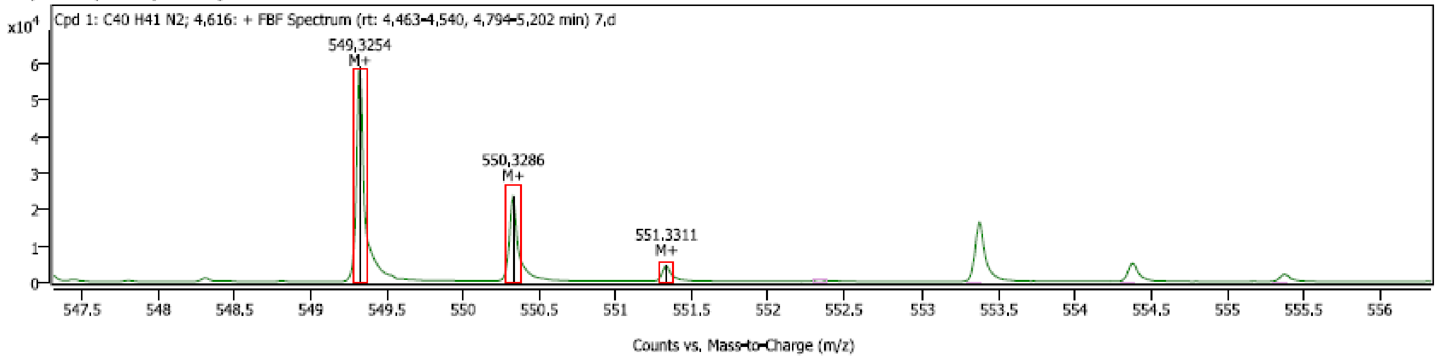

Compound ID Table

Name	Formula	Species	RT	RT Diff	Mass	CAS	ID Source	Score	Score (Lib)	Score (Tgt)
	C40 H41 N2	M+	4,616		549,3259		FBF	94,78		94,78

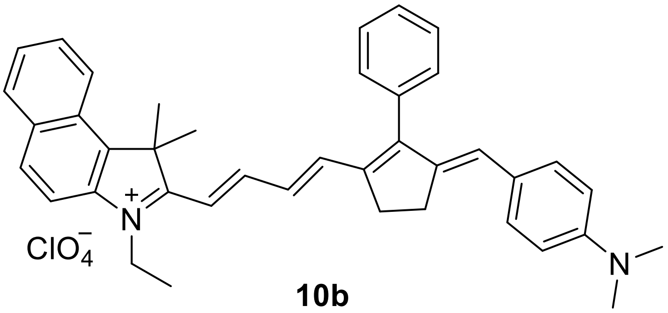

10b

Compound Summary

Cpd	Name	Formula	CAS	RT	Mass	Mass (Tgt)	Diff (Tgt, ppm)	Score	Algorithm
1		C40 H41 N2		4,482	549,3263	549,3270	-1,28	95,62	FBF

Compound Details

Cpd. 1: C40 H41 N2

Name	Formula	RT	RI	Mass	Diff (Tgt, ppm)	CAS	ID Source	Score	Algorithm
	C40 H41 N2	4,482		549,3263	-1,28		FBF	95,62	FBF

Species	m/z	Score (Tgt)	Score (Lib)	Score (DB)	Score (MFG)	Score (RT)
M+	549,3257	95,62				

Compound Chromatograms (overlaid)

Structure

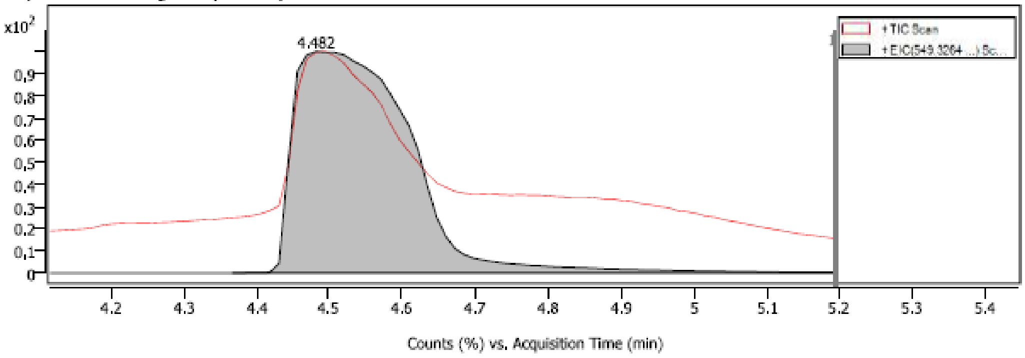

Compound Spectra (overlaid)

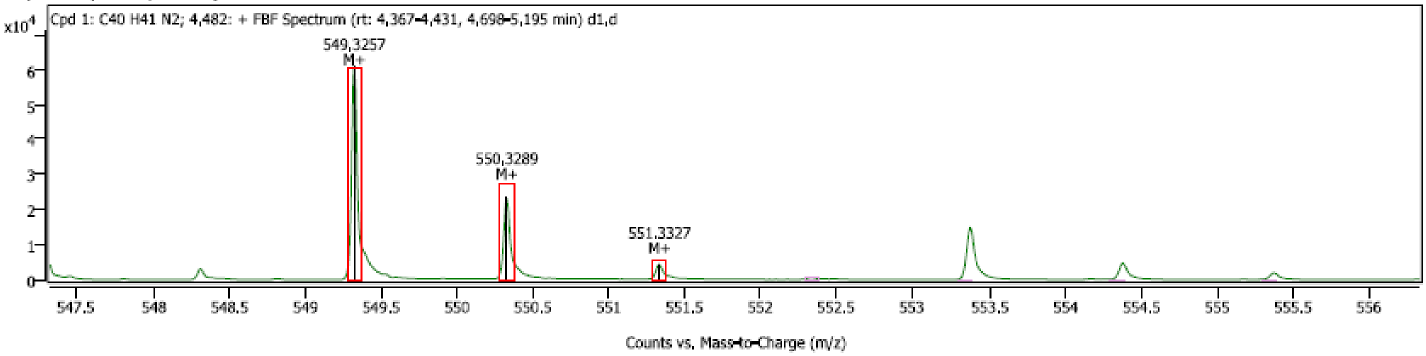

Compound ID Table

Name	Formula	Species	RT	RT Diff	Mass	CAS	ID Source	Score	Score (Lib)	Score (Tgt)
	C40 H41 N2	M+	4,482		549,3263		FBF	95,62		95,62
